# Digital health technologies for adults with ADHD: a scoping review

**DOI:** 10.3389/fdgth.2026.1746732

**Published:** 2026-02-23

**Authors:** Fin J. Schofield, Sarah Wilkie, Emily E. Nielsen, Amberly Brigden, Matt W. Jones, Hanna K. Isotalus

**Affiliations:** 1 Centre for Digital Health and Care, School of Engineering Maths and Technology, Faculty of Engineering and Science, University of Bristol, Bristol, United Kingdom; 2 School of Physiology, Pharmacology, and Neuroscience, Faculty of Life Sciences, University of Bristol, Bristol, United Kingdom

**Keywords:** adult ADHD, cognitive psychology, diagnostic technologies, digital interventions, mHealth, remote monitoring technologies

## Abstract

**Introduction:**

Adult Attention-Deficit/Hyperactivity Disorder (ADHD) is associated with negative long-term outcomes including accident and injury, impairment in social and occupational functioning, and a high rate of mental health comorbidities. Access to suitable healthcare remains challenging due to diagnostic delays, variable treatment responses, and difficulties transitioning out of pediatric support structures. Digital health technologies (DHTs) hold the potential to address these challenges.

**Methods:**

We conducted a scoping review to identify DHTs developed specifically for adults with ADHD, categorize them by their intended role within the health and social care system and by their core technological features, examine their methodological trends, and examine the quality of evidence by conducting a Risk of Bias analysis.

**Results:**

A systematic search across databases, up to December 2025, identified 133 eligible studies. 63 were categorized as *Treat a Specific Condition*, most frequently using web/app-based cognitive therapy or psychoeducation (*n* = 26), cognitive training programs (*n* = 13), transcranial stimulation (*n* = 12), and neurofeedback (*n* = 9). 36 were categorized as *Drive Clinical Management*, with technologies mostly supporting diagnostic decision-making through machine-learning analysis of participant features, such as data from continuous performance tasks (*n* = 11), neuroimaging (*n* = 11), and virtual reality (*n* = 5). 19 papers were classified as *Diagnose a Specific Condition* and used similar machine-learning classification, yet do not situate the DHT as a support tool that complements the traditional clinical assessment pathway.

**Discussion:**

Through our analysis, we identify various opportunities to strengthen the evidence base. This includes clarifying clinical integration points for diagnostic DHTs, ensuring technologies support adherence by incorporating lived experience, and developing remote monitoring technologies that demonstrate value to both clinicians and patients. Key questions remain on how DHTs can be translated into clinical practice, and we highlight various implementation-oriented frameworks which can guide development by encouraging multidisciplinary research that ensures the broader health and care system is considered alongside isolated measures of preliminary efficacy.

**Systematic Review Registration:**

https://osf.io/tk3pm.

## 1 Introduction

Attention-deficit/hyperactivity disorder (ADHD) is defined as a persistent neurodevelopmental disorder with presentations of hyperactivity, impulsivity, and inattention (1). The criteria for diagnosis center around the identification of persistent and pervasive symptoms of inattention and/or hyperactivity-impulsivity which result in functional impairment (2, 3).

Whilst historically viewed as a childhood disorder, longitudinal studies have identified that ADHD symptoms persist into adulthood in up to 65% of cases (4–7). Both the World Health Organization (WHO) and American Psychiatric Association (APA) have updated their criteria to recognize adult ADHD, which in turn has supported the development of clinical interview structures and screening tools for use when diagnosing adult ADHD (8–11). The prevalence of adulthood ADHD is estimated to be 2.58% (12).

Currently, diagnosis of adult ADHD is challenging, with reports in the UK of wait-times commonly exceeding 2 years (13), and an administrative prevalence (i.e., having a diagnosis coded in a primary care record) of 0.3% for adult males and 0.07% for adult females (14), which is substantially lower than the expected epidemiological prevalence. In the absence of established biomarkers for ADHD, diagnosis is determined by a detailed patient history, ideally collated from multiple reporters (15). However, referral into this process is prone to biases (16), delays (17), and consequently missed diagnoses (18). For those who receive a diagnosis in infancy, the transition into adulthood poses a risk as they move away from parental and clinical pediatric support structures (19). Examples show that only a fifth of those who require an adult mental health service referral successfully make the transition from pediatric services (20).

Alongside difficulties in diagnosis, ADHD in adulthood is reported to negatively affect an individual across multiple functional domains. Those diagnosed with ADHD are more prone to accidents (21), death by accident (22), academic underachievement (23), and impairment in social or occupational environments (24). In a meta-analysis, Shaw et al. (25), identify 9 major domains (non-therapeutic drug use, academic outcomes, antisocial behavior, social functioning, self-esteem, occupation, driving, services use, and obesity) wherein participants with ADHD were shown to have poorer long-term outcomes when compared to non-ADHD controls. Furthermore, in adulthood, ADHD is often comorbid with various other disorders, including anxiety disorders, major depressive disorder, bipolar disorder, and substance use disorder (26).

ADHD can be effectively managed, with evidence for the use of stimulants as first-line treatment in adulthood (27) alongside an array of non-pharmacological treatment paradigms that target specific symptoms (28). Whilst pharmacotherapies have been extensively trialed (27), inter-individual clinical response to stimulants vary (29), and clinical guidelines suggest regular monitoring and adjustment (15). Furthermore, evidence for the efficacy of stimulants for long-term outcomes is scarcer (25, 27). Whilst treatment with medication is more readily available, investigations have found that in the UK, psychological treatment was only available at half of ADHD services (30). Together, these challenges demonstrate a need to improve not only service provision, but further develop opportunities that empower patients to self-manage as traditional support structures diminish during adulthood.

Digital health technologies (DHTs), such as smartphone apps, wearable devices, and medical platforms, are increasingly being considered within healthcare systems (31–33), as they offer the potential to deliver cost benefits at scale to stretched services (32). DHTs additionally act as enablers of a more continuous form of health management that extends beyond classical clinical contexts (34). Unlike traditional treatment models, digital interventions can be adaptive to individual needs and preferences, which is in part enabled by the rapid and iterative frameworks which underpin technological development (35). DHTs span a wide range of uses, including the deployment of artificial intelligence (AI) to automate or support diagnosis (36), the delivery of digital health interventions (DHIs) that are intended to directly treat a condition through interactive behavioral or neurocognitive therapies (37), or remote monitoring technologies that gather and track patient outcomes (38).

Previous meta-reviews, such as Hollis et al. (37), collated reviews and randomized controlled trials (RCTs) that evaluate the clinical effectiveness of DHIs to improve mental health outcomes. They identified 190 papers evaluating approximately 147 DHIs, spanning multiple clinical targets including ADHD. Despite identifying 10 RCTs aimed at improving ADHD-related symptoms, they highlight inconsistent results for clinical efficacy. Lakes et al. (39), additionally performed a mapping review focused solely on DHIs developed for ADHD. They highlight rapid development within the field, yet note their included studies demonstrated little evidence of clinical efficacy from real-world settings. These findings demonstrate a need to identify where there is existing evidence for DHIs in the literature and examine possible sources of inconsistency within the evidence base. In addition, both these reviews have solely focused on interventional tools in childhood ADHD. As such, there is a need to assess the state of DHTs more broadly throughout the care pathway, whilst understanding their use in an adult ADHD population that is faced with a unique set of challenges in accessing suitable healthcare and for whom a “care gap” appears (19).

This review broadens the scope to examine how DHTs are developed and deployed throughout the diagnosis, management, and treatment of ADHD in adulthood. We apply this specifically to adulthood, and extend the scope beyond DHIs to consider how DHTs can be applied across the health and care system. Using a framework for DHT classification (40), we chart the evidence based on the DHT's intended purpose and primary modality, examine the outcome measures which are used to determine the efficacy, and employ a Risk of Bias (RoB) analysis to identify trends in sources of bias. The results of this enable us to formulate how future studies can address gaps in the evidence base and extend the utility of DHTs across domains of healthcare and support for adults with ADHD.

## 2 Methods

As a methodology for evidence synthesis, we chose to perform a scoping review as the method enables us to map a breadth of existing evidence across a range of sources, without limiting the objective to a single outcome or modality (41). We used guidance from the Joanna Briggs Institute (42) during the development of our protocol, which is available at https://osf.io/tk3pm. We report our findings in line with the Preferred Reporting Methods for Systematic Reviews and Meta-Analyses extension for Scoping Reviews (PRISMA-ScR) (43). The checklist for each criterion of the PRISMA-ScR is available in the Supplementary Material.

We first developed an *a priori* research question wherein we query the extent to which DHTs are being employed that target adults with ADHD. Broadly, there are four outcomes of interest from this review, which are to: (1) Conduct a comprehensive search to identify DHTS developed to specifically target adults with ADHD, (2) Chart identified DHTs by (a) classifying their intended purpose and (b) identifying their core technological features, (3) Examine methodological trends in the development and evaluation of DHTs, and (4) Examine the quality of evidence for identified DHTs by conducting a Risk of Bias analysis.

### 2.1 Data sources and search strategy

To reflect the interdisciplinary nature of digital health technologies, we deployed searches in a mixture of medical (PubMed, PsycINFO, Medline, CINAHL, Cochrane), engineering (IEEE Xplore, ACM Digital Library), and general-purpose databases (Web of Science, Scopus). Base terms “ADHD”, “Technology”, and “Adult” were used to initially scan titles and abstracts to identify related terminology. Where possible, we incorporated MeSH, APA, or key word descriptors to index relevant literature categories. Search terms deployed in PubMed are shown in Table 1, with all search queries available to view in the Supplementary Material.

**Table 1:** Search terms deployed for pubMed.

Terms connected by OR	AND	Terms connected by OR	AND	Terms connected by OR
Attention Deficit Disorder with Hyperactivity [MeSH]		Telemedicine [MeSH]		Adult [MeSH]
		Digital Health [MeSH]		
		Software [MeSH]		
		Technolog*		
		Virtual		
		Remote		
		Assistive		
		Smartphone		
		Comput*		
		mHealth		
		m health		
		Smart-*		
		Self-help		
		Robot*		
		Automat*		
		App		
		Mobile device		
		Digit*		
		Sensor		
		Tablet		
		Portable		
		Interface		
		Virtual reality		
		VR		

* Denotes a wild-card in the search-term.

### 2.2 Inclusion and exclusion criteria

Broad inclusion criteria were set, such that any primary peer-reviewed paper, which evaluated the deployment of a DHT or prototype DHT, to target any aspect of ADHD, in any setting, with adults, was included. By keeping the criteria broad, we sought to include as many examples of DHT development and deployment as possible. We set no limit on the publication date and conducted our last round of searches to identify examples up to and including December 2025. We excluded abstracts, student theses, datasets, theoretical contributions, and technologies deployed solely to participants between 0 and 17 Years Old. Texts were limited to those available in the English language.

In the context of this review, we adopt the definition of a DHT provided by NICE (40). The definition includes technologies, apps, platforms, or software that is intended to benefit an individual's health, or the wider health and social care system. Importantly, it also excludes technologies designed solely for healthcare professional training, or technologies that are only used to facilitate data collection in research studies (44). As such, technologies which were deployed with the intent of researching ADHD neurophysiology only, without an explicit health benefit, are not included in the review.

### 2.3 Screening and study selection

All searches were exported and collated into EndNote 21 (45). Full results were uploaded to the online review management software rayyan.ai (46) and screened for duplication. Two of the authors (FS, SW) then independently screened the titles and abstracts against the inclusion and exclusion criteria to determine eligibility. Where decisions could not be made from the title and abstract alone, the full text was assessed. Disagreements between the two reviewers were settled through regular discussions following the initial round of screening. Inter-rater reliability was 97.25%. Papers which were screened in were assessed by the primary author, with any exclusions made at this stage noted with reasoning. The citation lists of full-text included papers were examined to identify any further eligible examples.

### 2.4 Data charting

Key study information including title, abstract, year of publication, primary/secondary outcome(s), study size, and age of participants was extracted from each paper and recorded in a spreadsheet using Microsoft Excel (47).

To chart the collected papers, we adopt the framework proposed by NICE for DHTs (40), wherein technologies are classified by their intended purpose and stratified into tiers based on their overall risk. Key descriptions are summarized in Table 2 [for further detail, see (40, 44)]. Alongside this, each paper was inductively mapped into a technology cluster, depending on the type of technology primarily being used. This was achieved by initially labelling the core technology as described by the authors. These labels were then iteratively developed into overarching categories based on their similarity to other examples.

**Table 2:** Descriptions of NICE DHT classification tiers [adapted from (40)].

Group	Description
A: System Service	DHTs intended to release costs or staff time, or improved efficiency. Unlikely to have direct health outcomes measurable for individual service users
B: Communicating About Health and Care	Communicating with health and care professionals, or others, to help service users manage their health and care. Allows 2-way communication
B: Health and Care Diaries	Health and care diaries to help service users to manage their own health and wellness. Allows service users to record information to create health diaries. Information and data stay with the service user and are not automatically shared with others for review
B: Promoting good health	Population-level information to help people and service users maintain healthy lifestyles and manage conditions. Provides non-personalized information and resources to service users. May encourage behaviors that promote good health and address issues such as smoking, eating, and exercise. May also provide information about specific conditions
C: Inform Clinical Management	DHTs that record and calculate data and transmit the data to a professional carer or third-party organization, to inform clinical management decisions in the future. Also, DHTs that provide personalized information or guidance to end users to promote healthy living. Information provided by the DHT will not trigger an immediate or near-term action by clinical or care staff
C: Drive Clinical Management	Information provided by the DHT will be used to aid in treatment, aid in diagnoses, to triage or identify early signs of a disease or condition, or will be used to guide next diagnostics or next treatment interventions
C: Diagnose a Condition	Information provided by the DHT will be used to take an immediate or near-term action to diagnose, screen, or detect a disease or condition
C: Treat a Specific Condition	Information provided by the DHT will be used to take an immediate or near-term action to treat, prevent, or mitigate by means of providing a therapy to a human body

Within the NICE DHT framework, the categories *Drive Clinical Management* and *Diagnose a Specific Condition* overlap conceptually. DHTs classified as *Drive Clinical Management* may inform clinical assessment, whilst *Diagnose a Specific Condition* describes DHTs that result in an immediate or near-term action to diagnose, screen, or detect a condition. To support the distinction between the two categories, we classified each DHT on the basis that it would be implemented clinically as described by the authors. DHTs that provided a stand-alone diagnostic or screening outcome, with no integration into traditional assessment pathways, were considered *Diagnose a Specific Condition*. DHTs that supplemented traditional clinical assessment to produce a probability of diagnosis, or propose a clinical support tool that agglomerates existing clinical data, were classified as *Drive Clinical Management*.

### 2.5 Risk of bias assessment

To support the assessment of study design and quality of evidence, RoB analysis was undertaken using the NIH Study Quality Assessment Tools (48). A RoB assessment is generally not performed in a scoping review unless specifically required to support the study aims (42). However, one objective of this review is to examine the quality of evidence for how DHTs would bring about measurable health benefits. As such, a RoB analysis provided the framework for an objective appraisal of the evidence. The NIH tools provide a suite of tailored checklists for different study designs including controlled interventions (CIV), systematic reviews and meta-analyses, observational cohort/cross-sectional (OCS), case-control (CCO), pre-post with no control group (PPNC), and case series (CAS) studies. Checklists range from 9 items for CAS studies to 14 items for CIV studies, with possible responses to each criterion including Yes, No, Cannot Determine (CD), Not Reported (NR), or Not Applicable (NA). The NIH tools were chosen due to their ability to accommodate multiple study designs within a single framework, which aligns with the heterogeneous nature of study designs collated in a scoping review. The NIH tools can additionally be used to provide an overall rating of papers as either Good, Fair, or Bad. However, given that they are not validated tools, and not considered as robust as study-design specific tools (49–51) we do not categorize studies according to quality. We instead report on the descriptive trends observed across the criterion for the collection of papers. The use of more extensive tools was considered out of scope for the purposes of this study, given that RoB is not typically performed in scoping reviews.

### 2.6 Evidence synthesis

An overview of how each component of the review methodology supports our objectives is presented in Table 3. To support with the synthesis of results, we present our findings following the NICE DHT Framework for classification. As such, the results section is structured such that the answers to our research question, namely the papers identified, common outcome measures, types of technology deployed, study design, and sources of bias, are presented for each NICE DHT category. This enables us to identify trends and insights that were specific to each aspect of the clinical pathway. Full tables containing the information extracted for each paper are available in the Supplementary Material.

**Table 3:** Mapping of research objectives to the supporting methodologies undertaken in this review.

Research objective	Supporting methodologies
Conduct a comprehensive search to identify DHTs developed to specifically target adults with ADHD	Development and deployment of search terms across multidisciplinary databases. Selection of relevant examples through set inclusion and exclusion criteria
2. Chart identified DHTs by (a) classifying their intended purpose and (b) identifying core technological features	Use of the NICE DHT Framework for DHT Classification to identify intended purpose. Inductively mapping each example into a core technology grouping
3. Examine methodological trends in the development and evaluation of DHTs	Identify and report on study designs and outcome measures that authors deploy
4. Examine the quality of evidence for these DHTs through a Risk of Bias analysis	Risk of Bias Analysis using the NIH Study Quality Assessment Tools. Identifying common sources of bias through reporting on descriptive trends

## 3 Results

### 3.1 Search results

**Figure 1:**
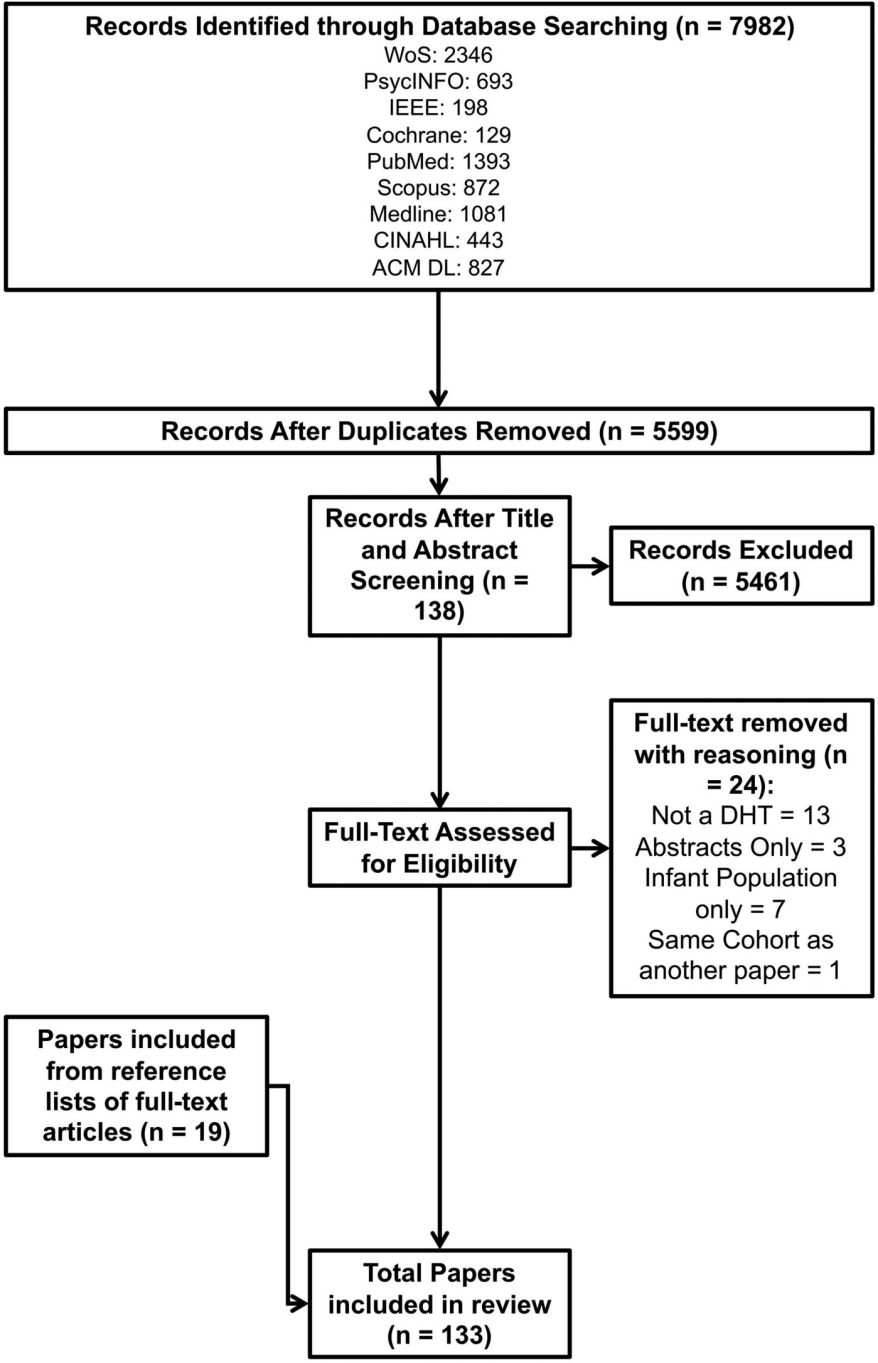
PRISMA-ScR flow diagram of paper selection. The figure shows the flow of papers throughout the screening process, beginning with all searches returned at the top (*n* = 7982) and ending with the final selection of papers (*n* = 133) after deduplication, title and abstract screening, and searching citations of included papers.

Figure 1 shows that after executing the search strategy in each database, 7,982 hits were returned. Following de-duplication, screening for eligibility, and searching through citations of full-text inclusions, 133 papers were included in the review.

### 3.2 Study characteristics and DHT classification

**Figure 2:**
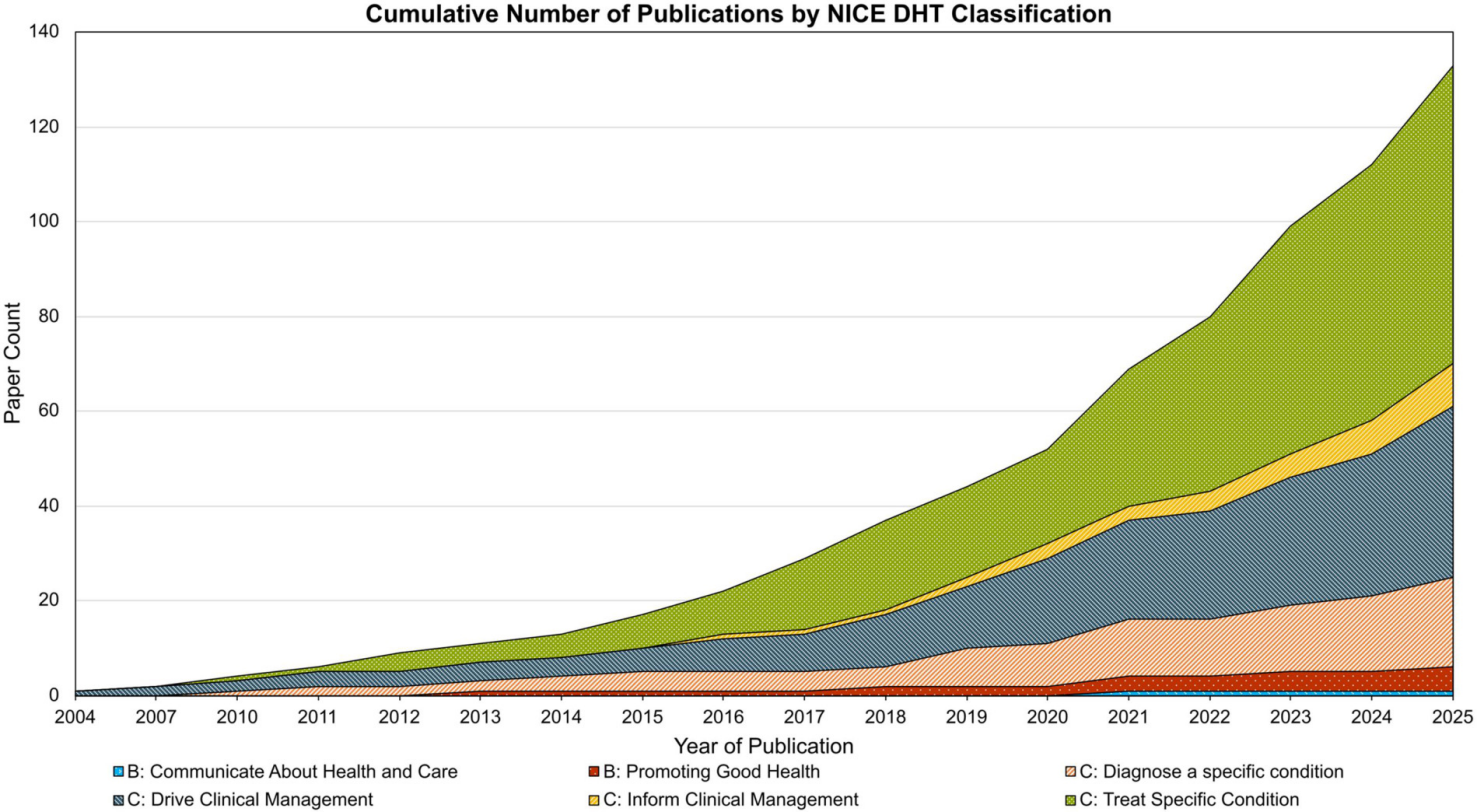
Cumulative count of publications stratified by NICE DHT classification. The figure shows all 133 included publications, stratified by the NICE DHT category they were charted into. Count of publications spans from the earliest paper identified in 2004 up to 2025.

Figure 2 shows the cumulative number of publications for each DHT classification. For class C technologies, 63 (43.37%) were classified as *Treat Specific Condition*, 36 (27.07%) were *Drive Clinical Management*, 19 (14.29%) were *Diagnose a specific condition,* and 9 (6.77%) were *Inform Clinical Management*. For Class B technologies*,* 5 (3.76%) were *Promoting Good Health*, and 1 (0.75%) was *Communicating about Health and Care*.

**Figure 3:**
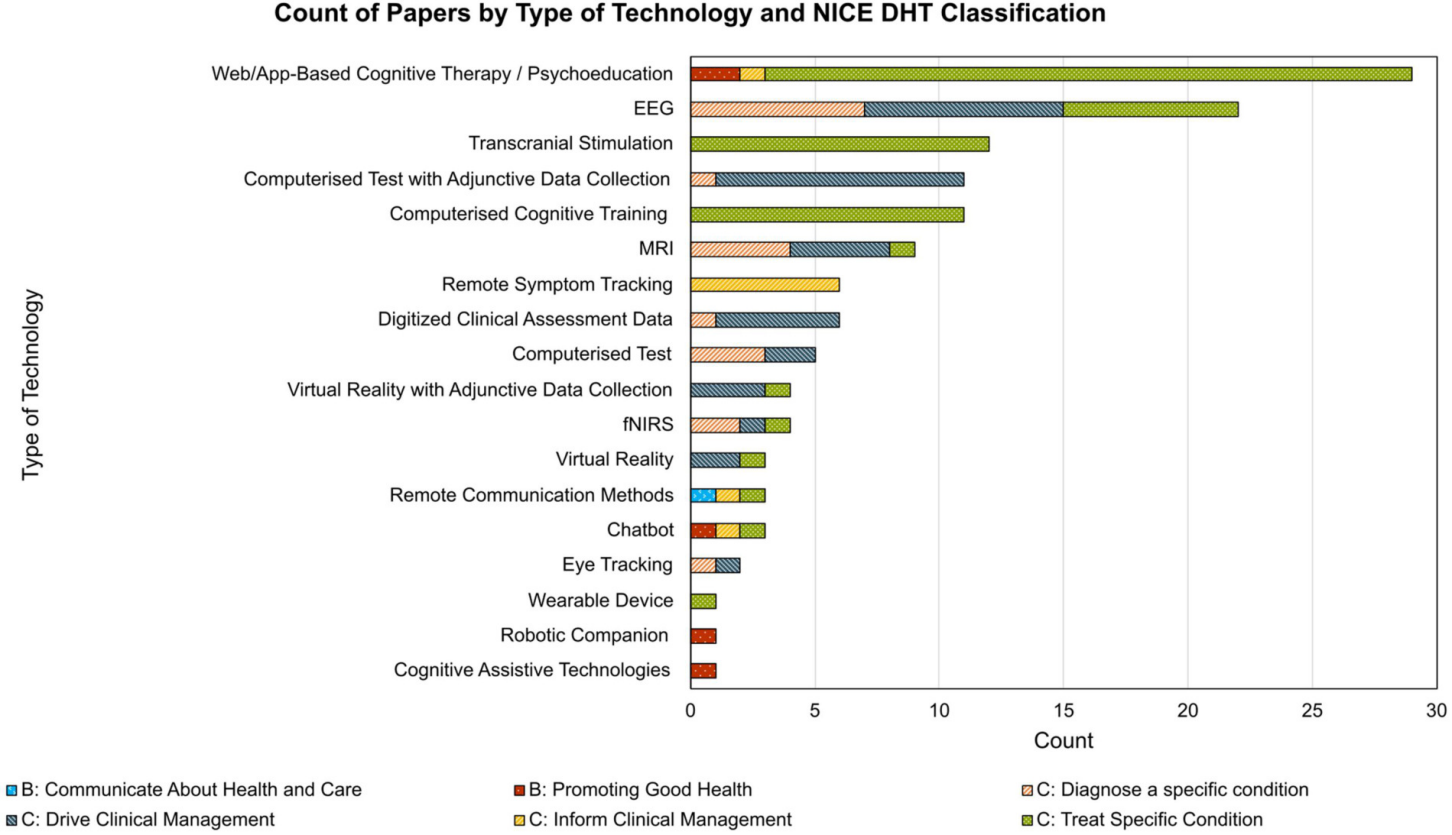
Count of papers by technology type and NICE DHT classification. Each technology type is presented along the *x*-axis, with count along the *y*-axis. Each column is stratified by the NICE DHT classification. EEG, electroencephalography; fNIRS, functional near-infrared spectroscopy; MRI, magnetic resonance imaging.

Alongside charting DHTs into NICE categories, we also categorized papers based on the core technology being deployed, shown in Figure 3. The most used technology was a form of web or app-based cognitive therapy or psychoeducation, used in 29 papers, which were classified predominantly as *Treat Specific Condition* in 26 cases. This was followed by 22 electroencephalography (EEG) based technologies, used in 7 cases to *Treat Specific Condition*, 8 cases to *Drive Clinical Management,* or in 7 cases to *Diagnose a specific condition*. A series of 16 papers examined the use of a computerized continuous performance test (CPT), either in isolation (*n* = 5) or while simultaneously (*n* = 11) collecting other forms of data such as head movement, neuroimaging data, or eye tracking data.

As shown in Table 4, study design varied depending on the NICE DHT classification. For DHTs categorized into *Treat Specific Condition*, CIVs were dominant, used in 38 studies. In contrast, *Drive Clinical Management* and *Diagnose a Specific Condition* were almost all OCS studies, with 34 out of 36 and 18 out of 19, respectively. *Promoting Good Health* and *Inform Clinical Management* were each a mix of study designs. The only paper to be categorized as *Communicate About Health and Care* was a CAS study. Information regarding the average study size, participant age, and % Males for each NICE DHT category is presented in Supplementary Table 1.

**Table 4:** Counts of papers by study design within each NICE DHT Category.

NICE DHT category	Case series	Controlled intervention	Observational cohort/cross-sectional	Pre-Post with no control group	**Total**
Communicate About Health and Care	1	0	0	0	**1**
Promoting Good Health	1	1	1	2	**5**
Diagnose a Specific condition	0	1	18	0	**19**
Drive Clinical Management	1	0	34	1	**36**
Inform Clinical Management	2	2	5	0	**9**
Treat Specific Condition	1	38	7	17	**63**
**Total**	**6**	**42**	**65**	**20**	**133**

Columns present study design types, and rows list NICE DHT Categories. Cell color intensity is proportional to the frequency count in each cell, with darker green shading indicating a higher number of studies and lighter green shading indicating fewer studies for a given combination of DHT category and study design.

Bold denotes the totals for each column/row.

### 3.3 NICE DHT category: treat a specific condition (*n* = 63)

Across this category of papers, several subcategories of intervention shared a common primary technology. 27 out of 63 evaluated the deployment of a form of digital cognitive therapy or psychoeducation (52–78), 13 focused on the evaluation of cognitive training programs (79–91), 9 deployed a form of neurofeedback (NF) (92–100), 9 papers used transcranial direct current stimulation (tDCS) or transcranial alternating current stimulation (tACS) (101–109) and 3 used repetitive transcranial magnetic stimulation (rTMS) (110–112). The remaining 2 papers examined the demographic contributors to successful treatment outcomes for remote telepsychiatry (113) and a haptic feedback wearable to improve symptoms of anxiety and focus (114). Key study-level information for each subcategory is available to view in Supplementary Tables 2–6.

Cognitive-Behavioral DHTs (Supplementary Table 2) typically deployed modules to participants grounded in psychotherapeutic approaches. Most drew on Cognitive Behavioral Therapy (CBT) (55, 58–60, 63, 74, 75), with fewer examples referencing Acceptance and Commitment Therapy (65, 66), Dialectical Behavior Therapy (DBT) (71), Self-determination theory (76), Stress management (77), or more general psychoeducational principles (52, 67, 68). DHTs were most frequently deployed as part of a guided program (52, 54, 57, 58, 64–67, 71–74, 76, 77), with fewer examples of DHTs used as a self-guided tool (53, 55, 56, 59, 60, 63, 69, 70, 75), or as companion apps that accompanied traditional face-to-face therapies (61, 62, 68). 1 study additionally developed and deployed a chatbot to accompany self-guided app content (78). Interventions were most commonly 6 weeks in length (Range 3 weeks—16 weeks). Content was mostly developed to address general ADHD-related challenges in 16 cases (53, 55, 56, 58–60, 63, 64, 67–69, 71–74, 78). Fewer interventions focused on specific aspects such as emotion regulation (61, 62), executive functioning (54, 75), and comorbidities including cannabis use disorder (52) and anxiety and depression (70). Effectiveness was largely assessed using self-report measures of ADHD symptoms including the Adult ADHD Self-Report Scale (ASRS) (55, 57, 58, 61, 75), the ADHD Rating Scale (ADHD-RS) (74, 76) and DSM-IV Current Symptoms Scale Self-Report (CSS) (63). Only one paper reported observer-rated symptom severity through the Integrated Diagnosis of ADHD—Revised (IDA-R) framework (68). A majority of papers (53, 54, 56, 59–62, 64, 65, 67, 69–71, 73, 76) reported primary outcomes related to user experience, including feasibility, acceptability, adherence, and user satisfaction. Together, these results indicate a tendency to deliver DHTs as part of a guided program, yet the high proportion of outcomes based around feasibility and acceptability suggests many remain in an early stage of development.

Another 13 papers evaluated cognitive training programs (Supplementary Table 3). These DHTs present the user with increasingly challenging tasks designed to target and strengthen neuropsychological processes implicated in ADHD. 11 programs ran on computer or mobile interfaces (80–83, 85–91), whilst 2 were based on VR paradigms (79, 84). Training most often targeted working memory (79, 81–83, 85–87, 89, 90), with fewer examples targeting attention (80, 84, 88) or executive functioning more generally (86, 87, 91). Outcomes were split into “near-transfer” measures which relate directly to the neuropsychological process being targeted, and “far-transfer” measures which examine whether improvements transfer to a generalized setting. Near-transfer measures were more frequently deployed, in 10 studies, and consisted of tests of cognitive ability such as CPTs (80, 84, 86–88, 91), the Wechsler Adult Intelligence Scale (WAIS) IV Digit Span (82, 83, 89) or Wechsler Memory Scale (WMS) IV Spatial Span (82, 83, 85). Far-transfer measures appeared in fewer cases (*n* = 8 studies) and relied largely on self-report measures such as the ASRS (82, 83, 85–89), and both the Cognitive Failures Questionnaire (CFQ) and Barkley Deficits in Executive Functioning (BDEF) (82, 83, 85).

A further 9 papers evaluated the deployment of neurofeedback (NF) protocols (Supplementary Table 4). NF operationalizes a neuroimaging modality to produce visual or acoustic stimuli that are fed back to the participant to serve as a basis for training to alter brainwave activity. Various parameters have been identified as targets for ADHD, and in this collection of papers, the theta-beta ratio was most commonly used in 4 studies (92, 97–99), with other examples including sensorimotor rhythm (92, 93, 98), alpha power (99), dorsal anterior cingulate cortex (dACC) activation levels (100), prefrontal HbO2 Concentration (94), and slow cortical potentials (95). Total amount of time spent actively performing NF varied from 8 h to 40 h, with an average across the papers of 18.08 h. Outcome measures again typically consisted of CPTs, used in 6 papers (92, 96–100), self-reports of ADHD symptoms in 6 papers (92, 95, 97–100), and in 3 papers (95, 97, 99), self-reports of symptoms of depression.

A total of 12 studies examined forms of non-invasive brain stimulation (Supplementary Table 5) including transcranial direct current stimulation (tDCS, *n* = 8), transcranial alternate current stimulation, (tACS, *n* = 1), or repetitive transcranial magnetic stimulation (rTMS, *n* = 3). These modalities either apply weak electrical currents to alter excitability (tDCS), alternating electrical currents to stimulate rhythmic electrophysiological activity (tACS), or magnetic pulses (rTMS) to modulate cortical excitability in specific regions. The dorsolateral prefrontal cortex (dlPFC) was the most common region targeted in 7 papers (101, 102, 104, 106, 107, 109, 111), with other areas including regions involved in P300 waveform generation (103), posterior brain regions (105), bilateral prefrontal regions (110, 112), and frontoparietal regions (108). Total amount of time receiving stimulation averaged 5.88 h (Range 0.33–14 h). Consistent with other subcategories of DHTs within *Treat a Specific Condition*, the most common outcome measures were a combination of ADHD symptom severity self-reports including the ASRS (102, 106, 107) and the Conners Adult ADHD Rating Scale (CAARS) (110, 112) alongside a form of CPT in 2 papers (101, 105).

Altogether, these studies showed a consistent focus on targeting ADHD-related symptoms, whilst relying on reporting efficacy through self-reported symptom measures. Alongside this, feasibility and acceptability were frequently reported, particularly for cognitive-behavioral DHTs. Cognitive training, neurofeedback, and non-invasive brain stimulation DHTs further emphasized near-transfer cognitive measures, and overall, comparatively fewer studies incorporated observer-rated outcomes or evidence of broader functional transfer.

### 3.4 NICE DHT category: drive clinical management (*n* = 36)

Within this category (Supplementary Table 7), 34 (out of 36) deployed a DHT to collect data used to support a diagnosis of ADHD through machine-learning or logistic regression-based classifier approaches. Approaches were split between using technologies to create unimodal (*n* = 15 papers) or multimodal datasets (*n* = 19 papers). Unimodal datasets relied on single sources of data such as EEG in (115–121), digitized standard clinical assessment data (122–124), MRI (125–127), VR (128, 129), CPTs (130, 131), eye tracking (132) and fNIRS (133). Conversely, multimodal datasets typically combined physiological and behavioral measures. The most commonly deployed was the QbTest + (134–139), which combines CPT metrics with head movement measurements, whilst other examples include actigraphy and heart rate data with CPT metrics (140), eye-tracking with CPT metrics (141, 142), VR-based CPT with multiple additional forms of data (head movement, eye tracking, EEG, subjective experience, and fNIRS) (143–145), multiple formats of standard clinical assessment data (self-report data with interview transcripts) (146, 147), and EEG with measures from the Wender-Utah Rating Scale (WURS) (148). Outside of diagnostic assessment, 1 paper examined the use of the QbTest + to evaluate medication response (149), and 1 used MRI in a classification study to discriminate between ADHD subtypes (150).

Classifier performance was evaluated in 30 studies, with the majority reporting accuracy (percentage correctly classified over the total dataset) (115–122, 125–127, 133, 137, 138, 140, 145–148), sensitivity (proportion of true positives over true positives and false negatives) and specificity (proportion of true negatives over true negatives and false positives) (115, 117–120, 123, 125–127, 134–139, 142, 145, 147). Area under curve (AUC) values were reported in 16 papers (115, 120–122, 124–126, 130–132, 138–142, 146), with raw receiver operating characteristics (ROC) presented in 9 papers (115, 120, 122, 124–126, 132, 138, 139). 28 studies derived classifier performance by comparing metrics from an ADHD group against a control group, with Healthy Controls used in 27 studies. 2 studies additionally included participants with Schizophrenia (115) and a range of psychiatric disorders (124), whilst another sought to classify participants into those diagnosed with ADHD or with Autism Spectrum Disorder (138).

Together, these studies generally followed a similar approach wherein DHTs are deployed to augment established diagnostic procedures. This was achieved by leveraging behavioral indices, neurophysiological measures, or both, to support classifier-based discrimination between ADHD and comparison groups.

### 3.5 NICE DHT category: diagnose a specific condition (*n* = 19)

A further 19 papers also proposed a DHT specifically for diagnostic or screening purposes (Supplementary Table 8). In this group, 17 proposed classifications based on unimodal data, most commonly through EEG in 7 (151–157), MRI in 3 (158–160), CPTs in 3 (161–163), fNIRS in 2 (164, 165), and eye tracking (166) or through psychometric data (167) in 1 each. Fewer papers combined data in multimodal approaches, with 1 combining fNIRS with eye-tracking and CPT data (168), and 1 combining MRI with neuropsychological self-report measures (169).

Accuracy was again the most reported outcome measure in 12 cases (151, 152, 154, 155, 157–160, 164, 166, 167, 169), with 8 further specifying the sensitivity and specificity (151, 153, 154, 157, 159, 160, 162, 168), 7 including an AUC metric (151, 153, 156, 162, 163, 165, 168), and 4 showing the raw ROC (151, 163, 165, 168). All papers included HCs as a comparator group, with 1 study additionally instructing a group to “feign” ADHD to investigate a screening technology that would be sensitive to malingering (162).

Overall, these papers all use similar classification approaches as to those within *Drive Clinical Management*, and are evaluated in a similar manner. However, these examples differ in that the DHT is not situated as an aid to diagnosis, or adjunct to traditional assessment pathways. Instead, they propose that the DHTs would provide an automated output to facilitate immediate or near-term diagnosis.

### 3.6 NICE DHT category: inform clinical management (*n* = 9)

A total of 9 papers were classified as *Inform Clinical Management* (Supplementary Table 9). DHTs in this selection are defined by their purpose of gathering information that is transferred to a third-party to inform clinical decision-making. 5 papers (170–174) proposed the use of tracking tools for longitudinal monitoring of ADHD symptoms, 2 used either SMS (175) or an app (176) to track and improve medication adherence, 1 proposed a chatbot interface for self-screening (177), and 1 implemented a remote smoking monitoring system (178).

Two of the symptom-tracking technologies were early stage DHTs evaluated qualitatively with Backer et al. (170), and Patrickson et al. (171), both recruiting clinicians and end-users to iteratively develop and test app content. Conversely, Surman et al. (172), Sankesara et al. (173), and Ware et al. (174), all pursued a similar approach, wherein they deploy their technologies to collect real-world data for longitudinal symptom monitoring. Outside of symptom monitoring, both Biederman et al. (175), and Carvalho et al. (176), evaluated the effectiveness of their DHTs by examining medication possession rates, whilst the smoking monitoring (178) and chatbot tools (177) were deployed to small pilot cohorts with primary outcomes relating to feasibility.

### 3.7 NICE DHT category: promoting good health (*n* = 5)

A further 5 papers were categorized as *Promoting Good Health* (Supplementary Table 10). Of these, 3 evaluated a form of mobile app that provided resources for concerted information provision (179–181). These examples differed from the psychoeducational content of DHTs classified in *Treat Specific Condition* given that they focused on improving patients' knowledge of ADHD through non-personalized advice, rather than directly addressing ADHD symptoms. Seery et al. (179), evaluate co-produced psychoeducational content presented through graphical and text interfaces, whilst Luiu et al. (180), investigated user preferences for a prospective app focused on providing psychoeducation for specific dimensions of ADHD centered around the Precaution Adoption Process Model (PAPM) of behavior change. Jang et al. (181), evaluated a form of interactive content delivery through a chatbot, Todaki, that was designed to support the user in learning self-help skills to manage their condition. Outside of psychoeducational DHTs, Lindstedt et al. (182), report on the implementation of a suite of cognitive assistive technologies that were deployed to support daily activities, and Store et al. (183), evaluate the effectiveness of a soft-robotic sleep companion for improving sleep quality.

All studies incorporate investigation into usability and perceived satisfaction or helpfulness, while Lindstedt et al. (182), further examined the impact of assistive technologies on Quality of Life and Store et al. (183), report on changes in sleep measures derived from the Insomnia Severity Index and actigraphy data.

### 3.8 NICE DHT category: communicate about health and care (*n* = 1)

One paper was classified as an evaluation of a DHT for *Communicate about Health and Care* (Supplementary Table 11). In this paper Adamou et al. (184), surveyed 117 end-users on their experiences of remote diagnostic assessment that was introduced into the specialist adult ADHD and Autism Service as part of the COVID-19 pandemic. They adapted the Telehealth Usability Questionnaire to investigate whether users found remote telecommunication during assessment useful and satisfactory. They identify possible differences in preference across gender, with females more willing to continue conducting remote assessment. They conclude that for ADHD, remote assessments may be feasible given the high levels of reported satisfaction. However, they note in their study, future work should focus on understanding user satisfaction once the patient has progressed further down the clinical pathway. This limitation was due to their study being cross-sectional in design and only surveying participants prior to them receiving their diagnostic outcomes.

### 3.9 Risk of bias analysis

For analyzing trends in RoB, we present findings from all study designs for *Treat Specific Condition*, whilst focusing on OCS studies for *Drive Clinical Management* and *Diagnose a Specific Condition*. We place less emphasis on describing trends in bias for *Promoting Good Health*, *Inform Clinical Management*, and *Communicate about Health and Care*, given the lower number of examples within each study design. All RoB data is available to review in Supplementary Tables 12–28.

**Figure 4:**
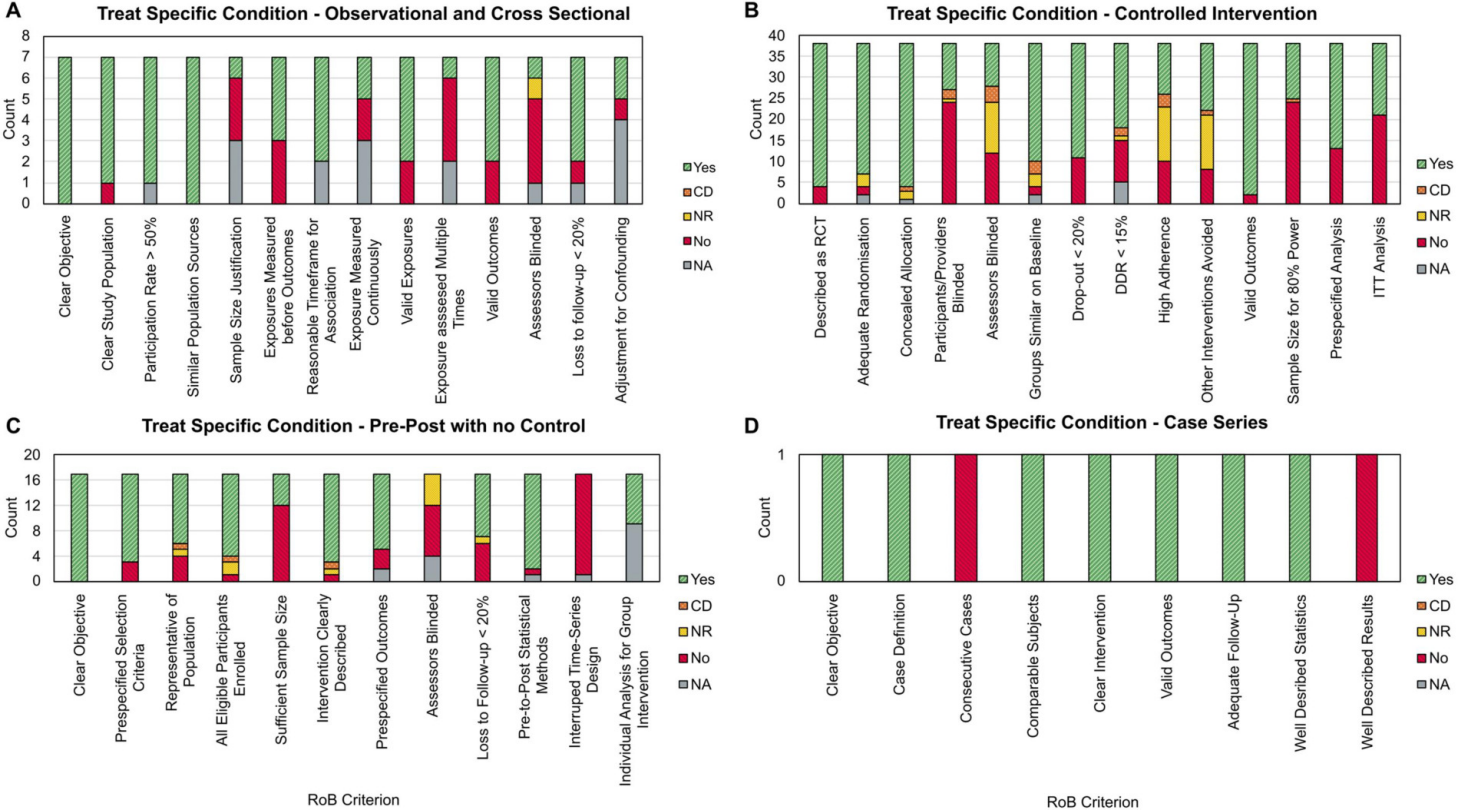
Four panels of stacked bar charts showing risk of bias (RoB) criterion scores for each paper type in the NICE DHT category treat specific condition. **(A)** Observational and cross-sectional, **(B)** controlled intervention, **(C)** pre-post with no control, **(D)** case series. Each criterion from the RoB Checklist is presented as a column, with the *y*-axis showing total counts. Each bar is stratified to show the count of each possible response. CD, cannot determine; NR, not reported; NA, not applicable; DDR, differential dropout rate; ITT, intention to treat.

Across the 63 papers classified as *Treat a Specific Condition*, our RoB analysis identified several consistent sources of bias. As shown in Figure 4, a lack of participant and intervention provider blinding was most prevalent, either not being implemented or reported in 25 (65.79%) CIV studies. Outcome assessor blinding was similarly uncommon (given the reliance on self-reported outcome measures) with a further 24 (63.15%) CIV, 5 OCS (71.42%) and 13 PPNC (76.47%) papers not including or reporting on using blinded outcome assessors. With regards to sample size, 24 CIV (63.15%) papers did not include power analyses to demonstrate at least 80% power, whilst 12 PPNC (70.58%) papers did not include sufficient justification or analysis of their sample size. For study adherence, 10 CIV (26.32%) papers reported a low level of adherence to the intervention, whilst a further 13 (34.21%) CIV papers did not report on investigations into adherence and 10 PPNC (58.82%) studies had a loss to follow up greater than 20%. Taken together, the high levels of non-blinding may undermine the internal validity of causal claims, whilst the lack of power analyses and investigation into adherence introduces uncertainty whether the observed effects represent true therapeutic benefit that translates into real-world scenarios.

**Figure 5:**
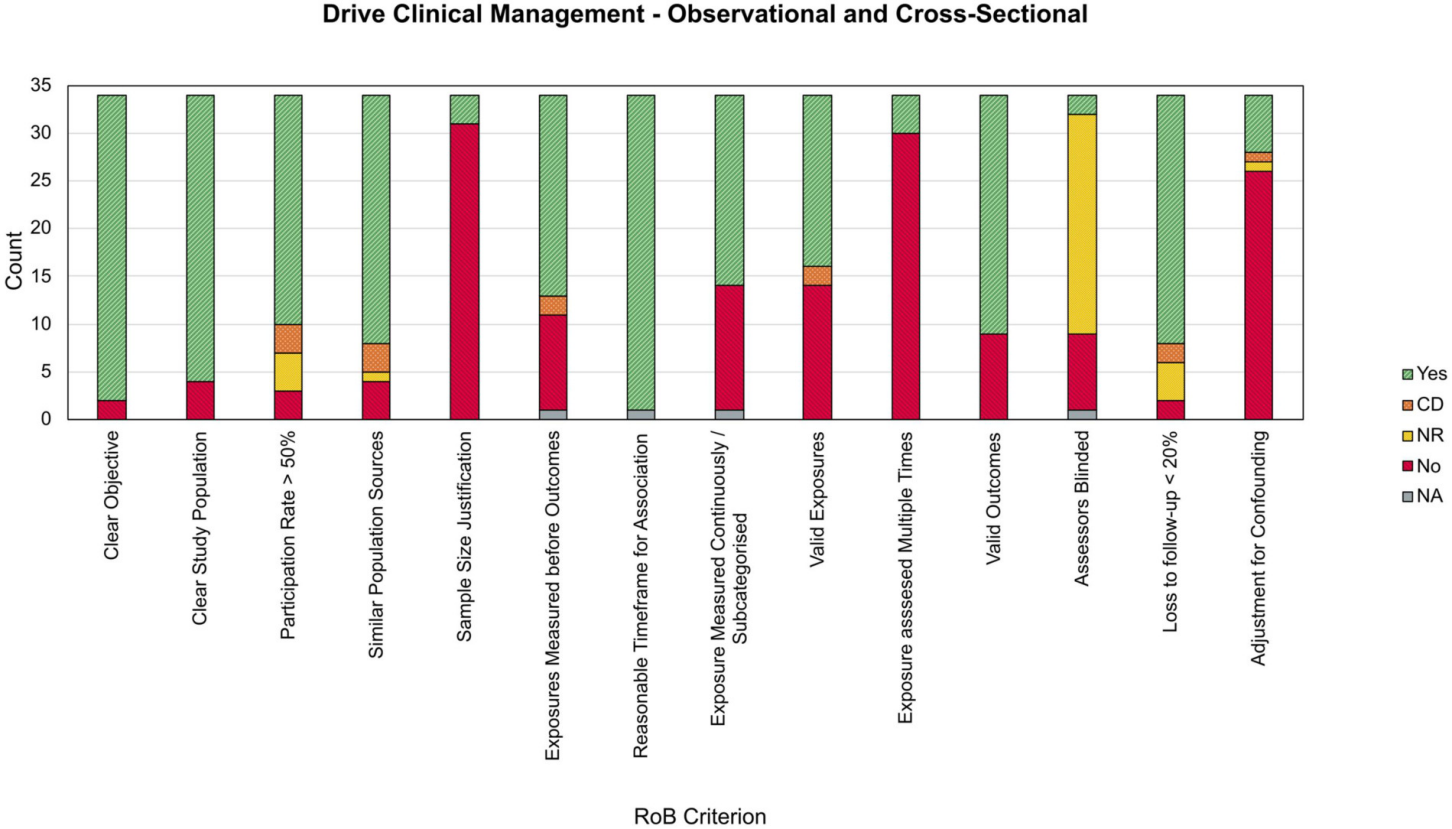
Stacked bar chart showing RoB criterion scores for observational and cross-sectional studies within drive clinical management. CD, cannot determine; NR, not reported; NA, not applicable.

In contrast to *Treat a Specific Condition*, DHTs which were classified as *Drive Clinical Management* were predominantly observational in design, with 34 OCS studies and 1 PPNC and 1 CAS study. Within the OCS group, shown in Figure 5, sample size was again a consistent source of bias, with only 3 papers (8.82%) including a justification of sample size or description of power (130, 132, 136). In addition, only 6 studies (17.65%) included adjustment for key confounding variables (124, 128, 130, 132, 134, 135), and 4 (11.76%) included repeated measures of the exposure (i.e., use of the DHT) (120, 126, 136, 139). These DHTs leverage an association between participant features and their ADHD diagnosis, yet these sources of bias may undermine the validity of any association by either not investigating the statistical power of these associations, other explanatory confounding variables, or any temporal instability in the features used for classification. Conversely, the collection's strengths were in specifying the research objectives, study population, and population sources.

**Figure 6:**
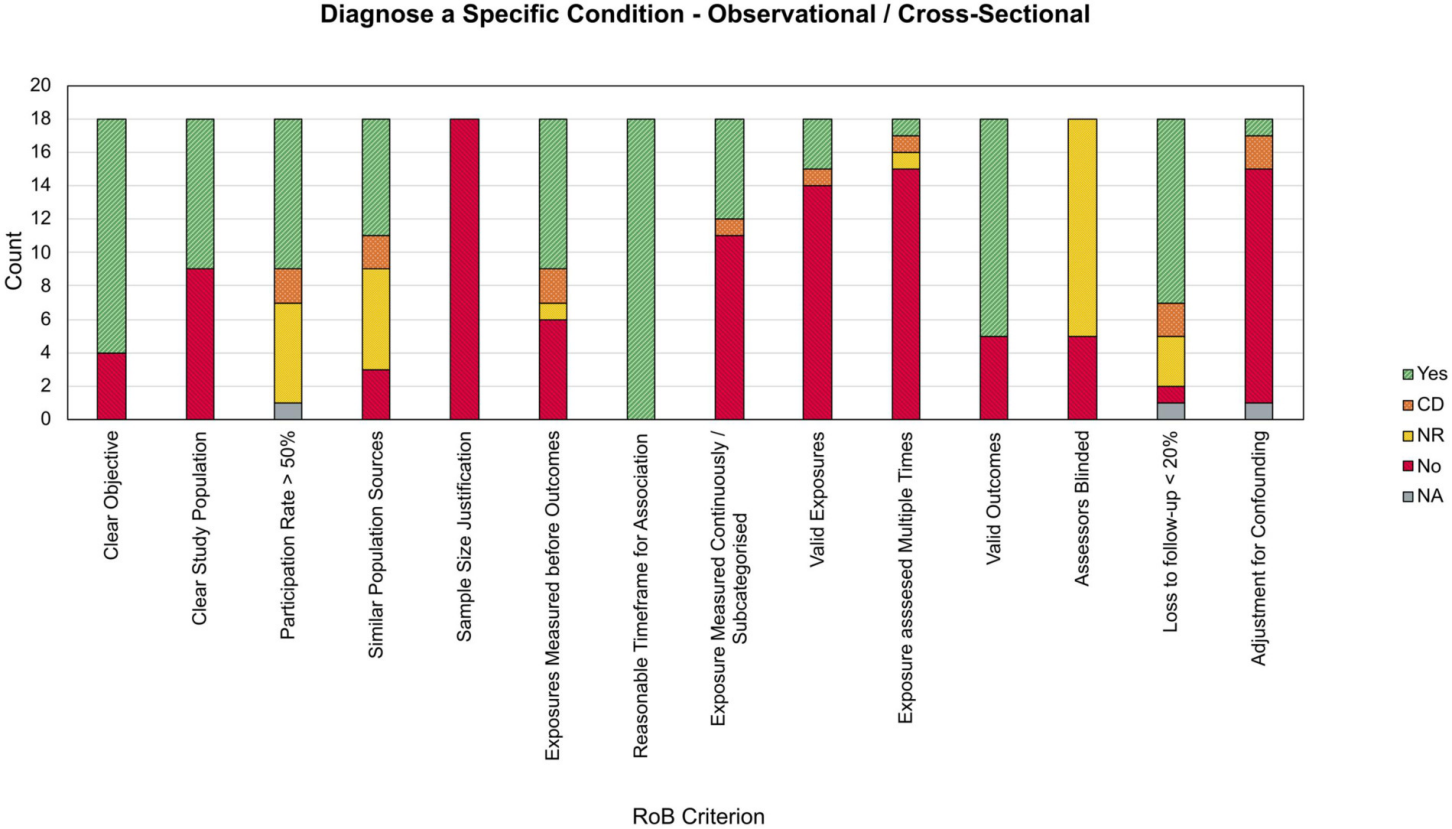
Stacked bar chart showing RoB criterion scores for observational and cross-sectional studies within diagnose a specific condition. CD, cannot determine; NR, not reported; NA, not applicable.

Papers within *Diagnose a Specific Condition* were similarly observational by design, with 18 papers classified as OCS studies and 1 as a CIV study. As with *Drive Clinical Management*, the most common sources of bias across the OCS papers, shown in Figure 6, included a lack of sample size justification in all 18 papers, a lack of adjustment for confounding in 14 (77.78%) papers, and not measuring the exposure more than once in 15 (83.33%) papers. In comparison to *Drive Clinical Management*, a greater proportion of papers were less clear on the population sources and inclusion criteria. Various examples did not describe how an ADHD diagnosis was established in participants in sufficient detail [e.g., (151, 158–160, 162, 166)] or being defined through cut-offs on screening measures (167) in comparison to a full, valid, diagnosis.

The remaining papers classified as *Inform Clinical Management* and *Promoting Good Health* were each a mix of study designs, whilst the 1 paper classified as *Communicate about Health and Care* was a CAS study. Common sources of bias were similar to the preceding categories, with a lack of assessor blinding and power analysis most prevalent. Given the low number of papers in each category, caution should be applied in interpreting trends in bias for these categories. However, key areas of bias appear consistent across NICE categories wherein the technology is premised on deployment to and use by an ADHD patient as an end-user.

## 4 Discussion

### 4.1 Summary

In this review, we identified 133 papers, and using the NICE classification framework, charted DHTs to identify a concentration of technologies that predominantly aim to treat a specific condition or support/automate diagnosis for adults with ADHD. Our findings present a high proportion of papers focusing on usability, feasibility, and reporting on early-stage trial results. Through our quality assessment, we identified gaps in blinding, adherence, and deployment of DHTs longitudinally. Here, we highlight key priorities for the next wave of research, which should further consider how to generate suitable evidence that supports DHT implementation as valid clinical tools.

### 4.2 Main findings

#### 4.2.1 DHTs for direct health outcomes

Various DHTs sought to directly improve health outcomes by targeting symptoms specifically implicated in ADHD. With medication discontinuation rate in adults with ADHD higher than expected (185), DHTs may work as scalable nonpharmacological tools that provide treatment. NF, tDCS, tACS, rTMS, and cognitive training programs included in this study shared a rationale of providing a structured, non-invasive treatment program, with some examples of at-home deployment (83, 85, 99, 106, 107, 109), furthering their potential as treatment options.

Another large cluster of papers evaluated a form of mobile or web-based cognitive therapy or psychoeducation. Treatment guidelines for ADHD recommend offering psychological treatment for medicated patients whose symptoms still cause impairment or when patients have made an informed choice to not take medication (15). Reviews have highlighted that there is a higher proportion of improved long-term outcomes when pharmacological and non-pharmacological treatment is combined (186), and previous qualitative investigations have highlighted the opportunity to implement DHIs as an adjunct to usual care (187).

Most DHTs for direct health outcomes included in this review were still early-phase or pilot in nature. Below, we present explicit recommendations based on our analysis which may support researchers in further considering how these DHIs may be developed and integrated alongside existing service provision.

##### 4.2.1.1 Sufficient blinding is essential to demonstrate efficacy

Previous reviews in pediatric ADHD (188) have shown that estimates of efficacy for non-pharmacological treatments are diminished when only considering studies with adequately blinded raters or raters not proximal to the therapeutic setting. These same concerns were identified through our RoB analysis, as most studies failed to implement participant and assessor blinding. Furthermore, several papers report being unable to find significant between-group differences (80, 97, 105, 112) when implementing an active control, or attribute their significance to placebo when only using wait-list controls (92). Future studies may wish systematically examine the efficacy of these interventions, with respect to blinding and assessor proximity, to align with previous reviews which have examined the same intervention types in pediatric ADHD populations (188, 189).

##### 4.2.1.2 Adherence assessment and engaging participants promotes DHT success

Another key concern in this category we identify is a low level or lack of reporting on adherence, and high dropout rates. This was most clearly exemplified in Marcelle et al. (81), who did not proceed with their analysis due to having only a 38% completion rate for their cognitive training program. They qualitatively investigated reasons for dropout to find a high level of participant displeasure with the training and difficulty remaining engaged. In practice, an intervention with this level of adherence is at risk of not being adopted, and the authors' findings highlight the importance of designing, and rigorously investigating, a DHT to ensure it remains feasible, acceptable, and usable once deployed. As argued by Spiel et al. (190), actively involving participants with ADHD as partners in the design process will more adequately identify whether a technology is sufficiently designed to accommodate their experiences and preferences.

#### 4.2.2 DHTs for diagnosis

53 out of the 133 papers we identified deployed a form of DHT which aimed to either support or automate a diagnosis of ADHD in adulthood. Currently, wait-times can exceed 2 years in the UK and trials have shown diagnoses can take an average of 2.75 h of clinician time (191). Previous reviews indicate a significant individual and societal cost associated with undiagnosed ADHD (192), with experts by experience highlighting the emotional toll associated with a lack of clinical recognition (193). By developing diagnostic DHTs, there is an opportunity to improve the process and enable patients to receive access to suitable and effective care. Licensing of the QbTest + by NICE (194) and the NEBA system by the FDA (195), shows that technologies are beginning to be implemented clinically to support ADHD diagnosis. However, both licensed products are currently restricted to children up to 17 years, indicating a need to produce suitable evidence for their utility in adult populations.

##### 4.2.2.1 Health system context is a vital component of DHT design

Our review separated diagnostic DHTs into two categories, either *Drive Clinical Management* or *Diagnose a Specific Condition*, depending on the reported degree of implementation alongside traditional clinical assessment pathways. Various technologies clearly complemented traditional assessments, such as classifiers which used transcripts and audio features from diagnostic interviews (122, 146), or others like the QbTest + (134–139) which specify their function explicitly as support tools. However, other DHTs classified in *Diagnose a Specific Condition* did not specify whether their DHT would function as either an adjunct or a direct replacement for assessments, instead focusing on reporting model performance. Whilst the NICE DHT Framework is most suitable for evaluating technologies that are likely to be commissioned in a healthcare service, our use of this framework to chart examples from a basic research environment has elucidated a key consideration for future studies. Namely, there are significant questions which remain over *how* these tools should be used, and by whom, within a diagnostic framework. Previous investigations into clinicians' perspectives highlight the need to suitably place DHTs within their workflow (196). Technologies should ideally streamline, rather than bloat, a clinical process, and future studies which address these questions will help to remove the barriers that prevent translation into clinical practice.

##### 4.2.2.2 Measure performance across multiple timepoints in varied cohorts

Our RoB analysis identified that almost all studies implemented diagnostic DHTs cross-sectionally with measures of the exposure (i.e., use of the DHT) only collected once, using restrictive cohorts that lacked external validity. The impacts of only evaluating performance in one session are highlighted clearly in Muller et al., (120), wherein they evaluate an EEG-based classification model using data collected from the same participants 12 months and 24 months after the original study. Comparing the model performance over the 3 timepoints, they report an Intraclass Correlation Coefficient (ICC) of 0.623, indicating variable performance that can result in a change of classification for participants. A key facet of the current diagnostic framework for psychiatric disorders is the need to demonstrate strong test-retest reliability (197), and future studies should consider deploying these technologies longitudinally to further investigate this characteristic.

Furthermore, our RoB analysis highlighted a consistent lack of sample size justification. As with group-level statistics, sample size has been shown to be a key determinant of machine-learning based classification performance in neuropsychiatric contexts (198). Larger sample sizes introduce heterogeneity to the dataset that may dampen performance but improve model generalizability. Future studies may benefit from implementing guidance to justify adequate sample sizes for clinical prediction models (199), and adhering to updated reporting guidelines to convey the degree of confidence in their findings (200).

An additional concern for these categories was the finding that studies often did not adjust for confounding variables in their analyses. Several examples in this review acknowledge this as a limitation and account for it by having set rigorous selection criteria that exclude participants with comorbidities or a history of other psychiatric diagnoses (125, 130, 141, 142). However, given the high rate of comorbidity in adults with ADHD and a high rate of misdiagnoses, excluding these participants comes at the cost of reducing external validity.

Comorbidities in general were overlooked in a diagnostic context, given the extent to which classification was premised on differentiation of ADHD participants from healthy controls. In practice, clinical guidelines suggest clinicians rule out manifestations that could be explained by other psychiatric diagnoses, or identify coexisting conditions (15). Without adequately training diagnostic models on datasets that include features associated with other conditions, these DHTs would not align with the current assessment framework.

To address these gaps, studies should aim to demonstrate validity by deploying DHTs longitudinally in larger, more varied cohorts that are reflective of patients who would present to the clinic. Previous reviews have additionally highlighted methodological concerns regarding train-test split protocols which introduce circular analysis that inflates performance metrics and have identified a lack of external validation datasets (201), which should also be addressed in future studies.

#### 4.2.3 Remote monitoring and self-management tools

Outside of direct treatment or diagnosis, DHTs were charted into the *Communicate about Health and Care*, *Promote Good Health*, and *Inform Clinical Management* categories. Mostly, these technologies were either remotely gathering information to support symptom tracking, automating medication reminders, or providing interactive digital resources to promote effective self-management.

##### 4.2.3.1 Stakeholder consensus promotes DHT success

Previous qualitative investigations have shown patients are most positive about the potential for remote monitoring technologies to improve ongoing support and management of ADHD (196). Given the highly variable nature of individual responses to pharmacotherapy and the need to establish greater evidence for their long-term efficacy, implementing a DHT that non-invasively gathers longitudinal data to monitor changes in symptom severity with medication may serve as a useful treatment adjunct. The approach taken by Surman et al. (172), which added elements of personalization by tailoring symptom monitoring to those most relevant to the participant, could promote participant engagement. However, their study showed low adherence, with only 70 participants out of 206 completing the study exit measures, and only 22% replying to all SMS prompts, demonstrating the need to balance the burden of continuous prompting with the value that users receive from the insight gained. At the same time, the clinician has a responsibility to provide regular monitoring and adjustment (15), and future tools could further demonstrate their utility by considering what would be valuable to both clinicians and patients, thereby situating a DHT as a means of building a working alliance. The approaches taken by Patrickson et al. (171), and Backer et al. (170), exemplify this, wherein they build their tools based on multiple stakeholder perspectives to understand what would be valuable to patients and what barriers exist in implementing remote monitoring technologies into clinician's workflows.

##### 4.2.3.2 Self-help technologies should have a clear targeted use-case

Outside of remote monitoring, self-help materials are a scalable means of providing resources to empower patients. As shown by the lower-risk classification of DHTs for “Promoting Good Health” or “General Wellness” in regulatory guidelines (40, 202), there are fewer barriers associated with their development. Nonetheless, they require rigorous evidence for their utility, and previous reviews have highlighted scarce evidence on whether standalone self-management information can reduce symptoms for young people with ADHD (203). Further specifying where self-help materials might be useful as an adjunct, as seen in Seery et al. (179), who focused on providing information specifically around access to healthcare for those awaiting diagnosis, may identify where these resources can be best deployed to complement existing treatments.

Previous investigations in the context of anxiety have shown users tend to acquire and utilize multiple self-help DHTs to build their own tailored collection of tools that support self-management (204). The example of Lindstedt et al. (182), who trialed various digital assistive technologies—that the participants themselves chose to implement—exemplifies this, whilst also adding an often-overlooked occupational perspective. Guidelines suggest that clinicians consider occupational needs when planning treatment (15), and implementing digital tools as a resource chosen in collaboration with patients to support daily functioning may bolster more holistic treatment plans. A recent review into ADHD service provision in the UK (193) has highlighted schemes that diversify the primary care workforce as an area where self-help DHTs could be “prescribed” by multidisciplinary teams to support long-term management.

### 4.3 Identifying research priorities through a framework for DHT development

Throughout this review, we have highlighted a range of gaps within the evidence base and presented suggestions to improve the development and evaluation of DHTs within each of the NICE classifications. In this section, we highlight two key research priorities to strengthen the development and evidence base for DHTs across the NICE classifications.

#### 4.3.1 Research priority 1: situate DHTS for the wider context of ADHD care provision

A unifying trend across all categories in this review was a greater need to extend investigations beyond establishing efficacy in research contexts and understand the wider context in which DHTs would be adopted. The NICE classification system identified a gap in consideration of how diagnostic DHTs would sit within clinical pathways, whilst for treatment-focused DHTs, our analysis showed a need to better evaluate adherence and understand patient experience. Remote monitoring tools additionally require integration with the healthcare system that can be achieved through meaningful stakeholder involvement, whilst self-help tools may be best suited as complements to existing management protocols. The need to consider DHTs from a systems perspective is further highlighted by the lack of examples we identified that were charted into the *System Services* category of the NICE DHT Framework.

Several frameworks exist which can be used to develop a greater understanding of the context in which an intervention would be delivered. For example, the MRC Complex Intervention framework (205) outlines a strategy for combining investigations of feasibility and implementation alongside an evaluation of efficacy. Such a strategy may be appropriate given the numerous factors involved in the management of ADHD in adulthood, such as the level of expertise required to treat and diagnose the condition, the involvement of multiple care settings, and the wide array of functional domains that are affected. This necessitates that solutions be multi-dimensional and can operate across interconnected healthcare domains.

#### 4.3.2 Research priority 2: involving users and user feedback in the design process

Although there was a focus in many papers on testing the acceptability of a DHT, a minority of papers reported a form of development which actively implemented this feedback into an iterative design process. Where feedback was actively implemented, there was a clear benefit to the development of the technology. For example, the “myADHD” intervention first utilized participatory design in Flobak et al. (53), to improve relevance to participants, and then gathered early user feedback through piloting in Nordby et al., (59). Difficulties in adherence were reported, and the intervention was subsequently restructured with deliberate efforts to improve uptake in Nordby et al., (60), before being evaluated for efficacy in Kenter et al., (55). Through a combination of research methods, the authors prioritize involving stakeholders, consider implementation, and then subsequently test for efficacy. The “Understanding and Managing Adult ADHD Programme” similarly combined multidisciplinary research methods with a joint investigation into feasibility and acceptability through qualitative research in Seery et al. (65), and reports of preliminary efficacy in Seery et al., (66). Additionally, Patrickson et al. (171), highlighted their intention to adhere to the Consolidated Framework for Implementation Research (206), which again emphasizes understanding integration alongside efficacy.

Other examples across the NICE categories may benefit from adhering to this approach. We would highlight the particular importance of these frameworks when considering diagnostic technologies. Using an implementation framework would help elucidate how these DHTs might be suitably applied to improve diagnosis rather than undermine, replace, or obfuscate the clinician's perspective. Previous investigations into the QbTest + for childhood ADHD diagnosis have evaluated the impact of a diagnostic-oriented DHT on clinician workflow (191) and given the expansive rollout now underway across the UK healthcare service (207), there may be opportunities to apply best practices that support the provision of similar technologies to adults with ADHD.

### 4.4 Limitations

There are several limitations in our review to note. Firstly, as a Scoping Review, we do not draw conclusions on the overall efficacy of any of the technologies presented here. As such, the review does not make recommendations or provide implications for direct clinical practice or policy. Rather, we identify and map the current research to provide an up-to-date view of the environment surrounding DHTs for adults with ADHD. Through our analysis, we identify gaps and concerns, which future research should address to provide a robust evidence base that enables implementation of these technologies into a clinical setting.

Secondly, the objective for our research question, and resulting search strategy, was to provide a breadth of DHTs to assess, rather than an in-depth focus on any single category. Previous reviews exist which provide a concerted focus on DHT categories, e.g., (37, 39, 201). However, this review is the first to look more broadly across all stages of the care pathway, enabling us to identify areas where DHTs are more frequently being developed. We justify our focus on adulthood ADHD given that it is currently managed distinctly from pediatric services, and the technologies are more suitably evaluated against the challenges that are faced in adulthood.

Thirdly, we have used three key tools to set the remit of the review. We define a “digital health technology” as a digital tool which provides a health benefit (40), yet there may be examples in the literature which we did not include if their health benefits, direct or otherwise, were not stated or sufficiently described. Next, we used the NICE DHT Framework for classification, which is based on the intended use of a technology in a clinical setting. Despite this, many papers describe results from an applied research setting and do not contextualize how their DHT is situated within a clinical setting. For the purposes of this review, we derived a primary category based on how the technologies were described by the authors, but these categories should not be considered definitive and may differ if implemented clinically. Lastly, we used the NIH Quality Assessment tools to undertake our RoB analysis, which lack validation. This choice is justified considering our objective was not to evaluate efficacy but instead identify trends across a heterogeneous set of study designs. The use of the NIH tools, which have tailored options for each type of study design under a single framework supported this. Future works which systematically examine efficacy may benefit from confirming these trends in particular subcategories using validated tools (49–51).

### 4.5 Conclusion

DHTs offer the potential to provide scalable and efficient solutions that improve access to suitable healthcare for adults with ADHD. This review identified numerous examples of existing research into technologies that could be situated throughout the care pathway to support diagnosis, treatment, monitoring, and self-management of ADHD in adulthood. Our review has highlighted that most DHTs are being developed to support clinical care, being categorized within treatment, diagnosis, and clinical management. However, the outcome measures and sources of bias we identify suggest that these DHTs could be improved by developing and evaluating them with a greater consideration of how they will be adopted into clinical settings and how they will be used by people with ADHD in real-world settings.

For DHTs to realize their potential, we highlight the need for future research that draws more broadly on complementary and multidisciplinary research methods that effectively capture the considerations of all individuals involved in the development and delivery of the technologies. This needs to be combined with robust investigations into participant experiences, and direct participant involvement in the design process. We highlight various frameworks which promote the view that interventions should extend their investigations beyond efficacy and consider the interactions between the technology and the people and systems that currently operate the care pathway.

## Data Availability

The original contributions presented in the study are included in the article/Supplementary Material, further inquiries can be directed to the corresponding author.

## Glossary

ADHD: attention-deficit/hyperactivity disorder
ADHD: rating scale (ADHD-RS)
ADHD: self assessment scale (ADHD-SB)
APA: American Psychiatric Association
ASRS: adult adhd self report scale
AUC: area under curve
BDEF: Barkley deficits in executive functioning
CAARS: Conners adult adhd rating scale
CAS: case series
CBT: cognitive behavioral therapy
CD: cannot determine
CFQ: cognitive failures questionnaire
CIV: controlled interventions
CPT: continuous performance test
CSS: current symptoms self-report scale
dACC: dorsal anterior cingulate cortex
DBT: dialectical behavioral therapy
DHI: digital health intervention
DHT: digital health technology
dlPFC: dorsolateral prefrontal cortex
DSM: diagnostic and statistics manual
EEG: electroencephalography
fNIRS: functional near-infrared spectroscopy
ICC: intraclass correlation coefficient
ICD-11: International classification of diseases—11th edition
IDA-R: integrated diagnosis of ADHD—revised
MRC: medical research council
MRI: magnetic resonance imaging
NA: not applicable
NF: neurofeedback
NICE: National Institute of Health and Care Excellence
NIH: National Institute of Health
NR: not reported
OCS: observational and cross-sectional
PAPM: precaution adoption process model
PPNC: pre-post with no control
PRISMA-ScR: preferred reporting items for systematic reviews and meta-analyses—scoping review extension
RCT: randomized controlled trial
RoB: risk of bias
ROC: receiver operating characteristics
rTMS: repetitive transcranial magnetic stimulation
SD: standard deviation
SMS: short messaging service
tDCS: transcranial direct current stimulation
VR: virtual reality
WAIS: Wechsler adult intelligence scale
WMS: Wechsler memory scale
WHO: World Health Organization
WURS: Wender-Utah rating scale
