# Supplementary material for: Digital health technologies for adults with ADHD: a scoping review
Source: Front Digit Health. 2026 Feb 23;8:1746732. doi: 10.3389/fdgth.2026.1746732 (PMC12969067; doi:10.3389/fdgth.2026.1746732)
Supplement: Supplementary file 1 [file Table1.docx]

Supplementary Material

# Supplementary Data

## Protocol Registration

The protocol for the systematic review is available from https://osf.io/tk3pm. The registration adheres to the Generalized Systematic Review Registration Form provided by the Open Science Framework (OSF).

## Deviations from Protocol

While the original protocol did not specify a RoB assessment – which is consistent with a Scoping Review methodology – the decision to incorporate RoB analysis emerged during the review process. This decision arose during the evidence-mapping stage, where RoB analysis was identified as an appropriate mechanism that would strengthen the interpretability of our findings. In particular, it enabled us to objectively examine the mechanisms by which Digital Health Technologies would lead the measurable health benefits. Given that these mechanisms are reliant on the validity of the included studies, our RoB assessment provided a structured approach to identifying bias.

## Search Terms

Not that PsycINFO and Medline were accessed through Ovid as the primary interface. For CINAHL, the EBSCO interface was used.

### PubMed

4 #1 AND #2 AND #3

3 "Telemedicine"[MeSH] OR "Digital Health"[MeSH] OR "software"[MeSH] OR "technolog*" OR "virtual" OR "remote" OR "assistive" OR "smartphone" OR "comput*" OR "mHealth" OR "m health" OR "smart-*" OR "self-help" OR "robot*" OR "automat*" OR "app" OR "internet" OR "mobile device" OR "digit*" OR "sensor" OR "tablet" OR "portable" OR "interface" OR "virtual reality" OR "VR"

2 Attention Deficit Disorder With Hyperactivity[MeSH]

1 Adult[MesH]

### Medline (Ovid)

1. exp Attention Deficit Disorder with Hyperactivity/

2. exp Adult/

3. Microcomputers/ or Computers, Handheld/ or Smart Glasses/ or Minicomputers/

4. Smartphone/ or Text Messaging/

5. exp Telemedicine/ or Digital Health/

6. exp Virtual Reality/

7. exp Self-Help Devices/

8. (technolog* or virtual or remote or assistive or smartphone or comput* or mhealth or "m health" or Smart* or self-help or robot* or automat* or app or internet or mobile device or digit* or sensor or tablet or portable or interface or virtual reality or VR).mp. [mp=title, book title, abstract, original title, name of substance word, subject heading word, floating sub-heading word, keyword heading word, organism supplementary concept word, protocol supplementary concept word, rare disease supplementary concept word, unique identifier, synonyms, population supplementary concept word, anatomy supplementary concept word]

9. exp Software/

10. 3 or 4 or 5 or 6 or 7 or 8 or 9

### PsycINFO (Ovid)

1. exp Attention Deficit Disorder/

2. Adult.mp. or (Emerging Adulthood/ or Late Adulthood/ or Middle Adulthood/)

3. exp Technology/

4. exp Computers/ or exp Mobile Devices/ or exp Laptop Computers/ or exp Tablet Computers/

5. (virtual or technolog* or automat* or app or online or digit* or portable or interface or internet or assistive or app or sensor or tablet or smart*).mp. [mp=title, abstract, heading word, table of contents, key concepts, original title, tests & measures, mesh word]

6. 3 or 4 or 5

7. 1 and 2 and 6

### Cochrane

#1 MeSH descriptor: [Attention Deficit Disorder with Hyperactivity] explode all trees

#2 MeSH descriptor: [Adult] explode all trees

#3 MeSH descriptor: [Telemedicine] explode all trees

#4 MeSH descriptor: [Digital Health] explode all trees

#5 MeSH descriptor: [Software] explode all trees

#6 technolog* OR virtual OR remote OR assistive OR smartphone OR comput* OR mHealth OR "m health" OR smart* OR "self help" OR robot* OR automat* OR app OR internet OR "mobile device" OR digit* OR sensor OR tablet OR portable OR interface OR virtual reality OR VR

#7 #1 AND #2 AND (#3 OR #4 OR #5 OR #6)

### CINAHL

S3 (TX ( technolog* or comput* or tablet or mobile phone or smartphone or internet or virtual or VR or automat* or app or online or digit* or portable or interface or internet or assistive or sensor or smart or telemedicine or "digital health" or "self-help")) AND (S1 AND S2)

S2 TX ( technolog* or comput* or tablet or mobile phone or smartphone or internet or virtual or VR or automat* or app or online or digit* or portable or interface or internet or assistive or sensor or smart or telemedicine or "digital health" or "self-help" or ehealth or mhealth or mobile health or "electronic intervention")

S1 AG ( adults or adult ) AND MH attention deficit hyperactivity disorder

### ACM Digital Library

[[All: "adhd"] OR [All: "attention-deficit/hyperactivity disorder"] OR [All: "attention deficit disorder"] OR [All: "attention deficit disorder with hyperactivity"] OR [All: "hyperkinetic syndrome"]] AND [[All: "adult"] OR [All: "aged"] OR [All: "young adult"] OR [All: "middle aged"] OR [All: "elderly"]]

### IEEE Xplore

"All Metadata":"ADHD" OR "All Metadata":"attention-deficit/hyperactivity disorder" OR "All Metadata":"attention deficit disorder" OR "All Metadata":"attention deficit disorder with hyperactivity" OR "All Metadata":"hyperkinetic syndrome") AND ("All Metadata":"Adult" OR "All Metadata":"aged" OR "All Metadata":"Young adult" OR "All Metadata":"middle aged" OR "All Metadata":"elderly"

### Web of Science

((ALL=("Attention Deficit Hyperactivity Disorder" OR "ADHD" OR "Attention Deficit Disorder with Hyperactivity" OR "attention deficit disorder" OR "hyperkinetic syndrome")) AND ALL=("adult" OR "young adult" OR "aged" OR "middle Aged" OR "elderly")) AND ALL=("telemedicine" OR "digital health" OR technolog* OR virtual OR remote OR assistive OR smartphone OR comput* OR mHealth OR smart* OR "self-help" OR robot* OR automat* OR app OR internet OR "mobile device" OR digit* OR sensor OR tablet OR portable OR interface OR "virtual reality" OR VR)

### Scopus

( TITLE-ABS-KEY ( "Attention Deficit Hyperactivity Disorder" OR "ADHD" OR "Attention Deficit Disorder with Hyperactivity" OR "Attention Deficit Disorder" OR "Hyperkinetic Syndrome" ) ) AND ( TITLE-ABS-KEY ( "Adult" OR "early adulthood" OR "middle aged" OR "aged" OR "young adult" OR "elderly" ) ) AND ( ALL ( telemedicine OR "digital health" OR software OR technolog* OR virtual OR remote OR assistive OR smartphone OR comput* OR mhealth OR "m health" OR smart* OR "self-help" OR robot* OR automat* OR app OR internet OR "mobile device" OR digit* OR sensor OR tablet OR portable OR interface OR virtual AND reality OR vr ))

# Supplementary Tables

**Table S1:** Average Age, Study Size, and Percentage Males for Each NICE DHT Classification

|  | **Treat a Specific Condition** | **Drive Clinical Management** | **Diagnose a Specific Condition** | **Inform Clinical Management** | **Promoting Good Health** | **Communicate about Health and Care** |
| --- | --- | --- | --- | --- | --- | --- |
| **Average Age (SD)** | 32.56 (6.81) | 29.99 (7.71) | 28.74 (6.29) | 32.15 (5.78) | 32.08 (7.79) | N/G (N/A) |
| **Average Study Size (SD)** | 70 (95.58) | 133 (144.98) | 133 (106.78) | 135 (178.13) | 18 (16.50) | 117 (N/A) |
| **Average % Male (SD)** | 45.54 (15.23) | 54.50 (20.24) | 49.08 (15.68) | 39.51 (11.96) | 37.22 (4.12) | 66.7 (N/A) |
| Acronyms: N/A: Not Applicable, N/G: Not Given | | | | | | |

Table S2: List of papers with key information extracted for papers classified as Treat Specific Condition - Web and App-Based Cognitive Therapy or Psychoeducation.

| **Study** | **Study Size** | **Study Design** | **Age**  **(Mean + SD)** | **% Male** | **Target Domain** | **Primary Outcome(s)** | **Secondary Outcome(s)** | **Method of Delivery** | **Guided or Self-Guided** | **Duration** |
| --- | --- | --- | --- | --- | --- | --- | --- | --- | --- | --- |
| Ahlers et al., (2022) (1) | 367  (94 with ADHD) | CI | 27.9  (7.5) | 71.6 | Cannabis Use Disorder | Cannabis Use in Past 30 Days | CUDIT  SeDS  ASRS  GAD-7  CES-D  Retention  Intervention adherence  Safety | Web | Guided | 6 Weeks |
| Antshel et al., 2025 (2) | 154 | CI | 36.15  (7.5) | 22.07 | Executive Functioning | ASRS | BFIRS  AAQoL  OTMP  DERS  ADHD-Cog  MAUQ | App | Self Guided | 8 Weeks |
| Champ et al., (2025) (3) | 23 | CI | 33.35  (10.1) | 56.52 | Motivation and Strategy Development | EQ-5D-5L  CORE-OM  ADHD-RS  AAQoL  SR&I  IAF | Feasibility | Web | Guided | 10 Weeks |
| Flobak et al., (2021) (4) | Study 1: 12 Study 2: 109 | OCS | 36.00  (9) | 19 | ADHD-Related Challenges | Qualitative Feedback for Design Recommendations | - | Web | Self Guided | 7 Weeks |
| Galili-Simhon et al., (2023) (5) | 5 | PPNC | 26.8  (1.92) | 40 | Executive Functioning | Recruitment Rate Retention Adherence | BRIEF-A AAQoL Acceptability | Video Conferencing | Guided | 16 Weeks |
| Kenter et al., (2023) (6) | 120 | CI | 40.9  (10.6) | 20 | ADHD-related challenges | ASRS | AAQoL  PSS User Satisfaction Treatment Adherence | Web | Self Guided | 7 Weeks |
| Knouse et al., (2022) (7) | 240 | CS | 29.15  (7.14) | 21.3 | ADHD-related challenges | Usability Feasibility | BAARS BFIRS | App | Self Guided | 7 Weeks |
| Moëll et al., (2015) (8) | 57 | CI | 36.8  (10.95) | 31.5 | Organisational Skills | ASRS Inattention subscale | ASRS - Hyperactivity subscale SDS HADS PSS | Web | Guided | 6 Weeks |
| Nasri et al., (2023) (9) | 104 | CI | 36.59  (10.36) | 32 | ADHD-related challenges | ASRS | Alcohol use Drug Use Depression Emotion Regulation Stress Quality of Life Sleep Difficulties | Web | Guided | 12 Weeks |
| Nordby et al., (2021) (10) | 13 | PPNC | 39.2  (10.2) | 46.15 | ADHD-related challenges | Feasibility Adherence | ASRS  PSS  PHQ  GAD  AAQoL | Web | Self Guided | 7 Weeks |
| Nordby et al., (2022) (11) | 109 | CI | 36.10  (9.1) | 19.3 | ADHD-related challenges | Adherence | Module Completion  Logins  Minutes Spent Online  Use of Coping Strategies | Web | Self Guided | 7 Weeks |
|  |  |  |  |  |  |  |  |  |  |  |
| Nordby et al., (2024) (12) | 16 | PPNC | 30.4 (11.7) | 37.5 | Emotion Regulation | Adherence  Feasibility  CEQ  ASRS | - | App | Companion to Traditional Therapy | 6 Weeks |
|  |  |  |  |  |  |  |  |  |  |  |
| Nordby et al., (2025) (13) | 9 | PPNC | 40.8 (Range 28-65) | 33.33 | Emotion Regulation | Qualitative Analysis of Participant Experience | - | App | Companion to Traditional Therapy | 6 Weeks |
| Oscarsson et al., (2025) (14) | 36 | PPNC | 44.4 (8.29) | 20.93 | Quality of Life | AAQoL | PSS; KEDS; GAD-7; PHQ-9; ASRS | Web | Guided | 12 Weeks |
| Pettersson et al., (2017) (15) | 45 | CI | 37.09  (10.35) | 35.57 | ADHD-related challenges | CSS | BDI  BAI ADHD Impact Module – Adult  COPM | Web | Self Guided | 10 Weeks |
| Scholl et al., (2021) (16) | 33 (11 with ADHD) | PPNC | N/G | N/G | ADHD-related challenges | Client Satisfaction Questionnaire | WHOQOL-BREF PHQ-9 CEQ | Web | Guided | 4 Weeks |
| Seery et al., (2023) (17) | 49 | OCS | 43.18  (9.75) | 20.93 | Wellbeing | Qualitative Analysis of Feasibility and Acceptability | - | Web | Guided | 6 Weeks |
| Seery et al., (2024) (18) | 257 | PPNC | 41.16  (10.07) | 22.18 | Wellbeing | Acceptability Attrition | AAQoL Psychological Flexibility Disability Self-Acceptance Scale Knowledge of ADHD | Web | Guided | 6 Weeks |
| Sehlin et al., (2018) (19) | 16 | OCS | 23.00  (5) | 56 | ADHD-related challenges | Qualitative Analysis of Feasibility and Acceptability | - | Web | Guided | 8 Weeks |
|  |  |  |  |  |  |  |  |  |  |  |
| Selaskowski et al., (2022) (20) | 43 | CI | 38.2 (11.9) | 53.49 | ADHD-Related Challenges | IDA-R | IDA-R Hyperactivity Subscale IDA-R Impulsivity Subscale  BDI | App | Companion to Traditional Therapy | 8 Weeks |
|  |  |  |  |  |  |  |  |  |  |  |
| Selaskowski et al., (2023) (21) | 34 | CI | 29.65 (8.55) | 47.1 | ADHD-Related Challenges | IDA-R; ADHD-SB | DASS-21  WHOQOL | Chatbot | Self Guided | 3 Weeks |
| Shelton et al., (2022) (22) | 235 | OCS | 27.54  (4.29) | 45.1 | ADHD-related challenges | Acceptability CEQ | - | Web | Self Guided | Unstructured |
| Tsirmpas et al., (2023) (23) | 30 | PPNC | 31.34  (6.44) | 67 | Anxiety and Depression | Participant Satisfaction Usability | PHQ-9 GAD-7 BAARS LISAT-11 (Life Satisfaction) SWLS  Self-Assessment Questionnaire User Feedback Survey | App | Self Guided | 16 Weeks |
| Ulusoy et al., (2024) (24) | 32 | CI | N/G | 37.5 | ADHD-related challenges | Attrition Adherence | ASRS BIS-11 MAAS  Stressful Life Events | Video Conferencing | Guided | 12 Weeks |
| Wentz et al., (2012) (25) | 12 | PPNC | 19.58  (4.25) | 58.33 | ADHD-related challenges | MANSA  HADS SOC RSES Patient Perspective On Care and Rehabilitation | - | Web | Guided | 8 Weeks |
| Wyler et al., (2021) (26) | 60 | OCS | 38.86  (11.12) | 57.3 | ADHD-related challenges | Client Satisfaction Questionnaire Session Evaluation Questionnaire Working Alliance Inventory  Global Assessment of Functioning | - | Video Conferencing | Guided | Unstructured |
| Zhang et al., (2025) (27) | 86 | CI | 25  (N/G) | 47.67 | ADHD-related challenges | ADHD-RS | Self-Rating Anxiety Scale  BRIEF-A  Stroop Colour Word Test  CPT  WHOQOL-BREF  SDS | Video Conferencing | Guided | 12 Weeks |
| **Acronyms:** ADHD Cognition Scale (ADHD-Cog), ADHD Rating Scale (ADHD-RS), ADHD Self Assessment Scale (ADHD-SB), Adult ADHD Quality of Life (AAQoL), Adult ADHD Self-Report Scale (ASRS), Barkley Adult ADHD Rating Scale (BAARS), Barkley Functional Impairment Scale (BFIRS), Barratt Impulsiveness Scale (BIS-11), Beck Anxiety Inventory (BAI), Beck Depression Inventory (BDI), Behavior Rating Inventory of Executive Function (BRIEF-A), Canadian Occupational Performance Measure (COPM), Cannabis Use Disorder Identification Test (CUDIT), Case Series (CS), Centre for Epidemiological Studies Depression Scale (CES-D), Clinical Outcomes in Routine Evaluation – Outcome Measure (CORE-OM), Continuous Performance Test (CPT), Controlled Intervention (CI), Current Symptoms Scale Self-Report Form (CSS), Deficits in Emotion Regulation Scale (DERS) , Depression, Anxiety, and Stress Scale-21 (DASS-21), Generalized Anxiety Disorder Scale (GAD-7), Hospital Anxiety and Depression Scale (HADS), Index of Autonomous Functioning (IAF), Integrated Diagnosis of ADHD – Revised (IDA-R), Karolinska Exhaustion Disorder Scale (KEDS), Life Satisfaction Questionnaire-11 (LISAT-11), Manchester Short Assessment of Quality of Life (MANSA), mHealth App Usability Questionnaire (MAUQ), Mindful Attention Awareness Scale (MAAS), Observational and Cross-Sectional (OCS), Patient Health Questionnaire-9 (PHQ-9), Perceived Stress Scale (PSS), Pre-Post with No Control (PPNC), Rosenberg Self-Esteem Scale (RSES), Satisfaction With Life Scale (SWLS), Self-Reflection and Insight Scale (SR&I), Sense of Coherence Scale (SOC), Severity of Dependence Scale (SeDS), Sheehan Disability Scale (SDS), Treatment Credibility and Expectancy (CEQ), World Health Organization Quality of Life (WHOQOL), World Health Organization Quality of Life – Brief (WHOQOL-BREF) | | | | | | | | | | |

Table S3: List of papers with key information extracted for papers classified as Treat Specific Condition - Cognitive Training Programs

| **Study** | **Study Size** | **Study Design** | **Age** | **% Male** | **Target Domain** | **No. Of Sessions** | **Blinded/**  **Unblinded** | **Control Condition** | **Near Transfer Measures** | **Far Transfer Measures** |
| --- | --- | --- | --- | --- | --- | --- | --- | --- | --- | --- |
| Cunha et al., (2023) (28) | 25 | CI | 20.96  (0.84) | 4.15 | Memory Cognitive Flexibility Processing Attention | 10x 30min (twice weekly) | Unblinded | Wait-List Control | Processing Speed Sequence of Letters and Numbers Spatial Location | - |
| Kolodny et al., (2017) (29) | 30 | CI | 25.1  (2.7) | 43.30 | Sustained Attention Selective-Spatial Attention Orienting Attention Executive Attention | 16x 1hr (twice weekly) | Unblinded | Computer Games Control | CPT Performance metrics Visual search task Stroop-like task | MATAL ADHD Questionnaire |
| Lintas et al., (2025) (30) | 106 | CI | 22.75 (0.54) | 59.17 | Working Memory | 18 x 30min (daily) | Unblinded | Fixed 1-Back Task | WAIS-IV Working Memory Scale  Corsi N-back Task | - |
| Marcelle et al., (2018) (31) | 13 | PPNC | 27.75  (5.6) | 53.85 | Working Memory | 25 x 45min (five times weekly) | Unblinded | Uncontrolled | - | - |
| Mawjee et al., (2014) (32) | 38 | CI | 23.39  (4.02) | 52.60 | Working Memory | 25x 45min (five times weekly) | Unblinded | Controlled  Shortened-Task (15 min) / Waitlist | Digit Span Spatial Span WRAML - Finger Windows | ASRS CFQ BDEF |
| Mawjee et al., (2015) (33) | 97 | CI | 23.9  (3.41) | 40.20 | Working Memory | 25 x 45 min (five times weekly) | Unblinded | Controlled  Shortened-Task (15 min) / Waitlist | Digit Span Spatial Span WRAML - Finger Windows | ASRS CFQ  BDEF  Math Fluency Word Reading Efficiency |
| Osman et al., (2025) (34) | 143806* (*Naturalistic Real-World Data collection) | OCS | 50.90 (16.87) | 36.83 | Executive Functioning | Varied | Unblinded | Uncontrolled | NCPT | BAMS-7 |
| Selaskowski et al., (2023) (35) | 36 (18 with ADHD) | CI | 31  (6.9) | 63.85 | Inattention | 1x 18 min | Single-Blinded | Controlled  Sham-Feedback | CPT performance metrics | - |
| Sjöwall et al., (2023) (36) | 24 | PPNC | 36.75  (8.37) | 45.80 | Working Memory | 25 x 45 min (five times weekly) | Unblinded | Uncontrolled | Spatial Span | ASRS CFQ BDEF |
| Stern et al., (2016) (37) | 60 | CI | 37.31  (10.11) | 43.33 | Working Memory Inhibition Shifting Selected and Divided Attention Persistence | 48 x 20 min (4-5 times a week) | Double-Blind | Controlled  Non-Hierarchical CT with less executive demand | CPT performance metrics | BRIEF-A ASRS COPM AAQoL |
| Stern et al., (2012) (38) | 14 | PPNC | 33.50 (9.27) | 71.43 | Working Memory Inhibition Shifting Selected and Divided Attention Persistence | 48 x 20 min (4-5 times a week) | Unblinded | Uncontrolled | CPT Performance Metrics | ASRS WURS BRIEF-A COPM AAQoL |
| Stern et al., (2023) (39) | 51 | CI | 27.77 (4.37) | 43.14 | Sustained Attention Selective-Spatial Attention Orienting Attention Executive Attention | 8x 120 min (weekly) | Unblinded | Controlled  MBSR | CPT Performance Metrics | ASRS |
| Woltering et al., (2021) (40) | 89 | CI | 23.77 (3.45) | 55.06 | Working Memory | 35 x 45min (Daily) | Unblinded | Controlled  Shortened-Task (15 min) / Waitlist | EEG Metrics of working memory Digit Span Pattern Recognition | ASRS SA-45 |
| Adult ADHD Quality of Life Scale (AAQoL), Adult ADHD Self-Report Scale (ASRS), Barkley Deficits in Executive Functioning (BDEF), Behavior Rating Inventory of Executive Function (BRIEF-A), Brief Attention and Mood Scale (BAMS-7), Canadian Occupational Performance Measure (COPM), Cognitive Failures Questionnaire (CFQ), Controlled Intervention (CI), Mindfulness-Based Stress Reduction (MBSR), Neurocognitive Performance Test (NCPT), Observational and Cross-Sectional (OCS), Pre-Post with No Control (PPNC), Symptom Assessment-45 (SA-45), Wender-Utah Rating Scale (WURS), Wide Range Assessment of Memory and Learning (WRAML) | | | | | | | | | | |

Table S4: List of papers with key information extracted for papers classified as Treat Specific Condition – Neurofeedback.

| **Study** | **Study Size** | **Study Design** | **Age (Mean + SD)** | **% Male** | **Imaging Device** | **Parameter of Brain Activity** | **No. Of Sessions** | **Blinded/**  **Unblinded** | **Control-Arm Condition** | **Primary Outcomes** | **Secondary Outcomes** |
| --- | --- | --- | --- | --- | --- | --- | --- | --- | --- | --- | --- |
| Cowley et al., (2016) (41) | 54 | CI | 36.11  (10.30) | 46 | EEG | Theta-Beta Ratio Sensorimotor Rhythm | 40 x 1-hour | Unblinded | Wait-List | Learning Curve Assessment TOVA ASRS  Digit Span | Vigilance (EEG protocol)  Circadian Pattern Assessment Mood  Excitement  Effort  Frustration  PSQI |
| Esmaeilzadeh et al., (2021) (42) | 11 | PPNC | 25  (6.82) | 54.55 | EEG | Sensorimotor Rhythm | 20 x 1-hour | Unblinded | Uncontrolled | Functional Connectivity | - |
| Hudak et al., (2018) (43) | 19 | PPNC | 30.37  (9.25) | 68.42 | fNIRS | Prefrontal HbO2 Concentration | 30 x 32 Min | Unblinded | Uncontrolled | Functional Connectivity Learning Rates | Event-Related Potentials |
| Mayer et al., (2012) (44) | 18 | PPNC | 27.56  (3.35) | 61.11 | EEG | Slow Cortical Potential | 15 x 32 min | Unblinded | Uncontrolled | ADHD-SB BDI | Contingent Negative Variant |
| Ochi et al., (2017) (45) | 17 | PPNC | Range 18-30 | N/G | EEG | Proprietary "Attention" Algorithm | 20 x 20 min | Unblinded | Uncontrolled | TOVA | - |
| Schonenberg et al., (2017) (46) | 118 | CI | 37.95  (11.04) | 56.67 | EEG | Theta-Beta Ratio | 15 x 32 min | Tripled-Blinded (Participants, Administrator, Assessors) | Sham-Control + Cognitive Behavioural Therapy | CAARS | BDI  STAI |
| Veilahti et al., (2021) (47) | 23 | CI | 35.7  (9.7) | 43.48 | EEG | Theta-Beta Ratio  Sensorimotor Rhythm | 40 x 1hr | Unblinded | Wait-List | Learning Rate | TOVA  ASRS |
| Whitehead et al., (2022) (48) | 593 | PPNC | 37.25  (11.9) | 54.6 | EEG | Theta-Beta Ratio  Alpha | Varied | Unblinded | Uncontrolled | GHQ-12  ASRS  GAD-7  PHQ-9 | Continuous Performance Test, Resting-State EEG Markers |
| Zilverstand et al., (2017) (49) | 13 | CI | 36.9  (13) | 46.15 | fMRI | dACC Activation Levels | 4 x 90 min | Single-Blinded (Participant) | Mental Arithmetic Challenge | MSIT SADT SART  2-Back Working Memory Digit Span Letter Number Sequencing Vocabulary Block Design | - |
| **Acronyms:** Adult ADHD Self-Report Scale (ASRS), Attention Deficit Hyperactivity Disorder Self-Assessment Scale (ADHD-SB), Beck Depression Inventory (BDI), Conner’s Adult ADHD Rating Scale (CAARS), Controlled Intervention (CI), Electroencephalography (EEG), Functional Magnetic Resonance Imaging (fMRI), Functional near-infrared spectroscopy (fNIRS), General Health Questionnaire-12 (GHQ-12), Generalized Anxiety Disorder Scale-7 (GAD-7), Multi Source Inference Task (MSIT), Patient Health Questionnaire-9 (PHQ-9), Pittsburgh Sleep Quality Index (PSQI), Pre-Post with No Control (PPNC), State Trait Anxiety Inventory (STAI), Sustained Attention Dots Task (SADT), Sustained Attention to Response Task (SART), Test of Variables of Attention (TOVA) | | | | | | | | | | | |

Table S5: List of papers with key information extracted for papers classified as Treat Specific Condition – Transcranial Direct / Alternate Current Stimulation and Repetitive Transcranial Magnetic Stimulation.

| **Study** | **Study Size** | **Study Design** | **Age (Mean + SD)** | **% Male** | **Region Targeted** | **No. Of Sessions** | **Stimulation Intensity** | **Blinded**  **/Unblinded** | **Control Condition** | **Primary Outcomes** | **Secondary Outcomes** |
| --- | --- | --- | --- | --- | --- | --- | --- | --- | --- | --- | --- |
| Barham et al., (2022) (50) | 22 | CI | 22 (2.77) | 22 | Anodal right dlPFC,  Cathodal left dlPFC | 5 x 20 min (Daily) | 2mA | Double-Blind | Sham-Control | Digit Span Trail Making Test  Stroop Test CPT Tower of London Benton Face Recognition  Reading the Mind in the Eyes | - |
| Bleich-Cohen et al., (2021) (51) | 62 | CI | 35.1 (9) | 35.1 | Deep TMS Right PFC  Deep TMS Left PFC | 15 x 15min (daily) | 120% of Rest Motor threshold | Double-Blind | Sham-Control | CAARS  CGI | fMRI changes in Activity |
| Bloch et al., (2010) (52) | 13 | CI | N/G | NG | Right dlPFC | 1x 22min | 100% of Rest Motor Threshold | Double-Blind | Sham-Control | PANAS | VAS – Attention  VAS - Mood CANTAB |
| Cachoieira et al., (2017) (53) | 17 | CI | 32.29 (4.98) | 32.29 | Anodal right dlPFC,  Cathodal left dlPFC | 5x 20min (Daily) | 2mA | Double-Blind | Sham-Control | ASRS | SDS |
| Dallmer-Zerbe et al., (2020) (54) | 18 | CI | 31.3 (9.89) | 31.3 | Areas of the brain involved in P300 Waveform generation | 1x 20 Min | 1mA | Single-Blind | Sham-Control | P300 Amplitude Modulation Go/Nogo Task | - |
| Dubreuil-Vall et al., (2021) (55) | 40 | CI | 37.35 (13.87) | 37.35 | Anodal right dlPFC, Cathodal left dlPFC | 1x 30min | 2mA | Double-Blind | Sham-Control | Eriksen Flanker Task Stop-Signal Task | Event-related potentials |
| Fu et al., (2025) (56) | 56 | CI | 26.73 (4.95) | 31.3 | Frontoparietal Regions | 20x 40min | 15mA (tACS) | Double-Blind | Sham-Control | ASRS  BRIEF-A | WFIRS  PSQI  HAMD-17  HAMA  CGI |
| Kannen et al., (2024) (57) | 24 | CI | 32.25 (10.46) | 32.25 | Posterior Brain Regions | 1x 18min | 1.5mA | Single-Blind | Sham-Control | Alpha Power Modulation CPT Performance Metrics Virtual Reality Sickness Questionnaire | Subjective symptom ratings on inattention, impulsivity, and hyperactivity |
| Leffa et al., (2022) (58) | 64 | CI | 38.3 (9.6) | 38.3 | Anodal right dlPFC, Cathodal left dlPFC | 28x30min (daily) | 2mA | Double-Blind | Sham-Control | ASRS | - |
| Paz et al., (2018) (59) | 26 | CI | 31.6 (6.65) | 31.6 | Bilateral Prefrontal Regions | 20x 20min (daily) | 120% of measured motor threshold | Double-Blind | Sham-Control | CAARS  TOVA | - |
| Rodrigues et al., (2024) (60) | 29 | CI | 37.8 (10.5) | 37.8 | Anodal right dlPFC, Cathodal left dlPFC | 28 x 30 min (daily) | 2mA | Double-Blind | Sham-Control | ASRS – Inattention Subscale | - |
| Schneider et al., (2025) (61) | 55 | CI | 38.1 (9.8) | 60 | Anodal right dlPFC, Cathodal left dlPFC | 28 x 30min (daily) | 2mA | Double-Blind | Sham-Control | Goal Attainment Scale | - |
| **Acronyms:** Adult ADHD Self Report Scale (ASRS), Behavior Rating Inventory of Executive Functioning (BRIEF-A), Cambridge Automated Neuropsychological Battery (CANTAB), Clinical Global Impression Scale (CGI), Conners Adult ADHD Rating Scale (CAARS), Controlled Intervention (CI), Dorsolateral Prefrontal Cortex (dlPFC), Hamilton Anxiety Scale (HAMA), Hamilton Depression Scale (HAMD-17), Positive and Negative Affect Scale (PANAS), Sheehan Disability Scale (SDS), Transcranial Magnetic Stimulation (TMS), Visual Analogue Scale (VAS), Weiss Functional Impairment Scale (WFIRS) | | | | | | | | | | | |

Table S6: List of papers with key information extracted for papers classified as Treat Specific Condition – Other.

| **Study** | **Study Size** | **Study Design** | **Age** | **% Male** | **Type of Technology** | **Primary Outcomes** | **Secondary Outcomes** |
| --- | --- | --- | --- | --- | --- | --- | --- |
| Bartlett et al., (2024) (62) | 49 | CI | 21.45 (2.16) | 26.53 | Wearable Device | STAI  EALS | Acceptability  Adherence |
| Praus et al., (2023) (63) | 121 | OCS | 39.09 (12.37) | 37 | Telemedicine | WHO-5  SCL-90R  BDI | Psychosocial Functioning  Feedback Survey |
| Acronym: Beck Depression Inventory (BDI), Controlled Intervention (CI), Everyday Attention to Life Scale (EALS), Observational and Cross-Sectional (OCS), State-Trait Anxiety Inventory (STAI), Symptom Checklist – 90 Item Revised (SCL-90R), World Health Organization Wellbeing Index – 5 (WHO-5) | | | | | | | |

**Table S7**: List of papers with key information extracted for papers classified as Drive Clinical Management.

| **Study** | **Study Size** | **Study Design** | **Age** | **% Male** | **Type of Technology** | **Aspect of Management** | **Primary Outcomes** | **Secondary Outcomes** |
| --- | --- | --- | --- | --- | --- | --- | --- | --- |
| Adamou et al., (2022) (64) | 69 | OCS | 33 (9.9) | 65.2 | Computerised Test with Adjunctive Data Collection | Diagnosis | Sensitivity Specificity PPV NPV | - |
| Amar et al., (2025) (65) | 79 (42 with ADHD) | OCS | N/G | 70.88 | EEG | Diagnosis | Accuracy  Precision  Recall  F1-Score  AUC | - |
| Baghdassarian et al., (2018) (66) | 119 (24 with ADHD) | OCS | SZ: 30.5 (median) ADHD: 29.5 (median) HC: 27.5 (median) | 60.5 | EEG | Diagnosis | Sensitivity Specificity Accuracy PPV  NPV  ROC AUC | |
| Bijlenga et al., (2015) (67) | 145 | PPNC | 31.4  (10.2) | 55.2 | Computerised Test with Adjunctive Data Collection | Medication Effects | QbTest Factor Scores | Fatigue after QbTest Reduction in QbTest Score after medication |
| Bijlenga et al., (2019) (68) | 209 (97 with ADHD) | OCS | 63.84  (5.1) | 45.95 | Computerised Test with Adjunctive Data Collection | Diagnosis | QbTest Factor Scores Sensitivity Specificity PPV  NPV | Effects of background characteristics on score performance |
| Chaim-Avancini et al., (2017) (69) | 133 (67 with ADHD) | OCS | 26.86  (5.86) | 72.18 | MRI | Diagnosis | AUC Accuracy Sensitivity Specificity PPV  NPV  False Positive Rate False Negative Rate False Discovery Rate ROC | - |
| Chen et al., (2021) (70) | 69 | CS | 33.01  (9.93) | 65.2 | Digitized Clinical Assessment Data | Diagnosis | Accuracy AUC ROC | |
| Edebol et al., (2013) (71) | 341 (55 with ADHD  202 controls  84 ADHD 'Normative') | OCS | 32.42  (10.06) | 54.55 | Computerised Test with Adjunctive Data Collection | Diagnosis | QbTest Factor Scores Sensitivity Specificity | Composite Measure of Symptoms GAF ASRS |
| Elbaum et al., (2020) (72) | 85 | OCS | 23.86  (2.43) | 32.95 | Computerised Test with Adjunctive Data Collection | Diagnosis | AUC | Between-Group Difference in Task Performance Impact of distractor types of performance during task |
| Emser et al., (2018) (73) | Study 1: 60 Study 2: 76 | OCS | Study 1: 8.8 (1.3) Study 2: 33.65 (10.65) | 65.79 | Computerised Test with Adjunctive Data Collection | Diagnosis | Accuracy Sensitivity Specificity | Correlations between CAARS and QbTest+ |
| Gounari et al., (2025) (74) | 53 (25 with ADHD) | OCS | 23.87  (3.82) | 46.9 | Virtual Reality | Diagnosis | TMT - VR TMT ASRS | Usability  User Experience  Acceptability SUS  UEQ-S  Service User Technology |
| Groom et al., (2016) (75) | 58 (33 with ADHD) | OCS | 32.33  (10.87) | 68.7 | Computerised Test with Adjunctive Data Collection | Diagnosis | Accuracy Sensitivity Specificity ROC AUC |  |
| Herman et al., (2025) (76) | 345 | OCS | 34.9 (10.61) | 19.14 | Digitized Clinical Assessment Data | Diagnosis | Cohen’s Kappa  Total Agreement  Sensitivity  Specificity  PPV  NPV | Factors contributing to assessment disagreement |
| Jylkka et al., (2023) (77) | 367 (112 with ADHD) | OCS | 31.07  (8.24) | 30 | Computerised Test | Diagnosis | EPELI Task Performance AUC | ROC  Cruiser and Matching Tasks ICAR-16 CPT Performance Metrics  Instruction Recall Task World List Learning |
| Kaur et al., (2018) (78) | 60 (30 with ADHD) | OCS | 20.58  (1.23) | N/G | EEG | Diagnosis | Accuracy | - |
| Kaur et al., (2020) (79) | 97 (47 with ADHD) | OCS | 20.45  (1.20) | 17.53 | EEG | Diagnosis | Accuracy Sensitivity Specificity | - |
| Kim et al., (2021) (80) | 79 (34 with ADHD) | OCS | 25.19  (6.14) | 81.01 | EEG | Diagnosis | Accuracy Sensitivity Specificity | - |
| Lev et al., (2022) (81) | 66 (33 with ADHD) | OCS | 23.32  (2.15) | 30.3 | Computerised Test with Adjunctive Data Collection | Diagnosis | AUC Sensitivity Specificity | - |
| Li et al., (2024) (82) | 22 (10 with ADHD) | OCS | 28.31  (7.35) | 59.1 | Digitized Clinical Assessment Data | Diagnosis | Accuracy Precision Recall F1 Score ROC AUC | Confusion Matrix |
| Lim et al., (2025) (83) | 150 (75 with ADHD) | OCS | 36.55  (1.09) | 36.67 | fNIRS | Diagnosis | Precision Recall Accuracy F1 Score | - |
| Luo et al., (2020) (84) | 72 (36 with ADHD) | OCS | 24.48  (2.15) | 84.72 | MRI | Diagnosis | Accuracy Sensitivity Specificity ROC AUC | |
| Mueller et al., (2011) (85) | 150 (75 with ADHD)  + 17 ADHD in external validation set | OCS | 35.50  (8.60) | 50.67 | EEG | Diagnosis | Accuracy Sensitivity Specificity Positive Predictive Value | Predictive Power (Classification Accuracy) in a Secondary Dataset |
| Muller et al., (2020) (86) | 328 (181 with ADHD) | OCS | 33.45  (10.97) | 72.63 | EEG | Diagnosis | ROC AUC Sensitivity Specificity FPR FNR Accuracy ICC | |
| Nash et al., (2025) (87) | 22 (10 with ADHD) | OCS | 30.98 (7.56) | 63.63 | Digitized Clinical Assessment Data | Diagnosis | Accuracy  Sensitivity  Specificity | - |
| Nobukawa et al., (2021) (88) | 36 (16 with ADHD) | OCS | 34.78  (8.07) | 44.44 | Eye Tracking | Diagnosis | ROC  AUC | - |
| Oh et al., (2023) (89) | 20 (none with ADHD) | OCS | 28.3  (6.5) | 50 | Virtual Reality | Diagnosis | VR-CPT Performance Measures | Theta-Beta Ratio  KIDS-SR  STAI  PSS  Presence Questionnaire Simulator Sickness Questionnaire SUS |
| Park et al., (2016) (90) | 34 (13 with ADHD-IA  21 with ADHD-C) | OCS | 27.88  (3.39) | 58.82 | MRI | Subtype Specification | Accuracy | Predicting ADHD-Related DSM Score |
| Robeva et al., (2004) (91) | 12 (6 with ADHD) | OCS | 20.1  (1.45) | 0 | EEG | Diagnosis | Accuracy | Correlation with WURS |
| Schweigher et al., (2007) (92) | 49 (28 with ADHD) | OCS | 26.46  (3.65) | 100 | Computerised Test | Diagnosis | Mindstreams Go-NoGo   AUC  ROC | - |
| Ulberstad et al., (2020) (93) | Study I: 25 (11 with ADHD) Study II: 142 (69 with ADHD) | OCS | 26.77  (11.42) | Study I 55.8% Study II 48% | Computerised Test with Adjunctive Data Collection | Diagnosis | QbCheck Performance Metrics  ICC  ROC  AUC  Sensitivity  Specificity | - |
| von Polier et al., (2025) (94) | 767 | OCS | 34.4 (10.60) | 50.59 | Digitized Clinical Assessment Data | Diagnosis | AUC  ROC  Precision  Recall  F1-Score |  |
| Wiebe et al., (2023) (95) | 35 (none with ADHD) | OCS | 23.43  (2.87) | 40 | Virtual Reality with Adjunctive Data Collection | Diagnosis | VR-CPT Performance Metrics Theta-Beta Ratio P300 Latency / Amplitude Head Actigraphy Self-Rated Inattention Self-Rated Impulsivity Self-Rated Hyperactivity | - |
| Wiebe et al., (2023) (96) | 75 (50 with ADHD, 25 Medicated) | OCS | 31.36  (9.20) | 59.72 | Virtual Reality with Adjunctive Data Collection | Diagnosis | CPT Performance Metrics Head Actigraphy EEG Theta-Beta Ratio Eye Tracking Metrics fNIRS HbO Levels | - |
| Wiebe et al., (2024) (97) | 86 (43 with ADHD) | OCS | 31.09  (8.18) | 61.63 | Virtual Reality with Adjunctive Data Collection | Diagnosis | Accuracy Sensitivity Specificity | - |
| Yao et al., (2019) (98) | 189 (112 with ADHD) | OCS | 25.94  (4.49) | 0.6243 | MRI | Diagnosis | Accuracy Specificity Sensitivity | - |
| Yousefimehr et al., (2024) (99) | 103 (51 with ADHD) | OCS | Range 17 - 67 | 51.46 | Computerised Test with Adjunctive Data Collection | Diagnosis | Accuracy Precision Recall F1 Score AUC | - |
| **Acronyms:** Adult ADHD Self-Report Scale (ASRS), Area Under Curve (AUC), Electroencephalography (EEG), False Discovery Rate (FDR), False Negative Rate (FNR), False Positive Rate (FPR), Functional near-infrared spectroscopy (fNIRS), Global Assessment of Functioning (GAF), International Cognitive Ability Resource (ICAR-16), Intraclass Correlation Coefficient (ICC), Korean Depressive Symptomology Scale (KIDS-SR), Magnetic Resonance Imaging (MRI), Negative Predictive Value (NPV), Perceived Stress Scale (PSS), Positive Predictive Value (PPV), Receiver Operating Characteristic (ROC), Schizophrenia (SZ), State-Trait Anxiety Inventory (STAI), System Usability Scale (SUS), Trail Making Task (TMT), User Experience Questionnaire – Short (UEQ-S), Wender-Utah Rating Scale (WURS) | | | | | | | | |

**Table S8**: List of papers with key information extracted for papers classified as Diagnose a Specific Condition.

| **Study** | **Study Size** | **Study Design** | **Age** | **% Male** | **Type of Technology** | **Primary Outcomes** | **Secondary Outcomes** |
| --- | --- | --- | --- | --- | --- | --- | --- |
| Abedian et al., (2024) (100) | 79 (42 control + 37 ADHD) | OCS | Range (20-68) | 70.89 | EEG | Accuracy  Sensitivity  Specificity  ROC  AUC  Precision | |
| Ghassemi et al., (2010) (101) | 50 (10 with ADHD) | OCS | 29.79 (6.18) | 52 | EEG | Accuracy | - |
| Hong et al., (2024) (102) | 195 (120 with ADHD  45 Medicated) | OCS | 35.75 (1.13) | 36.92 | fNIRS | Precision Recall F1 Score Accuracy MCC GDR | Feature Selection Optimization |
| Jayawardena et al., (2019) (103) | 14 | OCS | 24.14 (5.35) | 28.57 | Eye Tracking | Precision Recall F1 Score Accuracy | - |
| Kiiski et al., (2020) (104) | 134 (38 with ADHD) | OCS | 31.83 (12.13) | 38.43 | EEG | Prediction of ADHD Symptoms AUC F1 Score Recall Specificity | APR |
| Leontyev et al., (2019) (105) | 100 | CI | 18.81 (1.32) | 42 | Computerised Test | Prediction of ADHD Symptoms | Feature Selection Optimization |
| Liu et al., (2021) (106) | 272 (43 with ADHD) | OCS | N/G | N/G | MRI | Accuracy Precision Recall | - |
| Ma et al., (2025) (107) | 142 (71 with ADHD) + 45 Validation Set | OCS | 22.43 (1.82) 37.02 (6) | 29.2 | Computerised Test with Adjunctive Data Collection | ROC AUC Sensitivity Specificity Youden Index | - |
| Morey et al., (2019) (108) | 368 | OCS | 18.9 (1.38) | 43.7 | Computerised Test | Sensitivity Specificity AUC | ANOVA / ANCOVA between TOAD, self-report measures, and traditional CPTs |
| Namasse et al., (2025) (109) | 354 | OCS | 18 - 22 (Range) | NG | Digitized Clinical Assessment Data | Accuracy Loss MSE MAE RMSE  F1 Score | Feature Selection Optimization |
| Taymourtash et al., (2015) (110) | 21 (10 with ADHD) | OCS | 27.00 (N/G) | N/G | EEG | Accuracy Sensitivity Specificity | |
| Tenev et al., (2014) (111) | 117 (67 with ADHD) | OCS | 33.1 (8.31) | 50.43 | EEG | Accuracy | ADHD Subtype Classification |
| Torgersen et al., (2025) (112) | 31 | OCS | 23.07 (3.54) | 41.94 | EEG | Accuracy  Precision  Recall  F1 Score | - |
| Trinh et al., (2023) (113) | 150 (52 with ADHD) | OCS | NG | N/G | EEG | AUC | - |
| Unal et al., (2019) (114) | 44 (14 with ADHD) | OCS | 43.39 (10.66) | 50 | Computerised Test | ROC AUC | - |
| Wang et al., (2011) (115) | 49 (21 with ADHD) | OCS | 29.44 (8.47) | 87.76 | MRI | Accuracy | - |
| Yao et al., (2021) (116) | 187 (77 with ADHD) | OCS | 25.94 (4.49) | 62.03 | MRI | Accuracy Specificity Sensitivity | - |
| Zhang et al., (2021) (117) | Dataset 1: 83 (41 with ADHD)  Dataset 2: 47 (24 with ADHD) | OCS | Dataset 1: 32.65 (9.97) Dataset 2: 34.82 (9.05) | Dataset 1: 50% Dataset 2: 70.2% | MRI | Accuracy Sensitivity Specificity F1 Score | - |
| Zhang et al., (2023) (118) | 40 (20 with ADHD) | OCS | 27.625 (5.75) | 45 | fNIRS | AUC ROC | - |
| **Acronyms**: Area Under Curve (AUC), Area Under Precision-Recall Curve (APR), Computerised Performance Test (CPT), Controlled Intervention (CI), Electroencephalography (EEG), Functional near-infrared spectroscopy (fNIRS), Geometric Discriminant Ratio (GDR), Magnetic Resonance Imaging (MRI), Matthews Correlation Coefficient (MCC), Mean Absolute Error (MAE), Mean Square Error (MSE), Observational and Cross-Sectional (OCS), Receiver Operating Characteristic (ROC), Route Mean Square Error (RMSE) | | | | | | | |

**Table S9:** List of papers with key information extracted for papers classified as Inform Clinical Management.

| **Study** | **Study Size** | **Study Design** | **Age** | **% Male** | **Type of Technology** | **Primary Outcomes** | **Secondary Outcomes** |
| --- | --- | --- | --- | --- | --- | --- | --- |
| Backer et al., (2024) (119) | n = 13 clinicians n = 397 with ADHD | OCS | 34.2 | 50.1 | Remote Symptom Tracking | Qualitative Analysis of Content Validity Clinical Relevance | Face Validity Kaiser-Meyer-Olkin Sampling Accuracy Bartlett Test of Sphericity Exploratory Factor Analysis |
| Biederman et al., (2020) (120) | 448 (112 in intervention arm) | CI | 34.25 | 45 | Remote Communication Methods | Successful Issuement of Prescription | Effects of prescribing clinic (psychiatric vs non-psychiatric, age, sex, and income) |
| Carvalho et al., (2023) (121) | 73 | CI | 35 | 51.7 | Web/App-Based Cognitive Therapy / Psychoeducation | Medication Possession Rate | Knowledge of ADHD User Version Mobile Application Rating Scale ASRS |
| Dan et al., (2016) (122) | 3 | CS | 35.67 | 33.3 | Remote Symptom Tracking | % of Negative Samples of CO Intake | - |
| Håvik et al., (2019) (123) | 11 | CS | N/G | N/G | Chatbot | User Experiences Fallback Message Evaluation | User Experiences  Fallback message evaluation |
| Sankesara et al., (2025) (124) | 40 | OCS | 27.64 (6.11) | 25 | Remote Symptom Tracking | Smartphone Usage Data | Wearable Data  Psychometric Survey Data |
| Surman et al., (2022) (125) | 206 | OCS | 37.3 | 24.3 | Remote Symptom Tracking | System Usability Scale Study Engagement | - |
| Patrickson et al., (2024) (126) | 6 clinicians / practitioners 9 service consumers | OCS | N/G | 53.33 | Remote Symptom Tracking | Qualitative Analysis for Stakeholder Opinions | - |
| Ware et al., (2025) (127) | 23 | OCS | 21 (N/G) | 33.33 | Remote Symptom Tracking | Prediction of weekly ADHD symptom severity | F1 Score  Precision  Recall  Specificity  Accuracy |
| **Acronyms:** Adult ADHD Self Report Scale (ASRS), Carbon Monoxide (CO), Case Series (CS), Controlled Intervention (CI), Observational and Cross-Sectional (OCS) | | | | | | | |

**Table S10:** List of papers with key information extracted for papers classified as Promoting Good Health.

| **Study** | **Study Size** | **Study Design** | **Age** | **% Male** | **Type of Technology** | **Primary Outcomes** | **Secondary Outcomes** |
| --- | --- | --- | --- | --- | --- | --- | --- |
| Jang et al., (2021) (128) | 46 | CI | 24.79 (7.21) | 43 | Chatbot | Usability Acceptability Side-Effects | CAARS QUIDS Self-rating Anxiety Scale PSS |
| Lindstedt et al., (2013) (129) | 19 | PPNC | 31.7 (8.58) | 36.84 | Cognitive Assistive Technologies | QUEST Individual Support Protocol Satisfaction of Daily Occupants MANSA | - |
| Luiu et al., (2018) (130) | 6 | CS | 20-55 (Range) | N/G | Web/App-Based Cognitive Therapy / Psychoeducation | User Satisfaction Perceived Helpfulness | - |
| Seery et al., (2025) (131) | 14 | OCS | 40.29 (11.14) | 35.71 | Web/App-Based Cognitive Therapy / Psychoeducation | Qualitative Analysis of Acceptability and User Engagement | App Use Analytical Metrics |
| Store et al., (2023) (132) | 6 | PPNC | Range (20-60) | 33.33 | Robotic Companion | Sleep Onset Latency Wake After Sleep Onset Total Sleep Time Sleep Efficiency Wrist Actigraphy for Sleep Measures ISI Pre-Sleep Arousal Scale HADS ASRS | Thematic Analysis of Semi-Structured Interviews |
| **Acronyms**: Adult ADHD Self-Report Scale (ASRS), Case Series (CS), Conners Adult ADHD Rating Scale (CAARS), Controlled Intervention (CI), Hospital Anxiety and Depression Scale (HADS), Insomnia Severity Index (ISI), Manchester Short Assessment of Quality of Life (MANSA), Observational and Cross-Sectional (OCS), Perceived Stress Scale (PSS), Pre-Post with No Control (PPNC), Quebec User Evaluation with Assistive Technology (QUEST), Quick Inventory of Depressive Symptomology (QUIDS) | | | | | | | |

**Table S11:** List of papers with key information extracted for papers classified as Communicate about Health and Care.

| **Study** | **Study Size** | **Study Design** | **Age** | **% Male** | **Type of Technology** | **Primary Outcomes** | **Secondary Outcomes** |
| --- | --- | --- | --- | --- | --- | --- | --- |
| Adamou et al., (2021) (133) | 117 (20 ASD + 93 ADHD) | Case Series | Range 15-70 | 66.7 | Remote Communication Methods | Telehealth Usability Questionnaire | None |
| **Acronyms:** Autism Spectrum Disorder (ASD) | | | | | | | |

# Risk of Bias Analysis Supplementary Data

## NICE DHT Category: Treat a Specific Condition

Table S12: Risk of Bias Raw Scoring Criterion for Treat Specific Condition - Case Series

| **RoB Criterion** | **CD** | **NA** | **No** | **NR** | **Yes** | **Total** |
| --- | --- | --- | --- | --- | --- | --- |
| Was the study question or objective clearly stated? | 0 | 0 | 0 | 0 | 1 | 1 |
| Was the study population clearly and fully described, including a case definition? | 0 | 0 | 0 | 0 | 1 | 1 |
| Were the cases consecutive? | 0 | 0 | 1 | 0 | 0 | 1 |
| Were the subjects comparable? | 0 | 0 | 0 | 0 | 1 | 1 |
| Was the intervention clearly described? | 0 | 0 | 0 | 0 | 1 | 1 |
| Were the outcome measures clearly defined, valid, reliable, and implemented consistently across all study participants? | 0 | 0 | 0 | 0 | 1 | 1 |
| Was the length of follow-up adequate? | 0 | 0 | 0 | 0 | 1 | 1 |
| Were the statistical methods well-described? | 0 | 0 | 0 | 0 | 1 | 1 |
| Were the results well-described? | 0 | 0 | 1 | 0 | 0 | 1 |

Table S13: Risk of Bias Raw Scoring Criterion for Treat Specific Condition – Controlled Intervention

| **RoB Criterion** | **CD** | **NA** | **No** | **NR** | **Yes** | **Total** |
| --- | --- | --- | --- | --- | --- | --- |
| Was the study described as randomized, a randomized trial, a randomized clinical trial, or an RCT? | 0 | 0 | 4 | 0 | 34 | 38 |
| Was the method of randomization adequate (i.e., use of randomly generated assignment)? | 0 | 2 | 2 | 3 | 31 | 38 |
| Was the treatment allocation concealed (so that assignments could not be predicted)? | 1 | 1 | 0 | 2 | 34 | 38 |
| Were study participants and providers blinded to treatment group assignment? | 2 | 0 | 24 | 1 | 11 | 38 |
| Were the people assessing the outcomes blinded to the participants' group assignments? | 4 | 0 | 12 | 12 | 10 | 38 |
| Were the groups similar at baseline on important characteristics that could affect outcomes (e.g., demographics, risk factors, co-morbid conditions)? | 3 | 2 | 2 | 3 | 28 | 38 |
| Was the overall drop-out rate from the study at endpoint 20% or lower of the number allocated to treatment? | 0 | 0 | 11 | 0 | 27 | 38 |
| Was the differential drop-out rate (between treatment groups) at endpoint 15 percentage points or lower? | 2 | 5 | 10 | 1 | 20 | 38 |
| Was there high adherence to the intervention protocols for each treatment group? | 3 | 0 | 10 | 13 | 12 | 38 |
| Were other interventions avoided or similar in the groups (e.g., similar background treatments)? | 1 | 0 | 8 | 13 | 16 | 38 |
| Were outcomes assessed using valid and reliable measures, implemented consistently across all study participants? | 0 | 0 | 2 | 0 | 36 | 38 |
| Did the authors report that the sample size was sufficiently large to be able to detect a difference in the main outcome between groups with at least 80% power? | 1 | 0 | 24 | 0 | 13 | 38 |
| Were outcomes reported or subgroups analyzed prespecified (i.e., identified before analyses were conducted)? | 0 | 0 | 13 | 0 | 25 | 38 |
| Were all randomized participants analyzed in the group to which they were originally assigned, i.e., did they use an intention-to-treat analysis? | 0 | 0 | 21 | 0 | 17 | 38 |

Table S14: Risk of Bias Raw Scoring Criterion for Treat Specific Condition – Observational and Cross-Sectional

| **RoB Criterion** | **CD** | **NA** | **No** | **NR** | **Yes** | **Total** |
| --- | --- | --- | --- | --- | --- | --- |
| Was the research question or objective in this paper clearly stated? | 0 | 0 | 0 | 0 | 7 | 7 |
| Was the study population clearly specified and defined? | 0 | 0 | 1 | 0 | 6 | 7 |
| Was the participation rate of eligible persons at least 50%? | 0 | 1 | 0 | 0 | 6 | 7 |
| Were all the subjects selected or recruited from the same or similar populations (including the same time period)? Were inclusion and exclusion criteria for being in the study prespecified and applied uniformly to all participants? | 0 | 0 | 0 | 0 | 7 | 7 |
| Was a sample size justification, power description, or variance and effect estimates provided? | 0 | 3 | 3 | 0 | 1 | 7 |
| For the analyses in this paper, were the exposure(s) of interest measured prior to the outcome(s) being measured? | 0 | 0 | 3 | 0 | 4 | 7 |
| Was the timeframe sufficient so that one could reasonably expect to see an association between exposure and outcome if it existed? | 0 | 2 | 0 | 0 | 5 | 7 |
| For exposures that can vary in amount or level, did the study examine different levels of the exposure as related to the outcome (e.g., categories of exposure, or exposure measured as continuous variable)? | 0 | 3 | 2 | 0 | 2 | 7 |
| Were the exposure measures (independent variables) clearly defined, valid, reliable, and implemented consistently across all study participants? | 0 | 0 | 2 | 0 | 5 | 7 |
| Was the exposure(s) assessed more than once over time? | 0 | 2 | 4 | 0 | 1 | 7 |
| Were the outcome measures (dependent variables) clearly defined, valid, reliable, and implemented consistently across all study participants? | 0 | 0 | 2 | 0 | 5 | 7 |
| Were the outcome assessors blinded to the exposure status of participants? | 0 | 1 | 4 | 1 | 1 | 7 |
| Was loss to follow-up after baseline 20% or less? | 0 | 1 | 1 | 0 | 5 | 7 |
| Were key potential confounding variables measured and adjusted statistically for their impact on the relationship between exposure(s) and outcome(s)? | 0 | 4 | 1 | 0 | 2 | 7 |

Table S15: Risk of Bias Raw Scoring Criterion for Treat Specific Condition – Pre-Post with No Control

| **RoB Criterion** | **CD** | **NA** | **No** | **NR** | **Yes** | **Total** |
| --- | --- | --- | --- | --- | --- | --- |
| Was the study question or objective clearly stated? | 0 | 0 | 0 | 0 | 17 | 17 |
| Were eligibility/selection criteria for the study population prespecified and clearly described? | 0 | 0 | 3 | 0 | 14 | 17 |
| Were the participants in the study representative of those who would be eligible for the test/service/intervention in the general or clinical population of interest? | 1 | 0 | 4 | 1 | 11 | 17 |
| Were all eligible participants that met the prespecified entry criteria enrolled? | 1 | 0 | 1 | 2 | 13 | 17 |
| Was the sample size sufficiently large to provide confidence in the findings? | 0 | 0 | 12 | 0 | 5 | 17 |
| Was the test/service/intervention clearly described and delivered consistently across the study population? | 1 | 0 | 1 | 1 | 14 | 17 |
| Were the outcome measures prespecified, clearly defined, valid, reliable, and assessed consistently across all study participants? | 0 | 2 | 3 | 0 | 12 | 17 |
| Were the people assessing the outcomes blinded to the participants' exposures/interventions? | 0 | 4 | 8 | 5 | 0 | 17 |
| Was the loss to follow-up after baseline 20% or less? Were those lost to follow-up accounted for in the analysis? | 0 | 0 | 6 | 1 | 10 | 17 |
| Did the statistical methods examine changes in outcome measures from before to after the intervention? Were statistical tests done that provided p values for the pre-to-post changes? | 0 | 1 | 1 | 0 | 15 | 17 |
| Were outcome measures of interest taken multiple times before the intervention and multiple times after the intervention (i.e., did they use an interrupted time-series design)? | 0 | 1 | 16 | 0 | 0 | 17 |
| If the intervention was conducted at a group level (e.g., a whole hospital, a community, etc.) did the statistical analysis take into account the use of individual-level data to determine effects at the group level? | 0 | 9 | 0 | 0 | 8 | 17 |

## NICE DHT Category: Drive Clinical Management

Table S16: Risk of Bias Raw Scoring Criterion for Drive Clinical Management – Case Series

| **RoB Criterion** | **CD** | **NA** | **No** | **NR** | **Yes** | **Total** |
| --- | --- | --- | --- | --- | --- | --- |
| Was the study question or objective clearly stated? | 0 | 0 | 0 | 0 | 1 | 1 |
| Was the study population clearly and fully described, including a case definition? | 0 | 0 | 0 | 0 | 1 | 1 |
| Were the cases consecutive? | 0 | 0 | 0 | 0 | 1 | 1 |
| Were the subjects comparable? | 0 | 0 | 0 | 1 | 0 | 1 |
| Was the intervention clearly described? | 0 | 0 | 1 | 0 | 0 | 1 |
| Were the outcome measures clearly defined, valid, reliable, and implemented consistently across all study participants? | 0 | 0 | 0 | 0 | 1 | 1 |
| Was the length of follow-up adequate? | 0 | 0 | 1 | 0 | 0 | 1 |
| Were the statistical methods well-described? | 0 | 0 | 1 | 0 | 0 | 1 |
| Were the results well-described? | 0 | 0 | 1 | 0 | 0 | 1 |

Table S17: Risk of Bias Raw Scoring Criterion for Drive Clinical Management – Observational and Cross-Sectional

| **RoB Criterion** | **CD** | **NA** | **No** | **NR** | **Yes** | **Total** |
| --- | --- | --- | --- | --- | --- | --- |
| Was the research question or objective in this paper clearly stated? | 0 | 0 | 2 | 0 | 32 | 34 |
| Was the study population clearly specified and defined? | 0 | 0 | 4 | 0 | 30 | 34 |
| Was the participation rate of eligible persons at least 50%? | 3 | 0 | 3 | 4 | 24 | 34 |
| Were all the subjects selected or recruited from the same or similar populations (including the same time period)? Were inclusion and exclusion criteria for being in the study prespecified and applied uniformly to all participants? | 3 | 0 | 4 | 1 | 26 | 34 |
| Was a sample size justification, power description, or variance and effect estimates provided? | 0 | 0 | 31 | 0 | 3 | 34 |
| For the analyses in this paper, were the exposure(s) of interest measured prior to the outcome(s) being measured? | 2 | 1 | 10 | 0 | 21 | 34 |
| Was the timeframe sufficient so that one could reasonably expect to see an association between exposure and outcome if it existed? | 0 | 1 | 0 | 0 | 33 | 34 |
| For exposures that can vary in amount or level, did the study examine different levels of the exposure as related to the outcome (e.g., categories of exposure, or exposure measured as continuous variable)? | 0 | 1 | 13 | 0 | 20 | 34 |
| Were the exposure measures (independent variables) clearly defined, valid, reliable, and implemented consistently across all study participants? | 2 | 0 | 14 | 0 | 18 | 34 |
| Was the exposure(s) assessed more than once over time? | 0 | 0 | 30 | 0 | 4 | 34 |
| Were the outcome measures (dependent variables) clearly defined, valid, reliable, and implemented consistently across all study participants? | 0 | 0 | 9 | 0 | 25 | 34 |
| Were the outcome assessors blinded to the exposure status of participants? | 0 | 1 | 8 | 23 | 2 | 34 |
| Was loss to follow-up after baseline 20% or less? | 2 | 0 | 2 | 4 | 26 | 34 |
| Were key potential confounding variables measured and adjusted statistically for their impact on the relationship between exposure(s) and outcome(s)? | 1 | 0 | 26 | 1 | 6 | 34 |

Table S18: Risk of Bias Raw Scoring Criterion for Drive Clinical Management – Pre-Post with No Control

| **RoB Criterion** | **CD** | **NA** | **No** | **NR** | **Yes** | **Total** |
| --- | --- | --- | --- | --- | --- | --- |
| Was the study question or objective clearly stated? | 0 | 0 | 0 | 0 | 1 | 1 |
| Were eligibility/selection criteria for the study population prespecified and clearly described? | 0 | 0 | 0 | 0 | 1 | 1 |
| Were the participants in the study representative of those who would be eligible for the test/service/intervention in the general or clinical population of interest? | 0 | 0 | 0 | 0 | 1 | 1 |
| Were all eligible participants that met the prespecified entry criteria enrolled? | 0 | 0 | 0 | 0 | 1 | 1 |
| Was the sample size sufficiently large to provide confidence in the findings? | 0 | 0 | 1 | 0 | 0 | 1 |
| Was the test/service/intervention clearly described and delivered consistently across the study population? | 0 | 0 | 0 | 0 | 1 | 1 |
| Were the outcome measures prespecified, clearly defined, valid, reliable, and assessed consistently across all study participants? | 0 | 0 | 0 | 0 | 1 | 1 |
| Were the people assessing the outcomes blinded to the participants' exposures/interventions? | 0 | 0 | 1 | 0 | 0 | 1 |
| Was the loss to follow-up after baseline 20% or less? Were those lost to follow-up accounted for in the analysis? | 0 | 0 | 1 | 0 | 0 | 1 |
| Did the statistical methods examine changes in outcome measures from before to after the intervention? Were statistical tests done that provided p values for the pre-to-post changes? | 0 | 0 | 0 | 0 | 1 | 1 |
| Were outcome measures of interest taken multiple times before the intervention and multiple times after the intervention (i.e., did they use an interrupted time-series design)? | 0 | 0 | 1 | 0 | 0 | 1 |
| If the intervention was conducted at a group level (e.g., a whole hospital, a community, etc.) did the statistical analysis take into account the use of individual-level data to determine effects at the group level? | 0 | 1 | 0 | 0 | 0 | 1 |

## NICE DHT Category: Diagnose a Specific Condition

Table S19: Risk of Bias Raw Scoring Criterion for Diagnose a Specific Condition – Controlled Intervention

| **RoB Criterion** | **CD** | **NA** | **No** | **NR** | **Yes** | **Total** |
| --- | --- | --- | --- | --- | --- | --- |
| Was the study described as randomized, a randomized trial, a randomized clinical trial, or an RCT? | 0 | 0 | 1 | 0 | 0 | 1 |
| Was the method of randomization adequate (i.e., use of randomly generated assignment)? | 0 | 0 | 0 | 0 | 1 | 1 |
| Was the treatment allocation concealed (so that assignments could not be predicted)? | 0 | 0 | 0 | 1 | 0 | 1 |
| Were study participants and providers blinded to treatment group assignment? | 0 | 0 | 0 | 1 | 0 | 1 |
| Were the people assessing the outcomes blinded to the participants' group assignments? | 0 | 0 | 0 | 1 | 0 | 1 |
| Were the groups similar at baseline on important characteristics that could affect outcomes (e.g., demographics, risk factors, co-morbid conditions)? | 0 | 0 | 0 | 1 | 0 | 1 |
| Was the overall drop-out rate from the study at endpoint 20% or lower of the number allocated to treatment? | 0 | 0 | 0 | 0 | 1 | 1 |
| Was the differential drop-out rate (between treatment groups) at endpoint 15 percentage points or lower? | 0 | 0 | 1 | 0 | 0 | 1 |
| Was there high adherence to the intervention protocols for each treatment group? | 0 | 0 | 0 | 1 | 0 | 1 |
| Were other interventions avoided or similar in the groups (e.g., similar background treatments)? | 0 | 0 | 0 | 1 | 0 | 1 |
| Were outcomes assessed using valid and reliable measures, implemented consistently across all study participants? | 0 | 1 | 0 | 0 | 0 | 1 |
| Did the authors report that the sample size was sufficiently large to be able to detect a difference in the main outcome between groups with at least 80% power? | 0 | 0 | 1 | 0 | 0 | 1 |
| Were outcomes reported or subgroups analyzed prespecified (i.e., identified before analyses were conducted)? | 0 | 0 | 1 | 0 | 0 | 1 |
| Were all randomized participants analyzed in the group to which they were originally assigned, i.e., did they use an intention-to-treat analysis? | 0 | 0 | 1 | 0 | 0 | 1 |

Table S20: Risk of Bias Raw Scoring Criterion for Diagnose a Specific Condition – Observational and Cross-Sectional

| **RoB Criterion** | **CD** | **NA** | **No** | **NR** | **Yes** | **Total** |
| --- | --- | --- | --- | --- | --- | --- |
| Was the research question or objective in this paper clearly stated? | 0 | 0 | 4 | 0 | 14 | 18 |
| Was the study population clearly specified and defined? | 0 | 0 | 9 | 0 | 9 | 18 |
| Was the participation rate of eligible persons at least 50%? | 2 | 1 | 0 | 6 | 9 | 18 |
| Were all the subjects selected or recruited from the same or similar populations (including the same time period)? Were inclusion and exclusion criteria for being in the study prespecified and applied uniformly to all participants? | 2 | 0 | 3 | 6 | 7 | 18 |
| Was a sample size justification, power description, or variance and effect estimates provided? | 0 | 0 | 18 | 0 | 0 | 18 |
| For the analyses in this paper, were the exposure(s) of interest measured prior to the outcome(s) being measured? | 2 | 0 | 6 | 1 | 9 | 18 |
| Was the timeframe sufficient so that one could reasonably expect to see an association between exposure and outcome if it existed? | 0 | 0 | 0 | 0 | 18 | 18 |
| For exposures that can vary in amount or level, did the study examine different levels of the exposure as related to the outcome (e.g., categories of exposure, or exposure measured as continuous variable)? | 1 | 0 | 11 | 0 | 6 | 18 |
| Were the exposure measures (independent variables) clearly defined, valid, reliable, and implemented consistently across all study participants? | 1 | 0 | 14 | 0 | 3 | 18 |
| Was the exposure(s) assessed more than once over time? | 1 | 0 | 15 | 1 | 1 | 18 |
| Were the outcome measures (dependent variables) clearly defined, valid, reliable, and implemented consistently across all study participants? | 0 | 0 | 5 | 0 | 13 | 18 |
| Were the outcome assessors blinded to the exposure status of participants? | 0 | 0 | 5 | 13 | 0 | 18 |
| Was loss to follow-up after baseline 20% or less? | 2 | 1 | 1 | 3 | 11 | 18 |
| Were key potential confounding variables measured and adjusted statistically for their impact on the relationship between exposure(s) and outcome(s)? | 2 | 1 | 14 | 0 | 1 | 18 |

## NICE DHT Category: Inform Clinical Management

Table S21: Risk of Bias Raw Scoring Criterion for Inform Clinical Management – Case Series

| **RoB Criterion** | **CD** | **NA** | **No** | **NR** | **Yes** | **Total** |
| --- | --- | --- | --- | --- | --- | --- |
| Was the study question or objective clearly stated? | 0 | 0 | 0 | 0 | 2 | 2 |
| Was the study population clearly and fully described, including a case definition? | 0 | 0 | 1 | 0 | 1 | 2 |
| Were the cases consecutive? | 2 | 0 | 0 | 0 | 0 | 2 |
| Were the subjects comparable? | 1 | 0 | 1 | 0 | 0 | 2 |
| Was the intervention clearly described? | 0 | 0 | 0 | 0 | 2 | 2 |
| Were the outcome measures clearly defined, valid, reliable, and implemented consistently across all study participants? | 0 | 0 | 1 | 0 | 1 | 2 |
| Was the length of follow-up adequate? | 0 | 1 | 1 | 0 | 0 | 2 |
| Were the statistical methods well-described? | 0 | 0 | 1 | 0 | 1 | 2 |
| Were the results well-described? | 0 | 0 | 1 | 0 | 1 | 2 |

Table S22: Risk of Bias Raw Scoring Criterion for Inform Clinical Management – Controlled Intervention

| **RoB Criterion** | **CD** | **NA** | **No** | **NR** | **Yes** | **Total** |
| --- | --- | --- | --- | --- | --- | --- |
| Was the study described as randomized, a randomized trial, a randomized clinical trial, or an RCT? | 0 | 0 | 1 | 0 | 1 | 2 |
| Was the method of randomization adequate (i.e., use of randomly generated assignment)? | 1 | 1 | 0 | 0 | 0 | 2 |
| Was the treatment allocation concealed (so that assignments could not be predicted)? | 0 | 1 | 0 | 0 | 1 | 2 |
| Were study participants and providers blinded to treatment group assignment? | 0 | 0 | 2 | 0 | 0 | 2 |
| Were the people assessing the outcomes blinded to the participants' group assignments? | 0 | 0 | 1 | 1 | 0 | 2 |
| Were the groups similar at baseline on important characteristics that could affect outcomes (e.g., demographics, risk factors, co-morbid conditions)? | 0 | 0 | 0 | 0 | 2 | 2 |
| Was the overall drop-out rate from the study at endpoint 20% or lower of the number allocated to treatment? | 0 | 0 | 0 | 0 | 2 | 2 |
| Was the differential drop-out rate (between treatment groups) at endpoint 15 percentage points or lower? | 0 | 0 | 0 | 0 | 2 | 2 |
| Was there high adherence to the intervention protocols for each treatment group? | 0 | 0 | 0 | 1 | 1 | 2 |
| Were other interventions avoided or similar in the groups (e.g., similar background treatments)? | 0 | 0 | 0 | 1 | 1 | 2 |
| Were outcomes assessed using valid and reliable measures, implemented consistently across all study participants? | 0 | 0 | 0 | 0 | 2 | 2 |
| Did the authors report that the sample size was sufficiently large to be able to detect a difference in the main outcome between groups with at least 80% power? | 0 | 0 | 2 | 0 | 0 | 2 |
| Were outcomes reported or subgroups analyzed prespecified (i.e., identified before analyses were conducted)? | 0 | 0 | 1 | 0 | 1 | 2 |
| Were all randomized participants analyzed in the group to which they were originally assigned, i.e., did they use an intention-to-treat analysis? | 0 | 0 | 1 | 0 | 1 | 2 |

Table S23: Risk of Bias Raw Scoring Criterion for Inform Clinical Management – Observational and Cross-Sectional

| **RoB Criterion** | **CD** | **NA** | **No** | **NR** | **Yes** | **Total** |
| --- | --- | --- | --- | --- | --- | --- |
| Was the research question or objective in this paper clearly stated? | 0 | 0 | 0 | 0 | 5 | 5 |
| Was the study population clearly specified and defined? | 0 | 0 | 0 | 0 | 5 | 5 |
| Was the participation rate of eligible persons at least 50%? | 0 | 0 | 0 | 0 | 5 | 5 |
| Were all the subjects selected or recruited from the same or similar populations (including the same time period)? Were inclusion and exclusion criteria for being in the study prespecified and applied uniformly to all participants? | 0 | 0 | 0 | 0 | 5 | 5 |
| Was a sample size justification, power description, or variance and effect estimates provided? | 0 | 2 | 3 | 0 | 0 | 5 |
| For the analyses in this paper, were the exposure(s) of interest measured prior to the outcome(s) being measured? | 0 | 0 | 3 | 0 | 2 | 5 |
| Was the timeframe sufficient so that one could reasonably expect to see an association between exposure and outcome if it existed? | 0 | 1 | 0 | 0 | 4 | 5 |
| For exposures that can vary in amount or level, did the study examine different levels of the exposure as related to the outcome (e.g., categories of exposure, or exposure measured as continuous variable)? | 0 | 0 | 1 | 0 | 4 | 5 |
| Were the exposure measures (independent variables) clearly defined, valid, reliable, and implemented consistently across all study participants? | 0 | 0 | 0 | 0 | 5 | 5 |
| Was the exposure(s) assessed more than once over time? | 0 | 1 | 2 | 0 | 2 | 5 |
| Were the outcome measures (dependent variables) clearly defined, valid, reliable, and implemented consistently across all study participants? | 0 | 0 | 0 | 0 | 5 | 5 |
| Were the outcome assessors blinded to the exposure status of participants? | 0 | 1 | 2 | 2 | 0 | 5 |
| Was loss to follow-up after baseline 20% or less? | 0 | 0 | 2 | 0 | 3 | 5 |
| Were key potential confounding variables measured and adjusted statistically for their impact on the relationship between exposure(s) and outcome(s)? | 0 | 1 | 4 | 0 | 0 | 5 |

## NICE DHT Category: Promoting Good Health

Table S24: Risk of Bias Raw Scoring Criterion for Promoting Good Health – Case Series

| **RoB Criterion** | **CD** | **NA** | **No** | **NR** | **Yes** | **Total** |
| --- | --- | --- | --- | --- | --- | --- |
| Was the study question or objective clearly stated? | 0 | 0 | 0 | 0 | 1 | 1 |
| Was the study population clearly and fully described, including a case definition? | 0 | 0 | 1 | 0 | 0 | 1 |
| Were the cases consecutive? | 0 | 0 | 0 | 1 | 0 | 1 |
| Were the subjects comparable? | 0 | 0 | 0 | 1 | 0 | 1 |
| Was the intervention clearly described? | 0 | 0 | 1 | 0 | 0 | 1 |
| Were the outcome measures clearly defined, valid, reliable, and implemented consistently across all study participants? | 0 | 0 | 0 | 0 | 1 | 1 |
| Was the length of follow-up adequate? | 0 | 1 | 0 | 0 | 0 | 1 |
| Were the statistical methods well-described? | 0 | 1 | 0 | 0 | 0 | 1 |
| Were the results well-described? | 0 | 0 | 1 | 0 | 0 | 1 |

Table S25: Risk of Bias Raw Scoring Criterion for Promoting Good Health – Controlled Intervention

| **RoB Criterion** | **CD** | **NA** | **No** | **NR** | **Yes** | **Total** |
| --- | --- | --- | --- | --- | --- | --- |
| Was the study described as randomized, a randomized trial, a randomized clinical trial, or an RCT? | 0 | 0 | 0 | 0 | 1 | 1 |
| Was the method of randomization adequate (i.e., use of randomly generated assignment)? | 0 | 0 | 0 | 0 | 1 | 1 |
| Was the treatment allocation concealed (so that assignments could not be predicted)? | 0 | 0 | 0 | 1 | 0 | 1 |
| Were study participants and providers blinded to treatment group assignment? | 0 | 0 | 1 | 0 | 0 | 1 |
| Were the people assessing the outcomes blinded to the participants' group assignments? | 0 | 0 | 1 | 0 | 0 | 1 |
| Were the groups similar at baseline on important characteristics that could affect outcomes (e.g., demographics, risk factors, co-morbid conditions)? | 0 | 0 | 0 | 0 | 1 | 1 |
| Was the overall drop-out rate from the study at endpoint 20% or lower of the number allocated to treatment? | 0 | 0 | 0 | 0 | 1 | 1 |
| Was the differential drop-out rate (between treatment groups) at endpoint 15 percentage points or lower? | 0 | 0 | 0 | 0 | 1 | 1 |
| Was there high adherence to the intervention protocols for each treatment group? | 0 | 0 | 0 | 0 | 1 | 1 |
| Were other interventions avoided or similar in the groups (e.g., similar background treatments)? | 0 | 0 | 0 | 0 | 1 | 1 |
| Were outcomes assessed using valid and reliable measures, implemented consistently across all study participants? | 0 | 0 | 0 | 0 | 1 | 1 |
| Did the authors report that the sample size was sufficiently large to be able to detect a difference in the main outcome between groups with at least 80% power? | 0 | 0 | 1 | 0 | 0 | 1 |
| Were outcomes reported or subgroups analyzed prespecified (i.e., identified before analyses were conducted)? | 0 | 0 | 1 | 0 | 0 | 1 |
| Were all randomized participants analyzed in the group to which they were originally assigned, i.e., did they use an intention-to-treat analysis? | 0 | 0 | 0 | 0 | 1 | 1 |

Table S26: Risk of Bias Raw Scoring Criterion for Promoting Good Health – Observational and Cross-Sectional

| **RoB Criterion** | **CD** | **NA** | **No** | **NR** | **Yes** | **Total** |
| --- | --- | --- | --- | --- | --- | --- |
| Was the research question or objective in this paper clearly stated? | 0 | 0 | 0 | 0 | 1 | 1 |
| Was the study population clearly specified and defined? | 0 | 0 | 0 | 0 | 1 | 1 |
| Was the participation rate of eligible persons at least 50%? | 0 | 0 | 0 | 0 | 1 | 1 |
| Were all the subjects selected or recruited from the same or similar populations (including the same time period)? Were inclusion and exclusion criteria for being in the study prespecified and applied uniformly to all participants? | 0 | 0 | 0 | 0 | 1 | 1 |
| Was a sample size justification, power description, or variance and effect estimates provided? | 0 | 0 | 1 | 0 | 0 | 1 |
| For the analyses in this paper, were the exposure(s) of interest measured prior to the outcome(s) being measured? | 0 | 1 | 0 | 0 | 0 | 1 |
| Was the timeframe sufficient so that one could reasonably expect to see an association between exposure and outcome if it existed? | 0 | 0 | 0 | 0 | 1 | 1 |
| For exposures that can vary in amount or level, did the study examine different levels of the exposure as related to the outcome (e.g., categories of exposure, or exposure measured as continuous variable)? | 0 | 1 | 0 | 0 | 0 | 1 |
| Were the exposure measures (independent variables) clearly defined, valid, reliable, and implemented consistently across all study participants? | 0 | 1 | 0 | 0 | 0 | 1 |
| Was the exposure(s) assessed more than once over time? | 0 | 1 | 0 | 0 | 0 | 1 |
| Were the outcome measures (dependent variables) clearly defined, valid, reliable, and implemented consistently across all study participants? | 0 | 0 | 0 | 0 | 1 | 1 |
| Were the outcome assessors blinded to the exposure status of participants? | 0 | 1 | 0 | 0 | 0 | 1 |
| Was loss to follow-up after baseline 20% or less? | 0 | 0 | 0 | 0 | 1 | 1 |
| Were key potential confounding variables measured and adjusted statistically for their impact on the relationship between exposure(s) and outcome(s)? | 0 | 1 | 0 | 0 | 0 | 1 |

Table S27: Risk of Bias Raw Scoring Criterion for Promoting Good Health – Pre-Post with No Control

| **RoB Criterion** | **CD** | **NA** | **No** | **NR** | **Yes** | **Total** |
| --- | --- | --- | --- | --- | --- | --- |
| Was the study question or objective clearly stated? | 0 | 0 | 0 | 0 | 2 | 2 |
| Were eligibility/selection criteria for the study population prespecified and clearly described? | 0 | 0 | 0 | 0 | 2 | 2 |
| Were the participants in the study representative of those who would be eligible for the test/service/intervention in the general or clinical population of interest? | 0 | 0 | 0 | 0 | 2 | 2 |
| Were all eligible participants that met the prespecified entry criteria enrolled? | 0 | 0 | 0 | 0 | 2 | 2 |
| Was the sample size sufficiently large to provide confidence in the findings? | 0 | 0 | 2 | 0 | 0 | 2 |
| Was the test/service/intervention clearly described and delivered consistently across the study population? | 0 | 0 | 0 | 0 | 2 | 2 |
| Were the outcome measures prespecified, clearly defined, valid, reliable, and assessed consistently across all study participants? | 0 | 0 | 0 | 0 | 2 | 2 |
| Were the people assessing the outcomes blinded to the participants' exposures/interventions? | 0 | 0 | 2 | 0 | 0 | 2 |
| Was the loss to follow-up after baseline 20% or less? Were those lost to follow-up accounted for in the analysis? | 0 | 0 | 0 | 0 | 2 | 2 |
| Did the statistical methods examine changes in outcome measures from before to after the intervention? Were statistical tests done that provided p values for the pre-to-post changes? | 0 | 0 | 1 | 0 | 1 | 2 |
| Were outcome measures of interest taken multiple times before the intervention and multiple times after the intervention (i.e., did they use an interrupted time-series design)? | 0 | 0 | 2 | 0 | 0 | 2 |
| If the intervention was conducted at a group level (e.g., a whole hospital, a community, etc.) did the statistical analysis take into account the use of individual-level data to determine effects at the group level? | 0 | 2 | 0 | 0 | 0 | 2 |

## NICE DHT Category: Communicate about Health and Care

Table S28: Risk of Bias Raw Scoring Criterion for Communicate about Health and Care – Case Series

| **RoB Criterion** | **CD** | **NA** | **No** | **NR** | **Yes** | **Total** |
| --- | --- | --- | --- | --- | --- | --- |
| Was the study question or objective clearly stated? | 0 | 0 | 0 | 0 | 1 | 1 |
| Was the study population clearly and fully described, including a case definition? | 0 | 0 | 0 | 0 | 1 | 1 |
| Were the cases consecutive? | 0 | 0 | 0 | 0 | 1 | 1 |
| Were the subjects comparable? | 0 | 0 | 0 | 0 | 1 | 1 |
| Was the intervention clearly described? | 0 | 0 | 0 | 0 | 1 | 1 |
| Were the outcome measures clearly defined, valid, reliable, and implemented consistently across all study participants? | 0 | 0 | 0 | 0 | 1 | 1 |
| Was the length of follow-up adequate? | 0 | 0 | 1 | 0 | 0 | 1 |
| Were the statistical methods well-described? | 0 | 0 | 0 | 0 | 1 | 1 |
| Were the results well-described? | 0 | 0 | 0 | 0 | 1 | 1 |

# References

1. Ahlers J, Baumgartner C, Augsburger M, Wenger A, Malischnig D, Boumparis N, et al. Cannabis Use in Adults Who Screen Positive for Attention Deficit/Hyperactivity Disorder: Canreduce 2.0 Randomized Controlled Trial Subgroup Analysis. *Journal of medical Internet research* (2022) 24(4):e30138. doi: 10.2196/30138.

2. Antshel KM, McBride H, Knouse LE. Bridging the Gap: Digital Cbt for Adults Managing Adhd Challenges. *J Atten Disord* (2025). doi: 10.1177/10870547251384462.

3. Champ RE, Meneses RW, Adamou M, Gillibrand W, Arrey S, Tolchard B. A Neuroaffirmative, Self-Determination Theory-Based Psychosocial Intervention for Adults with Attention-Deficit/Hyperactivity Disorder: Randomized Feasibility Study. *JMIR Form Res* (2025) 9:e69943. Epub 20251029. doi: 10.2196/69943.

4. Flobak E, Nordby ES, Guribye F, Kenter R, Nordgreen T, Lundervold AJ. Designing Videos with and for Adults with Adhd for an Online Intervention: Participatory Design Study and Thematic Analysis of Evaluation. *Jmir Mental Health* (2021) 8(9). doi: 10.2196/30292.

5. Galili-Simhon S, Maeir A. Cognitive Functional Remote Group Intervention for Adults with Attention Deficit Hyperactivity Disorder: A Feasibility Study. *British Journal of Occupational Therapy* (2023) 86(10):686-96. doi: 10.1177/03080226231177842.

6. Kenter RMF, Gjestad R, Lundervold AJ, Nordgreen T. A Self-Guided Internet-Delivered Intervention for Adults with Adhd: Results from a Randomized Controlled Trial. *Internet Interv* (2023) 32. doi: 10.1016/j.invent.2023.100614.

7. Knouse LE, Hu X, Sachs G, Isaacs S. Usability and Feasibility of a Cognitive-Behavioral Mobile App for Adhd in Adults. *PLoS Digital Health* (2022) 1(8):1-19. doi: 10.1371/journal.pdig.0000083.

8. Moëll B, Kollberg L, Nasri B, Lindefors N, Kaldo V. Living Smart — a Randomized Controlled Trial of a Guided Online Course Teaching Adults with Adhd or Sub-Clinical Adhd to Use Smartphones to Structure Their Everyday Life. *Internet Interventions* (2015) 2(1):24-31. doi: <https://doi.org/10.1016/j.invent.2014.11.004>.

9. Nasri B, Cassel M, Enhärje J, Larsson M, Hirvikoski T, Ginsberg Y, et al. Internet Delivered Cognitive Behavioral Therapy for Adults with Adhd - a Randomized Controlled Trial. *Internet Interv* (2023) 33. doi: 10.1016/j.invent.2023.100636.

10. Nordby ES, Kenter RMF, Lundervold AJ, Nordgreen T. A Self-Guided Internet-Delivered Intervention for Adults with Adhd: A Feasibility Study. *Internet Interventions* (2021) 25:100416. doi: <https://doi.org/10.1016/j.invent.2021.100416>.

11. Nordby ES, Gjestad R, Kenter RMF, Guribye F, Mukhiya SK, Lundervold AJ, et al. The Effect of Sms Reminders on Adherence in a Self-Guided Internet-Delivered Intervention for Adults with Adhd. *Frontiers in Digital Health* (2022) 4. doi: 10.3389/fdgth.2022.821031.

12. Nordby ES, Guribye F, Schønning V, Andersen SL, Kuntsi J, Lundervold AJ. A Blended Intervention Targeting Emotion Dysregulation in Adults with Attention-Deficit/Hyperactivity Disorder: Development and Feasibility Study. *JMIR Formative Research* (2024) 8(1). doi: 10.2196/53931.

13. Nordby ES, Schonning V, Barnes A, Denyer H, Kuntsi J, Lundervold AJ, et al. Experiences of Change Following a Blended Intervention for Adults with Adhd and Emotion Dysregulation: A Qualitative Interview Study. *BMC Psychiatry* (2025) 25(1):56. doi: doi:10.1186/s12888-025-06476-1.

14. Oscarsson M, Hammarback S, Blom Wiberg K, Rozental A, Ginsberg Y, Carlbring P, et al. Web-Based Stress Management for Working Adults with Attention-Deficit/Hyperactivity Disorder (Adhd): Single-Arm, Open Pilot Trial. *JMIR formative research* (2025) 9:e66388. Epub 20250529. doi: 10.2196/66388.

15. Pettersson R, Söderström S, Edlund-Söderström K, Nilsson KW. Internet-Based Cognitive Behavioral Therapy for Adults with Adhd in Outpatient Psychiatric Care. *J Atten Disord* (2017) 21(6):508-21. doi: 10.1177/1087054714539998.

16. Scholl J, Kohls E, Görges F, Steinbrecher M, Baldofski S, Moessner M, et al. Acceptability and Feasibility of the Transfer of Face-to-Face Group Therapy to Online Group Chats in a Psychiatric Outpatient Setting during the Covid-19 Pandemic: Longitudinal Observational Study. *Jmir Formative Research* (2021) 5(7). doi: 10.2196/27865.

17. Seery C, Leonard-Curtin A, Naismith L, King N, Kilbride K, Wrigley M, et al. The Understanding and Managing Adult Adhd Programme: A Qualitative Evaluation of Online Psychoeducation with Acceptance and Commitment Therapy for Adults with Adhd. *Journal of Contextual Behavioral Science* (2023) 29:254-63. doi: <https://dx.doi.org/10.1016/j.jcbs.2023.08.005>.

18. Seery C, Leonard-Curtin A, Naismith L, King N, O'Donnell F, Byrne B, et al. Feasibility of the Understanding and Managing Adult Adhd Programme: Open-Access Online Group Psychoeducation and Acceptance and Commitment Therapy for Adults with Attention-Deficit Hyperactivity Disorder. *BJPsych Open* (2024) 10(5):e163. doi: doi:10.1192/bjo.2024.743.

19. Sehlin H, Ahlstrom BH, Andersson G, Wentz E. Experiences of an Internet-Based Support and Coaching Model for Adolescents and Young Adults with Adhd and Autism Spectrum Disorder-a Qualitative Study. *BMC Psychiatry* (2018) 18. doi: <https://dx.doi.org/10.1186/s12888-018-1599-9>.

20. Selaskowski B, Steffens M, Schulze M, Lingen M, Aslan B, Rosen H, et al. Smartphone-Assisted Psychoeducation in Adult Attention-Deficit/Hyperactivity Disorder: A Randomized Controlled Trial. *Psychiatry research* (2022) 317:114802. doi: 10.1016/j.psychres.2022.114802.

21. Selaskowski B, Reiland M, Schulze M, Aslan B, Kannen K, Wiebe A, et al. Chatbot-Supported Psychoeducation in Adult Attention-Deficit Hyperactivity Disorder: Randomised Controlled Trial. *BJPsych Open* (2023) 9. doi: <https://dx.doi.org/10.1192/bjo.2023.573>.

22. Shelton CR, Hartung CM, Canu WH. Feasibility and Acceptability of an Internet-Based Intervention for Young Adults with Adhd. *J Technol Behav Sci* (2022) 7(4):428-38. Epub 2022/05/24. doi: 10.1007/s41347-022-00256-4.

23. Tsirmpas C, Nikolakopoulou M, Kaplow S, Andrikopoulos D, Fatouros P, Kontoangelos K, et al. A Digital Mental Health Support Program for Depression and Anxiety in Populations with Attention-Deficit/Hyperactivity Disorder: Feasibility and Usability Study. *Jmir Formative Research* (2023) 7. doi: 10.2196/48362.

24. Ulusoy V, Bilican I, Gormez A. Effectiveness of an Online Dialectical Behavior Therapy Skills Training in Adults with Attention-Deficit/Hyperactivity Disorder: A Randomized Controlled Trial. *Psychotherapy Research* (2024):No-Specified. doi: <https://dx.doi.org/10.1080/10503307.2024.2311773>.

25. Wentz E, Nydén A, Krevers B. Development of an Internet-Based Support and Coaching Model for Adolescents and Young Adults with Adhd and Autism Spectrum Disorders: A Pilot Study. *Eur Child Adolesc Psychiatry* (2012) 21(11):611-22. Epub 2012/06/28. doi: 10.1007/s00787-012-0297-2.

26. Wyler H, Liebrenz M, Ajdacic-Gross V, Seifritz E, Young S, Burger P, et al. Treatment Provision for Adults with Adhd during the Covid-19 Pandemic: An Exploratory Study on Patient and Therapist Experience with on-Site Sessions Using Face Masks Vs. Telepsychiatric Sessions. *BMC Psychiatry* (2021) 21. doi: <https://dx.doi.org/10.1186/s12888-021-03236-9>.

27. Zhang SY, Pan MR, Zhang LQ, Li HM, Zhao MJ, Dong M, et al. Efficacy of Internet-Based Cognitive Behavioral Therapy for Medicated Adults with Attention-Deficit/Hyperactivity Disorder (Adhd): A Randomized Controlled Trial. *Psychiatry Res* (2025) 344:116352. doi: doi:10.1016/j.psychres.2025.116352.

28. Cunha F, Campos S, Simões-Silva V, Brugada-Ramentol V, Sá-Moura B, Jalali H, et al. The Effect of a Virtual Reality Based Intervention on Processing Speed and Working Memory in Individuals with Adhd—a Pilot-Study. *Frontiers in Virtual Reality* (2023) 4. doi: 10.3389/frvir.2023.1108060.

29. Kolodny T, Ashkenazi Y, Farhi M, Shalev L. Computerized Progressive Attention Training (Cpat) Vs. Active Control in Adults with Adhd. *Journal of Cognitive Enhancement* (2017) 1(4):526-38. doi: 10.1007/s41465-017-0056-x.

30. Lintas A, Bader M, Villa AEP. Boosting Working Memory in Adhd: Adaptive Dual N-Back Training Enhances Wais-Iv Performance, but Yields Mixed Corsi Outcomes. *Brain Sciences* (2025) 15(9). Epub 20250916. doi: 10.3390/brainsci15090998.

31. Marcelle ET, Ho EJ, Kaplan MS, Adler LA, Castellanos FX, Milham MP. Cogmed Working Memory Training Presents Unique Implementation Challenges in Adults with Adhd. *Front Psychiatry* (2018) 9. doi: <https://dx.doi.org/10.3389/fpsyt.2018.00388>.

32. Mawjee K, Woltering S, Lai N, Gotlieb H, Kronitz R, Tannock R. Working Memory Training in Adhd: Controlling for Engagement, Motivation, and Expectancy of Improvement (Pilot Study). *J Atten Disord* (2014) 21(11):956-68. doi: 10.1177/1087054714557356.

33. Mawjee K, Woltering S, Tannock R. Working Memory Training in Post-Secondary Students with Adhd: A Randomized Controlled Study. *PLoS One* (2015) 10(9):e0137173. Epub 2015/09/24. doi: 10.1371/journal.pone.0137173.

34. Osman AM, Madore KP, Jaffe PI, Offidani E, Childress AC, Newcorn JH, et al. Real-World Effectiveness of a Widely Available Digital Health Program in Adults Reporting a Lifetime Diagnosis of Adhd. *Npj Mental Health Research* (2025) 4(1):38. Epub 20250822. doi: 10.1038/s44184-025-00157-3.

35. Selaskowski B, Asché LM, Wiebe A, Kannen K, Aslan B, Gerding TM, et al. Gaze-Based Attention Refocusing Training in Virtual Reality for Adult Attention-Deficit/Hyperactivity Disorder. *BMC Psychiatry* (2023) 23(1):74. Epub 2023/01/27. doi: 10.1186/s12888-023-04551-z.

36. Sjöwall D, Berglund M, Hirvikoski T. Computerized Working Memory Training for Adults with Adhd in a Psychiatric Outpatient Context-a Feasibility Trial. *Applied Neuropsychology-Adult* (2023). doi: 10.1080/23279095.2022.2162900.

37. Stern A, Malik E, Pollak Y, Bonne O, Maeir A. The Efficacy of Computerized Cognitive Training in Adults with Adhd: A Randomized Controlled Trial. *J Atten Disord* (2016) 20(12):991-1003. Epub 2016/11/04. doi: 10.1177/1087054714529815.

38. Stern A, Pollack Y, Yakir A, Maeir A. A Pilot Study of Computerized Cognitive Training in Adults with Attention-Deficit/Hyperactivity Disorder: Change in Life Following 3 Months of Training Using the Atttgo[Tm] Program. *Israel Journal of Occupational Therapy / Ketab ’et Yiśreʼeliy Leriypẇy be ’Iysẇq* (2012) 21(4):E79-100.

39. Stern P, Kolodny T, Tsafrir S, Cohen G, Shalev L. Near and Far Transfer Effects of Computerized Progressive Attention Training (Cpat) Versus Mindfulness Based Stress Reduction (Mbsr) Practice among Adults with Adhd. *J Atten Disord* (2023) 27(7):757-76. Epub 2023/02/17. doi: 10.1177/10870547231155877.

40. Woltering S, Gu C, Liu Z-X, Tannock R. Visuospatial Working Memory Capacity in the Brain after Working Memory Training in College Students with Adhd: A Randomized Controlled Trial. *J Atten Disord* (2021) 25(7):1010-20. doi: <https://dx.doi.org/10.1177/1087054719879487>.

41. Cowley B, Holmstrom E, Juurmaa K, Kovarskis L, Krause CM. Computer Enabled Neuroplasticity Treatment: A Clinical Trial of a Novel Design for Neurofeedback Therapy in Adult Adhd. *Frontiers in Human Neuroscience* (2016) 10. doi: 10.3389/fnhum.2016.00205.

42. Esmaeilzadeh M, Soltanian-Zadeh H, Tabrizi YM, editors. Effect of Neurofeedback on Brain Functional Connectivity in Adult Adhd Patients: A Resting-State Fmri Study. *2021 28th National and 6th International Iranian Conference on Biomedical Engineering (ICBME)*; 2021 25-26 Nov. 2021.

43. Hudak J, Rosenbaum D, Barth B, Fallgatter AJ, Ehlis AC. Functionally Disconnected: A Look at How Study Design Influences Neurofeedback Data and Mechanisms in Attention-Deficit/ Hyperactivity Disorder. *PLoS ONE* (2018) 13(8). doi: 10.1371/journal.pone.0200931.

44. Mayer K, Wyckoff SN, Schulz U, Strehl U. Neurofeedback for Adult Attention-Deficit/Hyperactivity Disorder: Investigation of Slow Cortical Potential Neurofeedback—Preliminary Results. *Journal of Neurotherapy* (2012) 16(1):37-45. doi: 10.1080/10874208.2012.650113.

45. Ochi Y, Laksanasopin T, Kaewkamnerdpong B, Thanasuan K, editors. Neurofeedback Game for Attention Training in Adults. *2017 10th Biomedical Engineering International Conference (BMEiCON)*; 2017 31 Aug.-2 Sept. 2017.

46. Schönenberg M, Wiedemann E, Schneidt A, Scheeff J, Logemann A, Keune PM, et al. Neurofeedback, Sham Neurofeedback, and Cognitive-Behavioural Group Therapy in Adults with Attention-Deficit Hyperactivity Disorder: A Triple-Blind, Randomised, Controlled Trial. *Lancet Psychiatry* (2017) 4(9):673-84. Epub 2017/08/15. doi: 10.1016/s2215-0366(17)30291-2.

47. Veilahti AVP, Kovarskis L, Cowley BU. Neurofeedback Learning Is Skill Acquisition but Does Not Guarantee Treatment Benefit: Continuous-Time Analysis of Learning-Curves from a Clinical Trial for Adhd. *Front Hum Neurosci* (2021) 15:668780. Epub 2021/07/20. doi: 10.3389/fnhum.2021.668780.

48. Whitehead JC, Neeman R, Doniger GM. Preliminary Real-World Evidence Supporting the Efficacy of a Remote Neurofeedback System in Improving Mental Health: Retrospective Single-Group Pretest-Posttest Study. *Jmir Formative Research* (2022) 6(7). doi: 10.2196/35636.

49. Zilverstand A, Sorger B, Slaats-Willemse D, Kan CC, Goebel R, Buitelaar JK. Fmri Neurofeedback Training for Increasing Anterior Cingulate Cortex Activation in Adult Attention Deficit Hyperactivity Disorder. An Exploratory Randomized, Single-Blinded Study. *Plos One* (2017) 12(1). doi: 10.1371/journal.pone.0170795.

50. Barham H, Buyukgok D, Aksu S, Soyata AZ, Bulut G, Eskicioglu G, et al. Evidence for Modulation of Planning and Working Memory Capacities by Transcranial Direct Current Stimulation in a Sample of Adults with Attention Deficit Hyperactivity Disorder. *Neuroscience letters* (2022) 790:136883. doi: <https://dx.doi.org/10.1016/j.neulet.2022.136883>.

51. Bleich-Cohen M, Gurevitch G, Carmi N, Medvedovsky M, Bregman N, Nevler N, et al. A Functional Magnetic Resonance Imaging Investigation of Prefrontal Cortex Deep Transcranial Magnetic Stimulation Efficacy in Adults with Attention Deficit/Hyperactive Disorder: A Double Blind, Randomized Clinical Trial. *Neuroimage Clin* (2021) 30:102670. Epub 2021/07/04. doi: 10.1016/j.nicl.2021.102670.

52. Bloch Y, Harel EV, Aviram S, Govezensky J, Ratzoni G, Levkovitz Y. Positive Effects of Repetitive Transcranial Magnetic Stimulation on Attention in Adhd Subjects: A Randomized Controlled Pilot Study. *The World Journal of Biological Psychiatry* (2010) 11(5-6):755-8. doi: <https://dx.doi.org/10.3109/15622975.2010.484466>.

53. Cachoeira CT, Leffa DT, Mittelstadt SD, Mendes LST, Brunoni AR, Pinto JV, et al. Positive Effects of Transcranial Direct Current Stimulation in Adult Patients with Attention-Deficit/Hyperactivity Disorder - a Pilot Randomized Controlled Study. *Psychiatry Research* (2017) 247:28-32. doi: 10.1016/j.psychres.2016.11.009.

54. Dallmer-Zerbe I, Popp F, Lam AP, Philipsen A, Herrmann CS. Transcranial Alternating Current Stimulation (Tacs) as a Tool to Modulate P300 Amplitude in Attention Deficit Hyperactivity Disorder (Adhd): Preliminary Findings. *Brain Topogr* (2020) 33(2):191-207. Epub 2020/01/25. doi: 10.1007/s10548-020-00752-x.

55. Dubreuil-Vall L, Gomez-Bernal F, Villegas AC, Cirillo P, Surman C, Ruffini G, et al. Transcranial Direct Current Stimulation to the Left Dorsolateral Prefrontal Cortex Improves Cognitive Control in Patients with Attention-Deficit/Hyperactivity Disorder: A Randomized Behavioral and Neurophysiological Study. *Biol Psychiatry Cogn Neurosci Neuroimaging* (2021) 6(4):439-48. Epub 2021/02/08. doi: 10.1016/j.bpsc.2020.11.006.

56. Fu Z, Tian J, Kang S, Qin Z, Cao Q, Wang Y, et al. Efficacy and Safety of Transcranial Alternating Current Stimulation in Adults with Attention Deficit Hyperactivity Disorder: A Double-Blind Randomized Sham-Controlled Trial. *Molecular Psychiatry* (2025). Epub 20251219. doi: 10.1038/s41380-025-03407-0.

57. Kannen K, Rasbach J, Fantazi A, Wiebe A, Selaskowski B, Asché L, et al. Alpha Modulation Via Transcranial Alternating Current Stimulation in Adults with Attention-Deficit Hyperactivity Disorder. *Frontiers in Psychology* (2024) 14. doi: 10.3389/fpsyg.2023.1280397.

58. Leffa DT, Grevet EH, Bau CHD, Schneider M, Ferrazza CP, da Silva RF, et al. Transcranial Direct Current Stimulation Vs Sham for the Treatment of Inattention in Adults with Attention-Deficit/Hyperactivity Disorder: The Tuned Randomized Clinical Trial. *JAMA Psychiatry* (2022) 79(9):847-56. Epub 2022/08/04. doi: 10.1001/jamapsychiatry.2022.2055.

59. Paz Y, Friedwald K, Levkovitz Y, Zangen A, Alyagon U, Nitzan U, et al. Randomised Sham-Controlled Study of High-Frequency Bilateral Deep Transcranial Magnetic Stimulation (Dtms) to Treat Adult Attention Hyperactive Disorder (Adhd): Negative Results. *World J Biol Psychiatry* (2018) 19(7):561-6. Epub 2017/01/17. doi: 10.1080/15622975.2017.1282170.

60. Rodrigues da Silva PH, Leffa DT, Luethi MS, Silva RF, Ferrazza CP, Picon FA, et al. Baseline Brain Volume Predicts Home-Based Transcranial Direct Current Stimulation Effects on Inattention in Adults with Attention-Deficit/Hyperactivity Disorder. *J Psychiatr Res* (2024) 177:403-11. doi: doi:10.1016/j.jpsychires.2024.07.042.

61. Schneider M, Ferrazza CP, da Silva Bomber RF, Picon F, Rovaris DL, Sanches PRS, et al. Enhancing Goal Achievement in Adults with Adhd: A Participant-Centered Evaluation of Transcranial Direct Current Stimulation from the Tuned Trial. *J Atten Disord* (2025) 29(12):1070-8. Epub 20250617. doi: 10.1177/10870547251341595.

62. Bartlett G, Frings D, Chaplin E. Evaluating Doppel's Impact on Anxiety and Focus Amongst Adults with Adhd. *PLOS Digit Health* (2024) 3(7):e0000555. doi: doi:10.1371/journal.pdig.0000555.

63. Praus P, Proctor T, Rohrmann T, Benedyk A, Tost H, Hennig O, et al. Female Sex and Burden of Depressive Symptoms Predict Insufficient Response to Telemedical Treatment in Adult Attention-Deficit/Hyperactivity Disorder: Results from a Naturalistic Patient Cohort during the Covid-19 Pandemic. *Front Psychiatry* (2023) 14. doi: 10.3389/fpsyt.2023.1193898.

64. Adamou M, Jones SL, Marks L, Lowe D. Efficacy of Continuous Performance Testing in Adult Adhd in a Clinical Sample Using Qbtest+. *J Atten Disord* (2022) 26(11):1483-91. doi: 10.1177/10870547221079798.

65. Amar LA, Otifi AM, Mohamed SA. Comparative Study of Multi-Headed and Baseline Deep Learning Models for Adhd Classification from Eeg Signals. *Physical and engineering sciences in medicine* (2025) 48(4):1657-65. doi: <https://dx.doi.org/10.1007/s13246-025-01609-y>.

66. Baghdassarian EJ, Markhed MN, Lindström E, Nilsson BM, Lewander T. Auditory Brainstem Response (Abr) Profiling Tests as Diagnostic Support for Schizophrenia and Adult Attention-Deficit Hyperactivity Disorder (Adhd). *Acta Neuropsychiatrica* (2018) 30(3):137-47. doi: 10.1017/neu.2017.24.

67. Bijlenga D, Jasperse M, Gehlhaar SK, Kooij JJS. Objective Qbtest and Subjective Evaluation of Stimulant Treatment in Adult Attention Deficit-Hyperactivity Disorder. *Eur Psychiat* (2015) 30(1). doi: 10.1016/j.eurpsy.2014.06.003.

68. Bijlenga D, Ulberstad F, Thorell LB, Christiansen H, Hirsch O, Kooij JJS. Objective Assessment of Attention-Deficit/Hyperactivity Disorder in Older Adults Compared with Controls Using the Qbtest. *Int J Geriatr Psychiatry* (2019) 34(10):1526-33. Epub 2019/06/28. doi: 10.1002/gps.5163.

69. Chaim-Avancini TM, Doshi J, Zanetti MV, Erus G, Silva MA, Duran FLS, et al. Neurobiological Support to the Diagnosis of Adhd in Stimulant-Naive Adults: Pattern Recognition Analyses of Mri Data. *Acta Psychiatrica Scandinavica* (2017) 136(6):623-36. doi: <https://dx.doi.org/10.1111/acps.12824>.

70. Chen T, Antoniou G, Adamou M, Tachmazidis I, Su P. Automatic Diagnosis of Attention Deficit Hyperactivity Disorder Using Machine Learning. *Applied Artificial Intelligence* (2021) 35(9):657-69. doi: <https://dx.doi.org/10.1080/08839514.2021.1933761>.

71. Edebol H, Helldin L, Norlander T. Measuring Adult Attention Deficit Hyperactivity Disorder Using the Quantified Behavior Test Plus. *PsyCh Journal* (2013) 2(1):48-62. doi: 10.1002/pchj.17.

72. Elbaum T, Braw Y, Lev A, Rassovsky Y. Attention-Deficit/Hyperactivity Disorder (Adhd): Integrating the Moxo-Dcpt with an Eye Tracker Enhances Diagnostic Precision. *Sensors (Basel)* (2020) 20(21). Epub 2020/11/14. doi: 10.3390/s20216386.

73. Emser TS, Johnston BA, Steele JD, Kooij S, Thorell L, Christiansen H. Assessing Adhd Symptoms in Children and Adults: Evaluating the Role of Objective Measures. *Behavioral and Brain Functions* (2018) 14(1). doi: 10.1186/s12993-018-0143-x.

74. Gounari KA, Giatzoglou E, Kemm R, Beratis IN, Nega C, Kourtesis P. The Trail Making Test in Virtual Reality (Tmt-Vr): Examination of the Ecological Validity, Usability, Acceptability, and User Experience in Adults with Adhd. *Psychiatry International* (2025) 6(1):31. doi: 10.3390/psychiatryint6010031.

75. Groom MJ, Young Z, Hall CL, Gillott A, Hollis C. The Incremental Validity of a Computerised Assessment Added to Clinical Rating Scales to Differentiate Adult Adhd from Autism Spectrum Disorder. *Psychiatry Res* (2016) 243:168-73. Epub 2016/07/12. doi: 10.1016/j.psychres.2016.06.042.

76. Herman BK, Faraone SV, Cutler AJ, Newcorn JH, LaFrance EM, Ripper Lewis M, et al. Validity of an Online Assessment of Attention-Deficit/Hyperactivity Disorder among a Real-World Sample of Adults Seeking Web-Based Mental Health Care. *J Clin Psychiatry* (2025) 86(3). Epub 20250908. doi: 10.4088/JCP.25m15846.

77. Jylkkä J, Ritakallio L, Merzon L, Kangas S, Kliegel M, Zuber S, et al. Assessment of Goal-Directed Behavior and Prospective Memory in Adult Adhd with an Online 3d Videogame Simulating Everyday Tasks. *Sci Rep* (2023) 13(1):9299. Epub 2023/06/09. doi: 10.1038/s41598-023-36351-6.

78. Kaur S, Arun P, Singh S, Kaur D, editors. Eeg Based Decision Support System to Diagnose Adults with Adhd. *2018 IEEE Applied Signal Processing Conference (ASPCON)*; 2018 7-9 Dec. 2018.

79. Kaur S, Singh S, Arun P, Kaur D, Bajaj M. Phase Space Reconstruction of Eeg Signals for Classification of Adhd and Control Adults. *Clin EEG Neurosci* (2020) 51(2):102-13. Epub 2019/09/20. doi: 10.1177/1550059419876525.

80. Kim S, Baek JH, Kwon YJ, Lee HY, Yoo JH, Shim S-H, et al. Machine-Learning-Based Diagnosis of Drug-Naive Adult Patients with Attention-Deficit Hyperactivity Disorder Using Mismatch Negativity. *Translational psychiatry* (2021) 11(1):484. doi: <https://dx.doi.org/10.1038/s41398-021-01604-3>.

81. Lev A, Braw Y, Elbaum T, Wagner M, Rassovsky Y. Eye Tracking during a Continuous Performance Test: Utility for Assessing Adhd Patients. *J Atten Disord* (2022) 26(2):245-55. Epub 2020/11/27. doi: 10.1177/1087054720972786.

82. Li S, Nair R, Naqvi M. Acoustic and Text Features Analysis for Adult Adhd Screening: A Data-Driven Approach Utilizing Diva Interview. *IEEE Journal of Translational Engineering in Health and Medicine* (2024):1-. doi: 10.1109/JTEHM.2024.3369764.

83. Lim S, Dong SY, McIntyre RS, Chiang SK, Ho R. Exploring Functional Connectivity in Attention Deficit/Hyperactivity Disorder: A Functional near-Infrared Spectroscopy Study with Machine Learning Analysis. *IEEE J Biomed Health Inform* (2025):1-13. doi: 10.1109/JBHI.2025.3564487.

84. Luo Y, Alvarez TL, Halperin JM, Li X. Multimodal Neuroimaging-Based Prediction of Adult Outcomes in Childhood-Onset Adhd Using Ensemble Learning Techniques. *Neuroimage Clin* (2020) 26:102238. Epub 2020/03/18. doi: 10.1016/j.nicl.2020.102238.

85. Mueller A, Candrian G, Grane VA, Kropotov JD, Ponomarev VA, Baschera G-M. Discriminating between Adhd Adults and Controls Using Independent Erp Components and a Support Vector Machine: A Validation Study. *Nonlinear Biomedical Physics* (2011) 5(1):5. doi: 10.1186/1753-4631-5-5.

86. Muller A, Vetsch S, Pershin I, Candrian G, Baschera G-M, Kropotov JD, et al. Eeg/Erp-Based Biomarker/Neuroalgorithms in Adults with Adhd: Development, Reliability, and Application in Clinical Practice. *The world journal of biological psychiatry : the official journal of the World Federation of Societies of Biological Psychiatry* (2020) 21(3):172-82. doi: <https://dx.doi.org/10.1080/15622975.2019.1605198>.

87. Nash C, Nair R, Naqvi SM. Optimising Adhd Detection: An Autoencoder Approach for Multimodal Classification. *IEEE Transactions on Artificial Intelligence* (2025):1-11. doi: 10.1109/tai.2025.3592157.

88. Nobukawa S, Shirama A, Takahashi T, Takeda T, Ohta H, Kikuchi M, et al. Identification of Attention-Deficit Hyperactivity Disorder Based on the Complexity and Symmetricity of Pupil Diameter. *Sci Rep* (2021) 11(1):8439. Epub 2021/04/21. doi: 10.1038/s41598-021-88191-x.

89. Oh HK, Cho YJ, Kim JJ, Shin B, Kim SJ, Park S, et al. Advancing Ecological Validity and Clinical Utility in Virtual Reality-Based Continuous Performance Test: Exploring the Effects of Task Difficulty and Environmental Distractors. *Front Psychiatry* (2023) 14. doi: 10.3389/fpsyt.2023.1329221.

90. Park BY, Kim M, Seo J, Lee JM, Park H. Connectivity Analysis and Feature Classification in Attention Deficit Hyperactivity Disorder Sub-Types: A Task Functional Magnetic Resonance Imaging Study. *Brain Topogr* (2016) 29(3):429-39. Epub 2015/11/26. doi: 10.1007/s10548-015-0463-1.

91. Robeva R, Penberthy JK, Loboschefski T, Cox D, Kovatchev B. Combined Psychophysiological Assessment of Adhd: A Pilot Study of Bayesian Probability Approach Illustrated by Appraisal of Adhd in Female College Students. *Applied Psychophysiology and Biofeedback* (2004) 29(1):1-18. doi: 10.1023/b:apbi.0000017860.60164.66.

92. Schweiger A, Abramovitch A, Doniger GM, Simon ES. A Clinical Construct Validity Study of a Novel Computerized Battery for the Diagnosis of Adhd in Young Adults. *J Clin Exp Neuropsychol* (2007) 29(1):100-11. Epub 2006/12/13. doi: 10.1080/13803390500519738.

93. Ulberstad F, Bostrom H, Chavanon M-L, Knollmann M, Wiley J, Christiansen H, et al. Objective Measurement of Attention Deficit Hyperactivity Disorder Symptoms Outside the Clinic Using the Qbcheck: Reliability and Validity. *International journal of methods in psychiatric research* (2020) 29(2):e1822. doi: <https://dx.doi.org/10.1002/mpr.1822>.

94. von Polier GG, Ahlers E, Volkening J, Langner J, Patil KR, Eickhoff SB, et al. Exploring Voice as a Digital Phenotype in Adults with Adhd. *Scientific Reports* (2025) 15(1):18076. Epub 20250524. doi: 10.1038/s41598-025-01989-x.

95. Wiebe A, Kannen K, Li M, Aslan B, Anders D, Selaskowski B, et al. Multimodal Virtual Reality-Based Assessment of Adult Adhd: A Feasibility Study in Healthy Subjects. *Assessment* (2023) 30(5):1435-53. Epub 2022/04/19. doi: 10.1177/10731911221089193.

96. Wiebe A, Aslan B, Brockmann C, Lepartz A, Dudek D, Kannen K, et al. Multimodal Assessment of Adult Attention-Deficit Hyperactivity Disorder: A Controlled Virtual Seminar Room Study. *Clinical Psychology & Psychotherapy* (2023). doi: 10.1002/cpp.2863.

97. Wiebe A, Selaskowski B, Paskin M, Asché L, Pakos J, Aslan B, et al. Virtual Reality-Assisted Prediction of Adult Adhd Based on Eye Tracking, Eeg, Actigraphy and Behavioral Indices: A Machine Learning Analysis of Independent Training and Test Samples. *Translational Psychiatry* (2024) 14(1). doi: 10.1038/s41398-024-03217-y.

98. Yao D, Sun H, Guo X, Calhoun VD, Sun L, Sui J, editors. Adhd Classification within and Cross Cohort Using an Ensembled Feature Selection Framework. *2019 IEEE 16th International Symposium on Biomedical Imaging (ISBI 2019)*; 2019 8-11 April 2019.

99. Yousefimehr B, Ghatee M, Heydari A, editors. Improving Adhd Detection with Cost-Sensitive Lightgbm. *14th International Conference on Computer and Knowledge Engineering (ICCKE)*; 2024 2024-11-20.

100. Abedian S, Bajestani GS, Saeedi H, Makhloughi F. Diagnosis of Adult Adhd Using Eeg Signals Based on the Spectrogram and Convolutional Neural Networks. *Int J Comput Intell Appl* (2024). doi: 10.1142/s1469026823500347.

101. Ghassemi F, Moradi MH, Tehrani-Doost M, Abootalebi V, editors. Classification of Adhd/Normal Participants Using Frequency Features of Erp's Independent Components. *2010 17th Iranian Conference of Biomedical Engineering (ICBME)*; 2010 3-4 Nov. 2010.

102. Hong M, Dong SY, McIntyre RS, Chiang SK, Ho R. Fnirs Classification of Adults with Adhd Enhanced by Feature Selection. *IEEE Trans Neural Syst Rehabil Eng* (2024):220-31. doi: 10.1109/TNSRE.2024.3522121.

103. Jayawardena G, Michalek A, Jayarathna S, editors. Eye Tracking Area of Interest in the Context of Working Memory Capacity Tasks. *2019 IEEE 20th International Conference on Information Reuse and Integration for Data Science (IRI)*; 2019 30 July-1 Aug. 2019.

104. Kiiski H, Rueda-Delgado LM, Bennett M, Knight R, Rai L, Roddy D, et al. Functional Eeg Connectivity Is a Neuromarker for Adult Attention Deficit Hyperactivity Disorder Symptoms. *Clinical Neurophysiology* (2020) 131(1):330-42. doi: 10.1016/j.clinph.2019.08.010.

105. Leontyev A, Yamauchi T, Razavi M, editors. Machine Learning Stop Signal Test (Ml-Sst): Ml-Based Mouse Tracking Enhances Adult Adhd Diagnosis. *2019 8th International Conference on Affective Computing and Intelligent Interaction Workshops and Demos (ACIIW)*; 2019 3-6 Sept. 2019.

106. Liu L, Tang S, Wu F-X, Wang Y-P, Wang J. An Ensemble Hybrid Feature Selection Method for Neuropsychiatric Disorder Classification. *IEEE/ACM Trans Comput Biol Bioinformatics* (2021) 19(3):1459–71. doi: 10.1109/tcbb.2021.3053181.

107. Ma G, Eng AE, Chiang SK, Hao F, McIntyre RS, Zhou D, et al. A Comparative Diagnostic Study Using Clinical and Multimodal Assessment, Including Functional Neuroimaging and Oculomotricity Tools, to Differentiate Adhd in Young Patients from Healthy Control Group. *Psychiatry Clin Neurosci* (2025) 79(4):165-75. doi: 10.1111/pcn.13788.

108. Morey LC. Examining a Novel Performance Validity Task for the Detection of Feigned Attentional Problems. *Appl Neuropsychol Adult* (2019) 26(3):255-67. Epub 2017/12/19. doi: 10.1080/23279095.2017.1409749.

109. Namasse Z, Tabaa M, Hidila Z, Mouchawrab S. Explainable Artificial Intelligence for Predicting Attention Deficit Hyperactivity Disorder in Children and Adults. *Healthcare (Basel)* (2025) 13(2). doi: 10.3390/healthcare13020155.

110. Taymourtash A, Ghassemi F, editors. Independent Component Analysis of Sparse-Transformed Eeg Signals for Adhd/Normal Adults' Classification. *2015 23rd Iranian Conference on Electrical Engineering*; 2015 10-14 May 2015.

111. Tenev A, Markovska-Simoska S, Kocarev L, Pop-Jordanov J, Müller A, Candrian G. Machine Learning Approach for Classification of Adhd Adults. *Int J Psychophysiol* (2014) 93(1):162-6. Epub 2013/01/31. doi: 10.1016/j.ijpsycho.2013.01.008.

112. Torgersen EL, Ragnarson I, Molinas M. Decoding Attention through Eeg: Paving the Way for Bci Applications in Attention-Related Disorders. *Annual International Conference of the IEEE Engineering in Medicine and Biology Society* (2025) 2025:1-7. doi: 10.1109/EMBC58623.2025.11252840.

113. Trinh N, Whelan R, Ward T, Derosiere G, editors. Task-Related and Resting-State Eeg Classification of Adult Patients with Adhd Using Machine Learning. *2023 IEEE 19th International Conference on Body Sensor Networks (BSN)*; 2023 9-11 Oct. 2023.

114. Unal M, O'Mahony E, Dunne C, Meagher D, Adamis D. The Clinical Utility of Three Visual Attention Tests to Distinguish Adults with Adhd from Normal Controls. *Rivista Di Psichiatria* (2019) 54(5):211-7. doi: 10.1708/3249.32185.

115. Wang X, Jiao Y, Lu Z, editors. Discriminative Analysis of Resting-State Brain Functional Connectivity Patterns of Attention-Deficit Hyperactivity Disorder Using Kernel Principal Component Analysis. *2011 Eighth International Conference on Fuzzy Systems and Knowledge Discovery (FSKD)*; 2011 26-28 July 2011.

116. Yao DR, Yang EK, Sun L, Sui J, Liu MX, editors. Integrating Multimodal Mris for Adult Adhd Identification with Heterogeneous Graph Attention Convolutional Network. *4th International Workshop on Predictive Intelligence in Medicine (PRIME)*; 2021 Oct 01; Strasbourg, FRANCE (2021).

117. Zhang H, Zeng W, Deng J, Shi Y, Zhao L, Li Y. Brain Relatively Inert Network: Taking Adult Attention Deficit Hyperactivity Disorder as an Example. *Front Neurosci* (2021) 15:771947. Epub 2021/12/21. doi: 10.3389/fnins.2021.771947.

118. Zhang Y, Ran M, Zhang X, Wang M, Jiang G. Diminished Prefrontal Hemodynamic Response in Adult Attention Deficit Hyperactivity Disorder: A Multi-Channel Functional near-Infrared Spectroscopy Study. *Eur J Psychiat* (2023) 37(3):160-6. doi: 10.1016/j.ejpsy.2023.02.001.

119. Backer A, Forsstrom D, Hommerberg L, Johansson M, Hensler I, Lindner P. A Novel Self-Rating Instrument Designed for Long-Term, App-Based Monitoring of Adhd Symptoms: A Mixed-Methods Development and Validation Study. *Digit Health* (2024) 10:20552076241280037. doi: 10.1177/20552076241280037.

120. Biederman J, Fried R, DiSalvo M, Driscoll H, Green A, Biederman I, et al. A Novel Digital Health Intervention to Improve Patient Engagement to Stimulants in Adult Adhd in the Primary Care Setting: Preliminary Findings from an Open Label Study. *Psychiatry Res* (2020) 291:113158. Epub 2020/06/20. doi: 10.1016/j.psychres.2020.113158.

121. Carvalho LR, Haas LM, Zeni G, Victor MM, Techele SP, Marrone Castanho J, et al. Evaluation of the Effectiveness of the Focus Adhd App in Monitoring Adults with Attention-Deficit/Hyperactivity Disorder. *Eur Psychiat* (2023) 66(1):e53. doi: 10.1192/j.eurpsy.2023.2422.

122. Dan M, Grabinski MJ, Raiff BR. Smartphone-Based Contingency Management for Smoking Cessation with Smokers Diagnosed with Attention-Deficit/Hyperactivity Disorder. *Special Issue: Incentives and Motivation* (2016) 2(2):116-27. doi: <https://dx.doi.org/10.1037/tps0000062>.

123. Håvik R, Wake JD, Flobak E, Lundervold A, Guribye F, editors. A Conversational Interface for Self-Screening for Adhd in Adults. *5th International Conference on Internet Science (INSCI)*; 2018 Oct 24-26; St. Petersburg, RUSSIA (2019).

124. Sankesara H, Denyer H, Sun S, Deng Q, Ranjan Y, Conde P, et al. Identifying Digital Markers of Attention-Deficit/Hyperactivity Disorder (Adhd) in a Remote Monitoring Setting: Prospective Observational Study. *JMIR formative research* (2025) 9:e54531. Epub 20250129. doi: 10.2196/54531.

125. Surman C, Boland H, Kaufman D, DiSalvo M. Personalized Remote Mobile Surveys of Adult Adhd Symptoms and Function: A Pilot Study of Usability and Utility for Pharmacology Monitoring. *J Atten Disord* (2022) 26(7):1001-10. Epub 2021/10/26. doi: 10.1177/10870547211044213.

126. Patrickson B, Shams L, Fouyaxis J, Strobel J, Schubert KO, Musker M, et al. Evolving Adult Adhd Care: Preparatory Evaluation of a Prototype Digital Service Model Innovation for Adhd Care. *International Journal of Environmental Research and Public Health* (2024) 21(5):582. doi: 10.3390/ijerph21050582.

127. Ware S, Rijal K, Knouse LE, editors. Smartadhdmonitor: A Novel Approach to Automatic Adhd Monitoring through Smartphone App Usage Data. *2025 IEEE 49th Annual Computers, Software, and Applications Conference (COMPSAC)*; 2025 2025.

128. Jang S, Kim J-J, Kim S-J, Hong J, Kim S, Kim E. Mobile App-Based Chatbot to Deliver Cognitive Behavioral Therapy and Psychoeducation for Adults with Attention Deficit: A Development and Feasibility/Usability Study. *International journal of medical informatics* (2021) 150:104440. doi: <https://dx.doi.org/10.1016/j.ijmedinf.2021.104440>.

129. Lindstedt H, Umb-Carlsson O. Cognitive Assistive Technology and Professional Support in Everyday Life for Adults with Adhd. *Disabil Rehabil Assist Technol* (2013) 8(5):402-8. Epub 2013/09/03. doi: 10.3109/17483107.2013.769120.

130. Luiu AL, Prada P, Perroud N, Lovis C, Ehrler F. Adhd Mobile App Feasibility Test for Adults. *Stud Health Technol Inform* (2018) 255:247-51. Epub 2018/10/12.

131. Seery C, Cochrane RH, Mulcahy M, Kilbride K, Wrigley M, Bramham J. "A One-Stop Shop": Real-World Use and App-Users' Experiences of a Psychoeducational Smartphone App for Adults with Adhd. *Internet Interv* (2025) 39:100807. doi: 10.1016/j.invent.2025.100807.

132. Store SJ, Tillfors M, Angelhoff C, Norell-Clarke A. A Robot Intervention for Adults with Adhd and Insomnia-a Mixed-Method Proof-of-Concept Study. *PloS one* (2023) 18(9):e0290984. doi: <https://dx.doi.org/10.1371/journal.pone.0290984>.

133. Adamou M, Jones SL, Fullen T, Galab N, Abbott K, Yasmeen S. Remote Assessment in Adults with Autism or Adhd: A Service User Satisfaction Survey. *PLoS One* (2021) 16(3):e0249237. Epub 2021/03/26. doi: 10.1371/journal.pone.0249237.
